# Levels of human proteins in plasma associated with acute paediatric malaria

**DOI:** 10.1186/s12936-018-2576-y

**Published:** 2018-11-15

**Authors:** Philippa Reuterswärd, Sofia Bergström, Judy Orikiiriza, Elisabeth Lindquist, Sven Bergström, Helene Andersson Svahn, Burcu Ayoglu, Mathias Uhlén, Mats Wahlgren, Johan Normark, Ulf Ribacke, Peter Nilsson

**Affiliations:** 1 Department of Protein Science, SciLifeLab, KTH Royal Institute of Technology, Stockholm, Sweden; 2 Infectious Diseases Institute, College of Health Sciences, Makerere University, Kampala, Uganda; 3 Department of Molecular Biology, Umeå University, Umeå, Sweden; 4 Department of Medicine, Division of Immunology and Rheumatology, School of Medicine, Stanford University, Stanford, CA 94305 USA; 5 Department of Microbiology, Tumor and Cell Biology, Karolinska Institutet, Stockholm, Sweden

**Keywords:** Affinity proteomics, Human plasma profiling, Malaria, *Plasmodium falciparum*, suspension bead arrays, Sequestration, Cytoadhesion

## Abstract

**Background:**

The intimate interaction between the pathophysiology of the human host and the biology of the *Plasmodium falciparum* parasite results in a wide spectrum of disease outcomes in malaria. Development of severe disease is associated with a progressively augmented imbalance in pro- and anti-inflammatory responses to high parasite loads and sequestration of parasitized erythrocytes. Although these phenomena collectively constitute common denominators for the wide variety of discrete severe malaria manifestations, the mechanistic rationales behind discrepancies in outcome are poorly understood. Exploration of the human pathophysiological response by variations in protein profiles in plasma presents an excellent opportunity to increase the understanding. This is ultimately required for better prediction, prevention and treatment of malaria, which is essential for ongoing elimination and eradication efforts.

**Results:**

An affinity proteomics approach was used to analyse 541 paediatric plasma samples collected from community controls and patients with mild or severe malaria in Rwanda. Protein profiles were generated with an antibody-based suspension bead array containing 255 antibodies targetting 115 human proteins. Here, 57 proteins were identified with significantly altered levels (adjusted p-values < 0.001) in patients with malaria compared to controls. From these, the 27 most significant proteins (adjusted p-values < 10^−14^) were selected for a stringent analysis approach. Here, 24 proteins showed elevated levels in malaria patients and included proteins involved in acute inflammatory response as well as cell adhesion. The remaining three proteins, also implicated in immune regulation and cellular adhesivity, displayed lower abundance in malaria patients. In addition, 37 proteins (adjusted p-values < 0.05) were identified with increased levels in patients with severe compared to mild malaria. This set includes, proteins involved in tissue remodelling and erythrocyte membrane proteins. Collectively, this approach has been successfully used to identify proteins both with known and unknown association with different stages of malaria.

**Conclusion:**

In this study, a high-throughput affinity proteomics approach was used to find protein profiles in plasma linked to *P. falciparum* infection and malaria disease progression. The proteins presented herein are mainly involved in inflammatory response, cellular adhesion and as constituents of erythrocyte membrane. These findings have a great potential to provide increased conceptual understanding of host-parasite interaction and malaria pathogenesis.

**Electronic supplementary material:**

The online version of this article (10.1186/s12936-018-2576-y) contains supplementary material, which is available to authorized users.

## Background

Severe malaria caused by *Plasmodium falciparum* continues to be one of the leading infectious causes of morbidity and mortality in children worldwide [1, 2]. At first, the infection is most often manifested as an uncomplicated illness with non-specific symptomatology before it can rapidly progress into severe malaria with the majority of deaths occurring within 24 h of admission [3–5]. Importantly though, although the number of severe cases and deaths due to the disease is high, the absolute majority of patients never reach those stages in disease progression. At present, the understanding of the underlying, highly variable disease pathogenesis is limited.

The outcome of the disease is a result of the intimate interaction between the biology of the parasite and the pathophysiology of the human host during an on-going infection. Certain aspects of the host pathophysiology are well-known. For example, the early stages of clinical illness are known to be characterized by an imbalance in pro- and anti-inflammatory cytokines as a response to parasitized erythrocytes [6]. Increasing parasite loads further augment this imbalance. This mirrors what is often seen during the acute phase response to other types of infections, with elevated plasma levels of molecules such as c-reactive protein (CRP), lipopolysaccharide binding protein (LBP) and the cytokines tumour necrosis factor (TNF), interleukin 10 (Il-10) and interferon-gamma, among others [7–11]. As the *P. falciparum* malaria progresses to the severe form, this imbalance becomes even more profound, pointing to an exaggerated human immune response as one key aspect in the pathogenesis of the disease.

Severe and fatal malaria has also been intrinsically linked to the sequestration of *P. falciparum* parasitized erythrocytes to platelets, uninfected erythrocytes and endothelium of the peripheral microvasculature [12]. Sequestration of high parasite loads in various vital organs, in concert with the pathophysiological response of the host, are major reasons for the complexity of the severe malaria syndrome, with potential multi-organ consequences and failures. Besides the resulting physical blockade of oxygen transport, this sequestration leads to activation of the endothelium resulting in further inflammatory response, dysregulation of coagulation as well as release of microparticles of both endothelial, platelet and erythrocyte origin [13, 14]. In addition, the metabolism of the sequestered parasites also results in alterations in metabolic pathways of the affected organs and the resulting complications are increasingly recognized as contributors to tissue damage and severe disease [15, 16].

Thus, certain factors involved in the interaction between the parasite and the host and how these collectively contribute to the *P. falciparum* malaria pathogenesis are known. There is however still a major gap in the understanding of both general and specific mechanisms behind the appearance of uncomplicated malaria and in particular the various and discrete forms of complications presented in severely ill patients [17–20]. Identification and exploration of these mechanisms, with a consequential improved understanding of the pathogenesis, is vital in order to predict and prevent the disease to occur. This, in turn, is essential for ongoing malaria eradication efforts.

High-throughput screens of host protein levels in plasma present unprecedented opportunities to identify human proteins associated with disease pathogenesis. A previous in-house study by Bachmann et al. used suspension bead arrays and more than 1000 antibodies from the Human Protein Atlas (http://www.proteinatlas.org) to identify a protein panel discriminating patients with uncomplicated and different categories of severe malaria in a cohort of 700 paediatric samples from Nigeria [21]. Here the aim is to further increase the conceptual understanding of the interaction between the parasite and the human host. To achieve this, a targetted affinity-based proteomics approach was employed to profile 541 plasma samples from a paediatric cohort collected in Rwanda. With this approach, several of the malaria-associated proteins previously found by Bachmann et al. [21] have been identified alongside well-known proteins that display differential levels at different stages of the infection, as well as new leads for the exploration of parasite virulence and human pathophysiology in malaria.

## Method

**Fig. 1:**
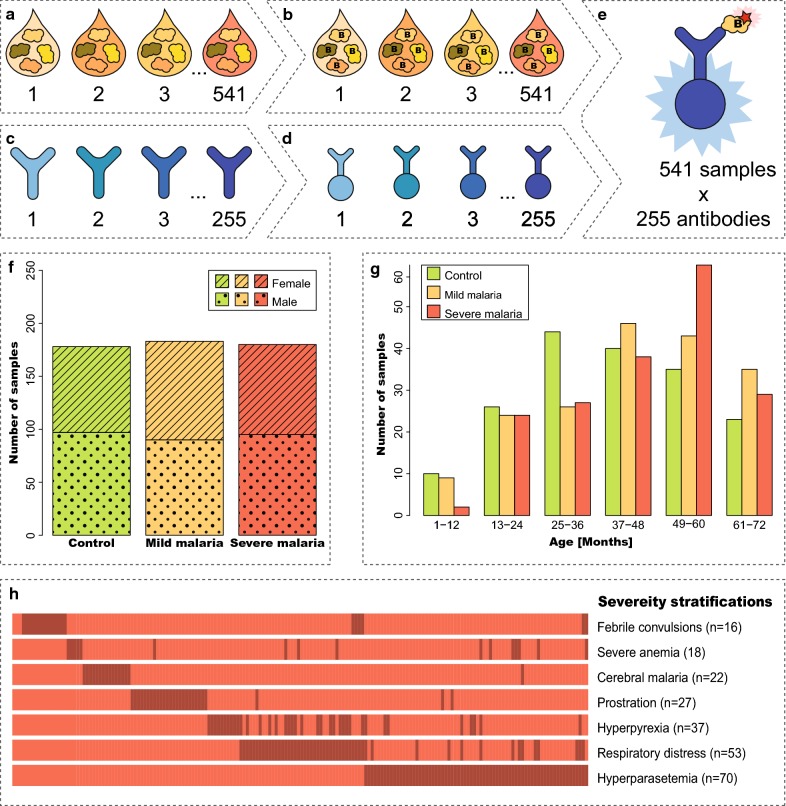
Overview of the antibody suspension bead array workflow and sample demographics. **a** Plasma samples were distributed into plates. **b** Samples were directly labelled with biotin. **c** 255 antibodies were diluted and distributed into plates. **d** Antibodies were coupled to magnetic, colour-coded beads, each with an individual bead ID. **e** 255 labelled samples were incubated with antibody-coupled beads. The beads were subsequently incubated together with a streptavidin-conjugated fluorophore. Two assay plates were used in this study since 541 samples were included. Detection was made using a flow cytometer-like instrument, registering signal intensity and bead ID by the utilization of two different lasers. **f** Information about sample groups and sex distribution within each sample group. **g** Age distributions were outlined for each sample group. **h** Different severe disease stratifications per sample within the severe malaria group illustrated as a line in the legend. A number (n = 49) of the samples exhibit more than one severe disease manifestation (see also Additional file 1)

Antibody suspension bead arrays were used to screen a cohort of paediatric plasma samples in order to explore human proteins involved in malaria pathogenesis. By combining previously identified disease-associated proteins with additions of proteins from a hypothesis-driven approach, 115 proteins were targetted with 255 antibodies (Fig. 1a–e). The sample cohort consisted of 541 plasma samples from community controls, mild malaria patients and various stratifications of severe malaria patients (Fig. 1f–h).

### Sample cohort

Plasma samples from paediatric malaria patients, as well as community controls, were collected in the regional hospital as well as health centres in the catchment area of Nyagatare, Eastern Province, Rwanda. The patients were enrolled over a 3-year period, that begun in 2011 and ended in 2014. The attending paediatrician or research assistant recorded clinical and biometric parameters at the sample collection site. The clinical assessment of the disease stratifications was performed simultaneously as the samples were drawn, i.e. at the emergency room as the patients received initial triage and supportive care. The children enrolled in the study were aged from 3 months up to 6 years. Community controls were recruited from healthy siblings or other children in the villages where the patients lived. In total, 541 samples were analysed with associated clinical data, including 178 controls, 183 patients with mild malaria and 180 patients with severe malaria. Clinical patient characteristics and the different severe disease stratifications are present in the group with severe malaria as detailed in the supplementary information (Additional files 1, 2). A positive blood smear was used to confirm active infection with *P. falciparum* malaria for patients with both mild and severe disease. All healthy community control patients were screened and found negative for malaria. The subtypes of severe malaria included in the study were hyperparasitaemia, respiratory distress, hyperpyrexia, prostration, cerebral malaria, malaria with severe anaemia, febrile convulsions, or combinations thereof. This the classification of severity was based on the WHO criteria for the identification of mild and different manifestations of severe malaria [22]. HIV positivity was an exclusion criterion and all known HIV positive patients were excluded from the initial screening process. All patients were subsequently screened for HIV and the study subjects found positive were excluded from the study. The attending clinician duly denoted any suspected co-infections in the clinical data.

Blood samples were collected at admission in ethylenediaminetetraacetic acid (EDTA) tubes. The blood was processed immediately after sampling from the patient into separate blood constituents by gel flotation (Polymorphoprep, Alere Technologies AS). The plasma samples were subsequently snap frozen in liquid nitrogen and stored at below − 80 °C until proteomic analysis was performed [22]. Plasma samples from the patients were directly labelled using biotin for the bead array analysis (Additional file 3A).

### Antibody selection

Antibodies for this screening were selected to validate previous findings by Bachmann et al. [21] in a new independent cohort, as well as for the discovery of new candidate protein markers to unravel the mechanism behind various stages of malaria infection. In addition to these previously suggested markers, additional proteins were selected from protein categories such as inflammatory proteins, erythrocyte surface proteins or proteins associated with microvesicles.

Overall, a total of 255 antibodies were included, targetting 115 unique human proteins, as well as assay specific controls. The majority of the antibodies used were polyclonal rabbit antibodies (Atlas Antibodies), originating from the Human Protein Atlas (HPA) (http://www.proteinatlas.org). In addition, 20 antibodies from R&D Systems, Abcam and Novus Biologicals were also included. Two assay specific controls were included in order to control the background; rabbit IgG (P120-301, Bethyl laboratories) for background binding to rabbit IgG molecules and a blocked bare bead (without coupled antibody) for background binding to the beads. Furthermore, two additional assay specific controls were used as sample loading controls; anti-human IgG (309-005-082, Jackson) and anti-human albumin (A0001, Dako). The selected antibodies were coupled onto carboxylated beads for the bead array assay (Additional file 3B).

### Assay procedure and readout

The assay procedure was performed as described previously [23, 24]. Biotin-labelled samples were thawed at 4 °C, vortexed and centrifuged at 2000 rpm for 1 min (Beckman Coulter). The plasma samples were diluted by adding 1 μL labelled plasma to 50 μL assay buffer in 96-well microtitre plates (732-4828, Thermo Scientific) using a liquid handler (SELMA, CyBio). The assay buffer consisted of 1× PBS with 0.5% (w/v) polyvinyl alcohol (P8136, Sigma-Aldrich), 0.8% (w/v) polyvinylpyrrolidone (PVP360, Sigma Life Science), 0.1% casein (C5890, Sigma Life Science) and 0.5 mg/ml rabbit IgG (P120-301, Bethyl Laboratories). The diluted samples were subsequently heat-treated at 56 °C for 30 min in a water bath (TW8, Julabo) followed by 10 min cooling down at room temperature. The samples (45 μL) were transferred to 5 μL bead stock in a 384-well microtitre assay plate, (781 101, Greiner BioOne), using the liquid handler before overnight incubation at 650 rpm at room temperature. Afterward, the beads were washed three times with 60 μL PBS-T and incubated for 10 min at room temperature at 650 rpm with 50 μL 0.4% paraformaldehyde (PFA) (43368, Alfa Aesar) in PBS-T. Beads were washed three times with 60 μL PBS-T before incubation with 50 μL of 0.5 μg/mL RPE-labelled streptavidin (SA1004-4, Invitrogen) for 20 min at room temperature at 650 rpm. Finally, the beads were washed three times with 60 μL PBS-T, before they were re-suspended in 60 μL PBS-T and analysed on a Flexmap 3D instrument (Luminex corp.). Binding events were displayed as median fluorescence intensity (MFI) where at least 50 beads per bead ID were counted. To assess inter-assay reproducibility, the assay run was repeated using a re-labelled subset (n = 325) of samples.

### Data analysis

Data analysis and visualization were performed with the open source software R (http://www.r-project.org) [25] and various R-packages, as described below.

As a part of quality control, inter- and intra-assay correlations were used to evaluate assay reproducibility using nonparametric Spearman’s correlation coefficients from the R-package ‘stats’. In order to explore sample heterogeneity, the distribution of the samples was illustrated with a nonlinear dimension reduction algorithm called t-distributed stochastic neighbour embedding (t-SNE) [26, 27]. The data was scaled and centred [28] before using the function Rtsne from the R-package ‘Rtsne’ with a perplexity of 30.

The univariate analysis included evaluation of individual protein’s ability to separate two sample groups. Student’s t-tests were used on log-transformed data for the analysis of the differences between controls and malaria cases (including both mild and severe malaria cases). Wilcoxon rank sum tests were used for the comparison between mild and severe malaria. False discovery rate (FDR) by Benjamini & Hochberg [29] was used to correct the p-values for multiple testing between controls versus malaria cases as well as mild versus severe malaria. Wilcoxon rank sum tests were used to calculate p-values for the pairwise comparisons of different severe disease stratifications (these p-values were not FDR adjusted). Boxplots of individual protein levels for different sample groups were created with R-package ‘graphics’ and ‘beeswarm’. Receiver operating characteristic (ROC) curves were used to demonstrate the classification power between two sample groups for the different proteins. Logistic regression was employed before ROC curves were created using log10-transformed data and the R-package ‘ROCR’.

Multivariate analysis was used to identify patterns in the data and to identify a potential combination of markers with higher separation power. Classification of protein level profiles was made using the unsupervised hierarchical clustering method Self-Organizing Tree Algorithm (SOTA) [30]. The data was scaled and centred [28] before using the R-package ‘clValid’ for SOTA clustering. In order to build a multivariate classification model the L1-penalized logistic regression, Least Absolute Shrinkage and Selection Operator (Lasso) [31] and logistic regression were combined. Lasso was applied using the R-package ‘glmnet’ to identify the driving variables in the dataset to evaluate potential multi-protein panels among all the antibodies cleared in the quality control. For the Lasso procedure, the MFI data were randomly divided into a training set and a test set. The training set consisted of 361 samples in total, including 119 control samples, 122 samples with mild malaria, and 120 samples with severe malaria. The test set consisted of 180 samples including; 59 control samples, 61 samples with mild malaria, and 60 samples with severe malaria. The tuning parameter lambda was determined by a five-fold cross-validation. The Lasso procedure determined the selection of contributing proteins using the value of lambda. Based on the output from Lasso, antibodies were selected to be included in a multi-protein panel. A model was built with this panel of antibodies using logistic regression on log10-transformed data. The classification power of the model was presented with ROC curves as described above and the area under the curve (AUC) was calculated, using the test set of samples. Heatmaps were used for exploratory analysis of samples and proteins using the R-package ‘pheatmap’ using hierarchical clustering with Euclidean distance on log10-transformed MFI data divided into two clusters.

Association networks between the proteins were explored using STRING v10.5 (https://string-db.org). The analysis was performed using all 61 presented proteins (Additional files 4, 5) as input and showed median confidence scores of minimum 0.4.

### Study and experimental quality control

Sample heterogeneity was explored using t-SNE plots to visualize how clinical data correlated with the protein profiles. Six parameters were selected for visualization (Additional file 6), based on the presented proteins (Additional files 4, 5). As expected, a visual trend in separation could be observed between controls and malaria samples for the following parameters: disease subtype, body temperature and signs of dehydration. No separation was observed for sex, age or weight.

During the assay analysis, MFI was registered for each antibody-coupled bead ID and sample. Coefficients of variation (CV) were calculated for all replicated samples to assess the technical variability of the assay. All median CVs were below 10% (IQR = 5), both between replicates within the same labelling plate and between all replicates in the same assay plate. Furthermore, the median CV between assay plates in the first run was 8% (IQR = 2) and the median CV between the first and the second run was 10% (IQR = 3). Spearman’s correlations were calculated (Additional file 7) to further investigate intra- and inter-assay variability. The Spearman’s Rho for intra-assay correlation was 0.999 and for the inter-assay correlation 0.965. Signals from the two positive loading controls (anti-albumin and anti-human IgG) were used to identify wells to be excluded from further analysis. A stringent quality control of the antibodies was performed, including a Spearman’s correlation between assays above 0.85. Inter-assay correlations are shown for each presented antibody (Additional files 8, 9, 10). Signals correlating with negative controls (rabbit IgG and bare bead) were also excluded from further analysis. After the quality control, the dataset contained protein profiles of 70 proteins, targetted by 99 antibodies. Among the presented antibodies targetting the same protein, 13 proteins had antibody correlations above 0.7 (Additional file 11).

## Results

### Protein profiles separate malaria cases from community controls

First, the relative protein levels were examined for all antibodies that passed the quality control to identify proteins with divergent amounts in community controls compared to malaria patients. Significant alterations in relative protein levels (p-values < 0.001) were observed for 76 antibodies corresponding to 57 proteins (Additional file 4).

Then, 27 proteins (number of UniProt IDs) were selected from this list with p-values below the arbitrary cut-off 10^−14^ for further analysis (Table 1). This stringent approach allowed us to focus on the proteins with the most significant differences. These 27 proteins included proteins with primary biological functions such as acute phase reactants of the innate immune response (e.g. CRP, LBP, TNF), proteins connected to cell migration (LCP1) or cell adhesion (VCAM1, VWF) and both cell migration and adhesion (CSF1, ITGAV, IGFBP1) as well as other inflammatory proteins. Two tissue remodelling proteins (CALCA, MMP2) and several proteins not traditionally involved in inflammation were also identified (e.g. ADSSL1, MYL3, NEFM, TIPIN). Three proteins displayed lower protein levels in the malaria cases compared to the controls; anti-adhesive SPARC (also known as Osteonectin), cell migration chemoattractant CCL5 (also known as RANTES) and apoptotic CTSD.

**Table 1:** Proteins present in divergent levels in community controls compared to malaria patients

Primary biological process	Gene name	Uniprot ID	Gene description	p-value	Antibody
Cell migration and adhesion	*CCL5*	*P13501*	*C*–*C motif chemokine ligand 5*	*1E−38*	*HPA010552*+
	CSF1	P09603	Colony stimulating factor 1	5E*−*60	HPA061864+
	IGFBP1	P08833	Insulin like growth factor binding protein 1	1E*−*21	MAB675 R&D
	ITGAV	P06756	Integrin subunit alpha V	3E*−*33	HPA004856
	LCP1	P13796	Lymphocyte cytosolic protein 1	6E*−*39	HPA019493+
	*SPARC*	*P09486*	*Secreted protein acidic and cysteine rich*	*5E−26*	*HPA003020*
	VCAM1	P19320	Vascular cell adhesion molecule 1	2E*−*74	HPA001618+
	VWF	P04275	von Willebrand factor	1E*−*75	HPA002082+
Acute phase response	CD14	P08571	CD14 molecule	6E*−*31	HPA001887+
	CEBPA	P49715	CCAAT/enhancer binding protein alpha	5E*−*83	HPA052734
	CRP	P02741	C-reactive protein	7E*−*109	HPA027396+
	LBP	P18428	Lipopolysaccharide binding protein	4E*−*87	HPA001508
	ORM1/ORM2	P02763, P19652	Orosomucoid 1/2	5E*−*27	HPA047725
	SERPINA3	P01011	Serpin family A member 3	2E*−*50	HPA002560+
	TNF	P01375	Tumor necrosis factor	3E*−*43	HPA077901+
Apoptosis	*CTSD*	*P07339*	*Cathepsin D*	*5E−15*	*HPA003001*
	RIPK2	O43353	Receptor interacting serine/threonine kinase 2	1E*−*14	HPA016499
Erythrocyte associated	EPB41L2	O43491	Erythrocyte membrane protein band 4.1 like 2	2E*−*15	HPA006642+
	GYPC	P04921	Glycophorin C (Gerbich blood group)	2E*−*28	HPA008965+
Tissue remodelling	CALCA	P01258	Calcitonin related polypeptide alpha	8E*−*28	HPA064453
	MMP2	P08253	Matrix metallopeptidase 2	2E*−*42	HPA001939
Axon associated	NEFM	P07197	Neurofilament medium	8E*−*16	HPA022845
DNA replication	TIPIN	Q9BVW5	TIMELESS interacting protein	4E*−*19	HPA039704
Motor protein	MYL3	P08590	Myosin light chain 3	3E*−*18	HPA016564
Purine biosynthesis	ADSSL1	Q8N142	Adenylosuccinate synthase like 1	4E*−*55	HPA052621
T-cell response	CD80	P33681	CD80 molecule	7E*−*24	HPA039851

Information about the 27 selected proteins (number of UniProt IDs) grouped on primary biological function with associated adjusted p-values based on t-test. Multiple proteins have previously been identified, by us and others, as having differential levels in malaria patients compared to controls and are marked [21, 32–39]. Proteins with lower levels in malaria cases compared to community controls are italicized. The proteins with support from multiple antibodies are marked + in the antibody column (see also Additional files 4, 11)

**Fig. 2:**
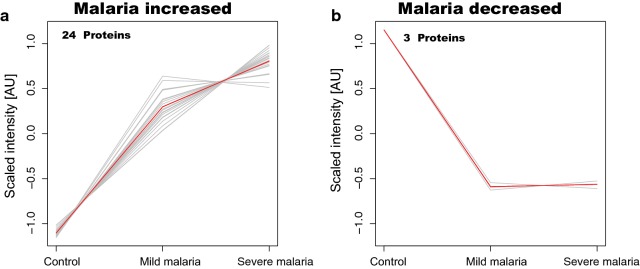
Cluster analysis of the 27 selected proteins with altered levels in plasma of patients with malaria compared to community controls. Self-Organizing Tree Algorithm (SOTA) analysis for the 27 selected proteins (p-values < 10^−14^) revealed two distinct trends in protein profiles showing the combined trend (red) and individual trends (grey). **a** Illustration of the 24 protein profiles with higher levels in the group of malaria cases compared to the group of controls. **b** Illustration of the three protein profiles with lower levels in the group of cases compared to the group of controls

The selected 27 proteins with the most significant differences were further characterized by SOTA clustering to reveal trends in protein level profiles with respect to disease progression (Fig. 2). From this clustering, 24 proteins showed a trend with higher levels in malaria patients compared to controls (Fig. 2a). In addition, an incremental trend in patients with severe disease compared to mild was observed. The remaining three proteins showed lower plasma levels in the malaria patient group compared to controls (Fig. 2b).

**Fig. 3:**
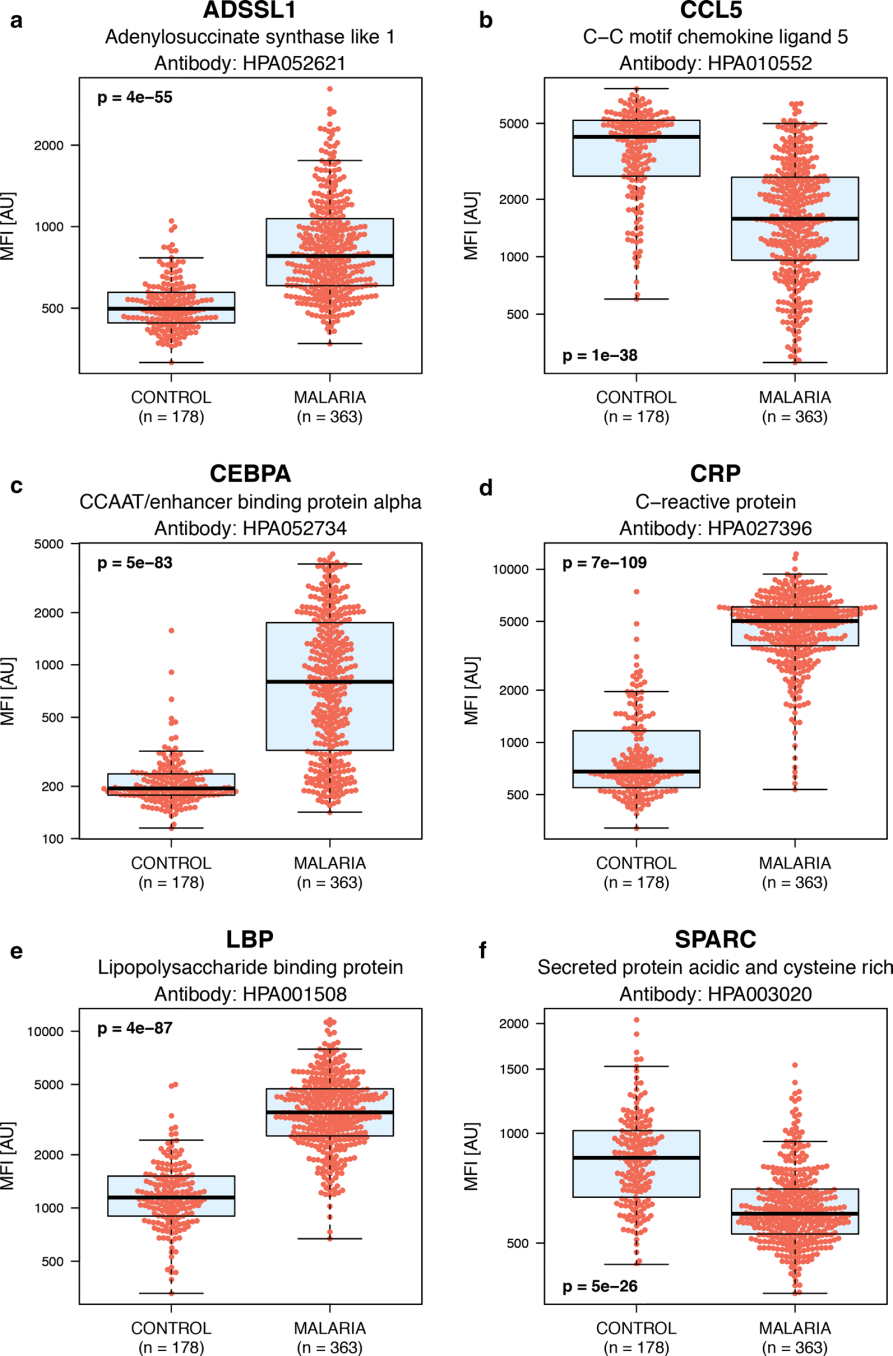
Boxplots outlining selected example proteins with differential protein levels in controls compared to malaria cases. Boxplots illustrating the relative intensities for six example proteins with differential protein levels in community controls compared to malaria cases (including both mild and severe malaria). Four of these proteins show increased levels in malaria cases compared to controls and the other two decreasing levels. These boxplots include the relative intensity signal for each individual sample (the individual red dots). **a** Adenylosuccinate synthase like 1 (ADSSL1). **b** C–C motif chemokine ligand 5 (CCL5). **c** CCAAT/enhancer binding protein alpha (CEBPA). **d** C-reactive protein (CRP). **e** Lipopolysaccharide binding protein (LBP). **f** Secreted protein acidic and cysteine rich (SPARC)

Furthermore, ROC curves were constructed for each respective protein, which together with significance levels further reveal the classification power of the selected proteins (Additional file 8). Six representative examples, showing both increased (ADSSL1, CEBPA, CRP, LBP) and decreased protein levels (CCL5, SPARC) in malaria patients compared to controls are shown in (Fig. 3).

**Fig. 4:**
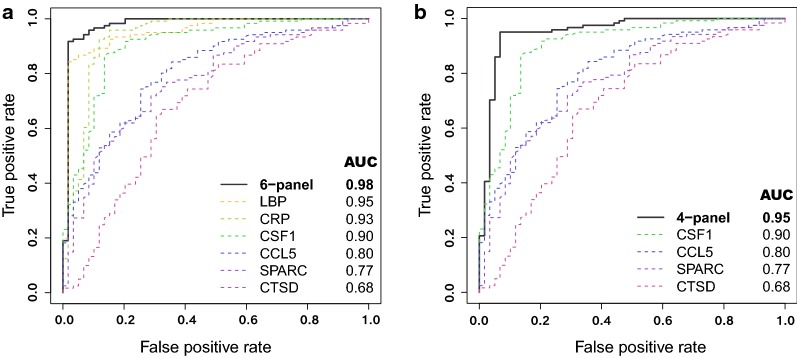
Protein panels with altered plasma levels between controls and malaria cases. Receiver operating characteristic (ROC) curves for the multi-protein panels and single protein ROC curves including values for the area under the ROC curve (AUC) for the antibodies selected from Lasso. **a** The 6-panel and single ROC curves for the six proteins: LBP (HPA001508), CRP (DYDY1707 R&D Systems), CSF1 (HPA061864), CCL5 (HPA010552), SPARC (HPA003020) and CTSD (HPA003001). **b** 4-panel multi-protein ROC curve excluding the classical inflammatory markers (CRP and LBP) and single protein ROC curves for CSF1, CCL5, SPARC and CTSD

Lasso regression analysis was used to identify whether combinations of proteins allowed an even higher discriminating power. Six proteins, covered by eight antibodies, were identified as the driving parameters that gave the optimal separation between the two sample groups in the training set. A multi-protein ROC curve based on logistic regression using all eight antibodies showed an AUC of 0.98 in the test set (Additional file 12). When including only one antibody per protein in the logistic regression model, this multi-protein 6-panel ROC curve still showed an AUC of 0.98 and the individual protein AUCs ranged between 0.68 and 0.95 (Fig. 4a). Out of the six proteins, three were identified as showing increased levels (CRP, CSF1, LBP) in malaria patients compared to controls while the remaining three displayed decreased levels (CCL5, CTSD, SPARC). Even when the classical inflammatory proteins CRP and LBP (also with the highest individual AUCs) were excluded, the joint AUC for the 4-panel was still 0.95 (Fig. 4b).

**Fig. 5:**
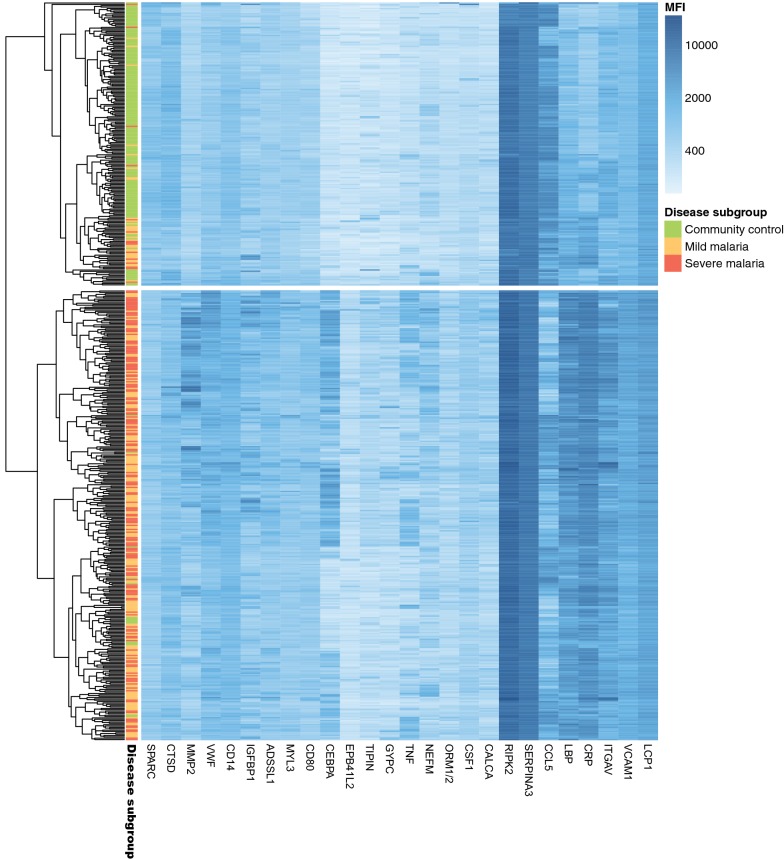
Heatmap of proteins discriminating between controls and malaria cases. Exploratory hierarchical clustering of all the 541 samples using the 27 proteins with adjusted p-values < 10^−14^ when discriminating between controls and malaria cases for all 541 samples. An extra sample-legend is provided for sample subgroup: community controls (green), mild malaria (orange) and severe malaria (red)

In addition, hierarchical clustering was used to identify potential sample clusters using all 541 samples and the 27 proteins (p-value < 10^−14^) (Fig. 5). Here, one cluster consisted of a majority of malaria cases (95% malaria cases and 5% controls) while the other consisted of a majority of control cases (78% controls and 22% malaria cases). Further scrutiny of the few malaria cases in the second cluster revealed the majority to be cases with mild disease.

### Protein profiles in mild and severe malaria cases

**Fig. 6:**
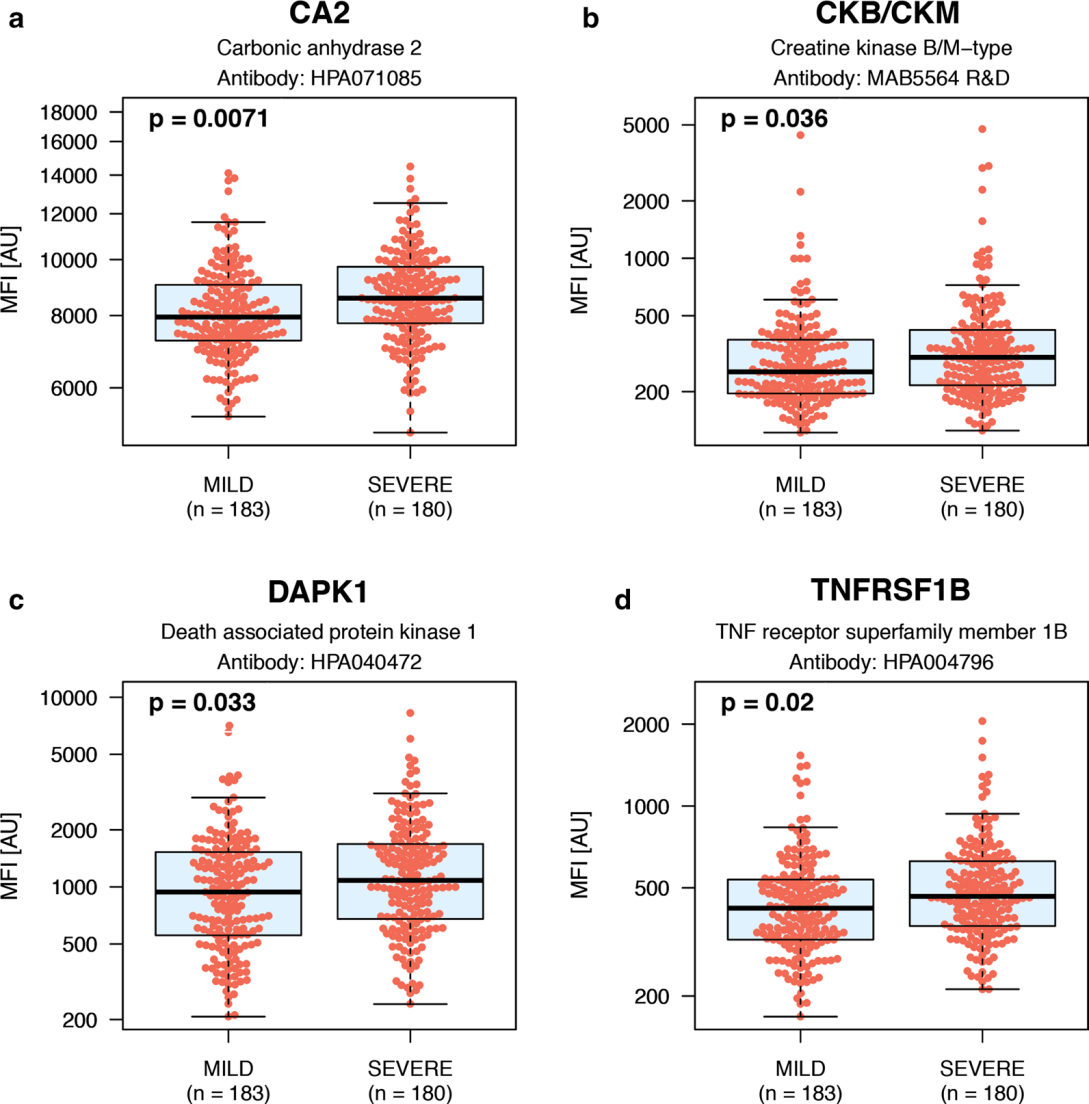
Boxplots outlining selected protein examples with differential levels in cases with severe malaria compared to mild malaria. Boxplots illustrating the relative intensities for four example proteins with differential protein levels in mild malaria compared to severe malaria cases. All four markers show increased levels of the protein in question for severe cases compared to mild malaria cases. These boxplots include the relative intensity signal for each individual sample (the individual red dots). **a** Carbonic anhydrase 2 (CA2). **b** Creatine kinase B/M-type (CKB/CKM). **c** Death associated protein kinase 1 (DAPK1). **d** Tumour necrosis factor receptor superfamily member 1B (TNFRSF1B)

Clinical manifestations of malaria infection are heterogeneous and cover a spectrum from mild disease to various degrees of severity. Less pronounced differences were found when comparing protein levels from patients with mild and severe malaria than between malaria cases and controls, which is to be expected. Hence, adjusted p-values < 0.05 were defined as significant for further analysis. Here, 37 proteins (number of UniProt IDs) were identified with the chosen significance level (Table 2 and Additional file 5). The identified proteins belonged to four major groups based on biological function: reactants of the acute phase response (e.g. CRP, LBP, TNF); erythrocyte specific (e.g. ANK1, BPGM, GYPC,); cell migration and adhesion (e.g. ADAMTS13, VWF); and tissue remodelling (e.g. AGT, CA2, MMP2) (Table 2). The relative differences for four representative proteins (Fig. 6) are displayed, out of the total set of 37 proteins (Additional file 9).

**Table 2:** Proteins present in divergent levels in mild compared to severe malaria cases

Primary biological process	Gene name	Uniprot ID	Gene description	p-value	Antibody
Cell migration and adhesion	ADAMTS13	Q76LX8	ADAM metallopeptidase with thrombospondin type 1 motif 13	2E−02	HPA042014
	CSF1	P09603	Colony stimulating factor 1	3E−02	HPA061864+
	IGFBP1	P08833	Insulin like growth factor binding protein 1	3E−02	MAB675 R&D
	ITGAV	P06756	Integrin subunit alpha V	2E−02	HPA004856
	VCAM1	P19320	Vascular cell adhesion molecule 1	2E−02	HPA069867+
	VWF	P04275	von Willebrand factor	4E−02	HPA002082
Erythrocyte associated	ANK1	P16157	Ankyrin 1	5E−02	HPA004842
	BPGM	P07738	Bisphosphoglycerate mutase	1E−02	HPA016493
	EPB41L2	O43491	Erythrocyte membrane protein band 4.1 like 2	3E−02	HPA005730
	GYPC	P04921	Glycophorin C (Gerbich blood group)	2E−02	HPA008965
	HBA1/2	P69905	Hemoglobin subunit alpha 1/2	1E−02	HPA043780
	MPP1	Q00013	Membrane palmitoylated protein 1	1E−02	HPA076675
Tissue remodelling	AGT	P01019	Angiotensinogen	3E−02	MAB3156 R&D
	CA2	P00918	Carbonic anhydrase 2	7E−03	HPA071085
	CALCA	P01258	Calcitonin related polypeptide alpha	4E−02	HPA064453
	ELANE	P08246	Neutrophil elastase	1E−02	MAB91671 R&D
	MMP2	P08253	Matrix metallopeptidase 2	2E−02	HPA001939
	MMP9	P14780	Matrix metallopeptidase 9	3E−02	HPA001238
Acute phase response	CD14	P08571	CD14 molecule	3E−02	HPA001887+
	CEBPA	P49715	CCAAT/enhancer binding protein alpha	3E−02	HPA052734
	CRP	P02741	C-reactive protein	3E−02	HPA027396
	LBP	P18428	Lipopolysaccharide binding protein	3E−02	HPA001508
	TNF	P01375	Tumour necrosis factor	5E−02	HPA077901+
Apoptosis	DAPK1	P53355	Death associated protein kinase 1	3E−02	HPA040472
	NGF	P01138	Nerve growth factor	5E−02	HPA063135
	RIPK2	O43353	Receptor interacting serine/threonine kinase 2	7E−03	HPA015764+
	TNFRSF1B	P20333	TNF receptor superfamily member 1B	2E−02	HPA004796
Axon associated	HAP1	P54257	Huntingtin associated protein 1	3E−02	HPA053019
	NEFM	P07197	Neurofilament medium	3E−02	HPA022845
Motor protein	MYL3	P08590	Myosin light chain 3	3E−02	HPA016564
	MYO15A	Q9UKN7	Myosin XVA	2E−02	HPA039770
T cell response	CD80	P33681	CD80 molecule	2E−02	HPA039851
	TNFSF13B	Q9Y275	TNF superfamily member 13b	4E−02	HPA030526
Cell cycle regulation	CDK14	O94921	Cyclin dependent kinase 14	3E−02	HPA015267
DNA replication	TIPIN	Q9BVW5	TIMELESS interacting protein	4E−02	HPA039704
Energy transduction	CKB/CKM	P12277, P06732	Creatine kinase B-type/M-type	4E−02	MAB5564 R&D

Information about the 37 proteins (number of UniProt IDs) showing differential levels between patients with severe and mild malaria. Proteins are grouped on primary biological function with displayed adjusted p-values based on the Wilcoxon rank-sum test. All these proteins showed higher levels in severe compared to mild malaria cases and include proteins, previously identified by us, as associated with severe malaria have been marked [21]. Proteins supported by multiple antibodies are marked + in the antibody column (see also Additional files 5, 11)

Dehydration is common among malaria patients and may present a potential experimental challenge as measuring relative protein levels may result in higher signals due to dehydration, regardless of the disease state. Therefore, clinically assessed hydration status of the patients were investigated to ensure that the increased protein levels in severe malaria cases compared to mild were not due to dehydration (which is more prominent among severe malaria patients). The samples were re-analysed, excluding dehydrated individuals in the two patient groups, to observe potential differences (Additional file 9). A total of 26 out of 37 proteins still showed a significant p-value (< 0.05) between the two sample groups.

Hierarchical clustering was used to visualize separation of mild and severe cases using these 37 proteins (Additional file 13). The two clusters revealed a minor separation between mild and severe disease, cluster one showing 44% of cases with mild malaria compared to 59% in cluster two. These findings highlight the complexity of the malaria disease pathogenesis.

**Fig. 7:**
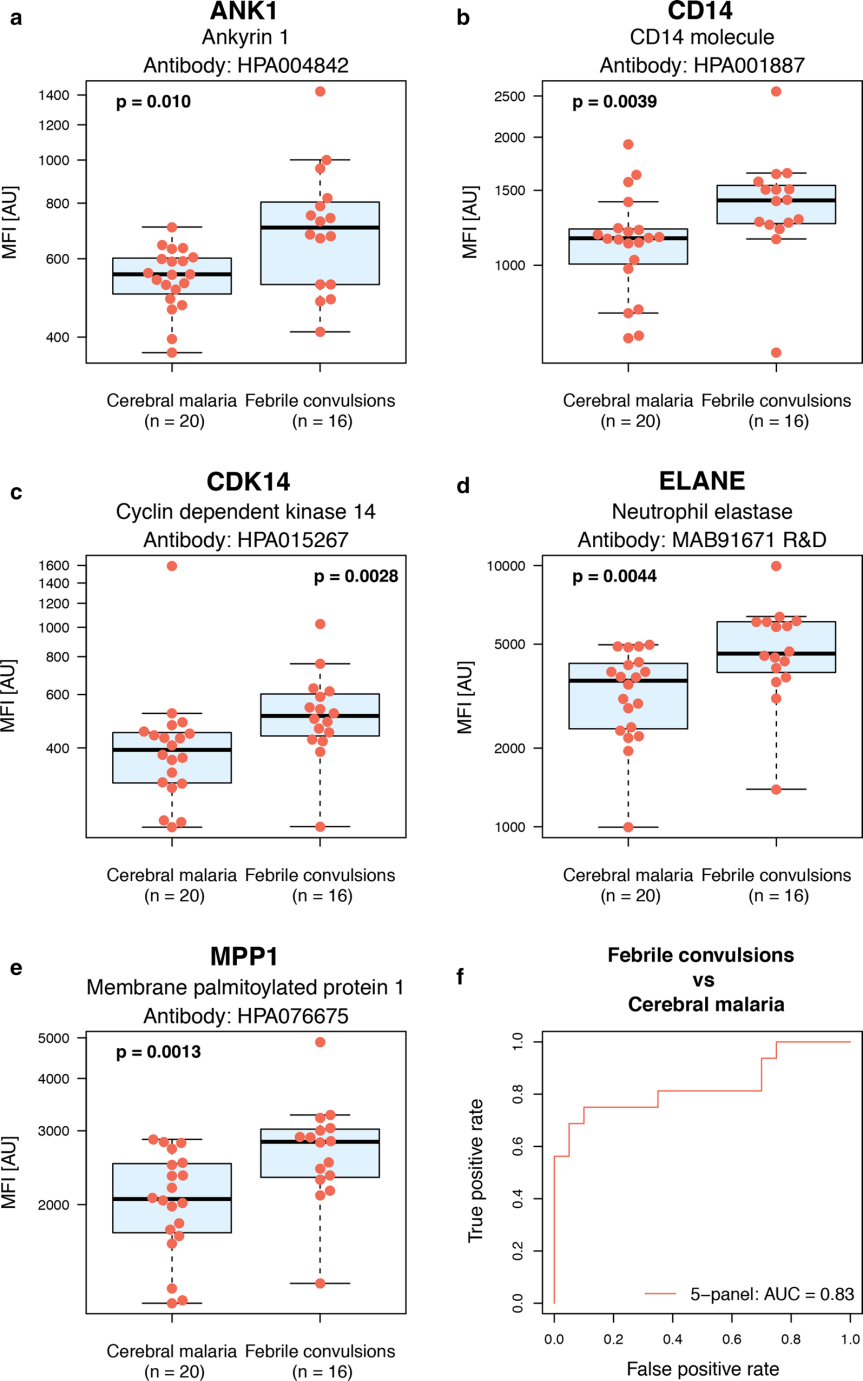
Boxplots outlining proteins with differential levels in cerebral malaria cases compared to febrile convulsion. Boxplots illustrating the relative intensities for the five proteins with differential protein levels in cerebral malaria compared to febrile convulsions during malaria infection (p-values < 0.01) together with a 5-protein multi-panel ROC curve. These boxplots include the relative intensity signal for each individual sample (the individual red dots). The ROC curve includes values for the area under the ROC curve (AUC) for the multi-protein panel. **a** Ankyrin 1 (ANK1). **b** Cyclin dependent kinase 14 (CDK14). **c** CD14 molecule (CD14). **d** Neutrophil elastase (ELANE). **e** Membrane palmitoylated protein 1 (MPP1). **f** 5-panel multi-protein signature of the five proteins

In an effort to elucidate differences between the severe manifestations, exploration of proteins with divergent levels between the discrete groups was carried out. Five proteins present at different levels in severe cases presented with either cerebral malaria of febrile convulsions were identified (Table 3 and Fig. 7). This was evident despite the clinical overlap of these different patient groups [40]. The five identified proteins (ANK1, CD14, CDK14, ELANE, MPP1) were found to be elevated in the febrile convulsion group compared to cerebral malaria. In addition, all five proteins also showed significant differences when comparing malaria patients to controls as well as mild malaria patients to severe. The proteins were combined into a multi-protein signature ROC curve (AUC of 0.83) in order to further explore the discriminatory power of these proteins (Fig. 7f).

**Table 3:** Proteins with differential levels observed between severe cases that presented with febrile convulsions compared to cerebral malaria

Primary biological process	Gene	Uniprot ID	Gene description	p-value	Antibody
Erythrocyte morphology	ANK1	P16157	Ankyrin 1	1e−02	HPA004842
	MPP1	Q00013	Membrane palmitoylated protein 1	1e−03	HPA076675
Acute phase response	CD14	P08571	CD14 molecule	4e−03	HPA001887+
Cell cycle regulation	CDK14	O94921	Cyclin dependent kinase 14	3e−03	HPA015267
ECM disassembly	ELANE	P08246	Neutrophil elastase	4e−03	MAB91671 R&D+

Information about the five proteins showing differential levels in patients with febrile convulsions compared to cerebral malaria, grouped on primary biological function, and unadjusted p-values based on Wilcoxon rank-sum test. All five proteins showed increased levels in patients with febrile convulsions compared to cerebral malaria. Proteins supported by multiple antibodies are marked + in the antibody column (see also Additional files 5, 12)

### Overlap and interaction networks between human proteins of variable levels during different stages of malaria infection

**Fig. 8:**
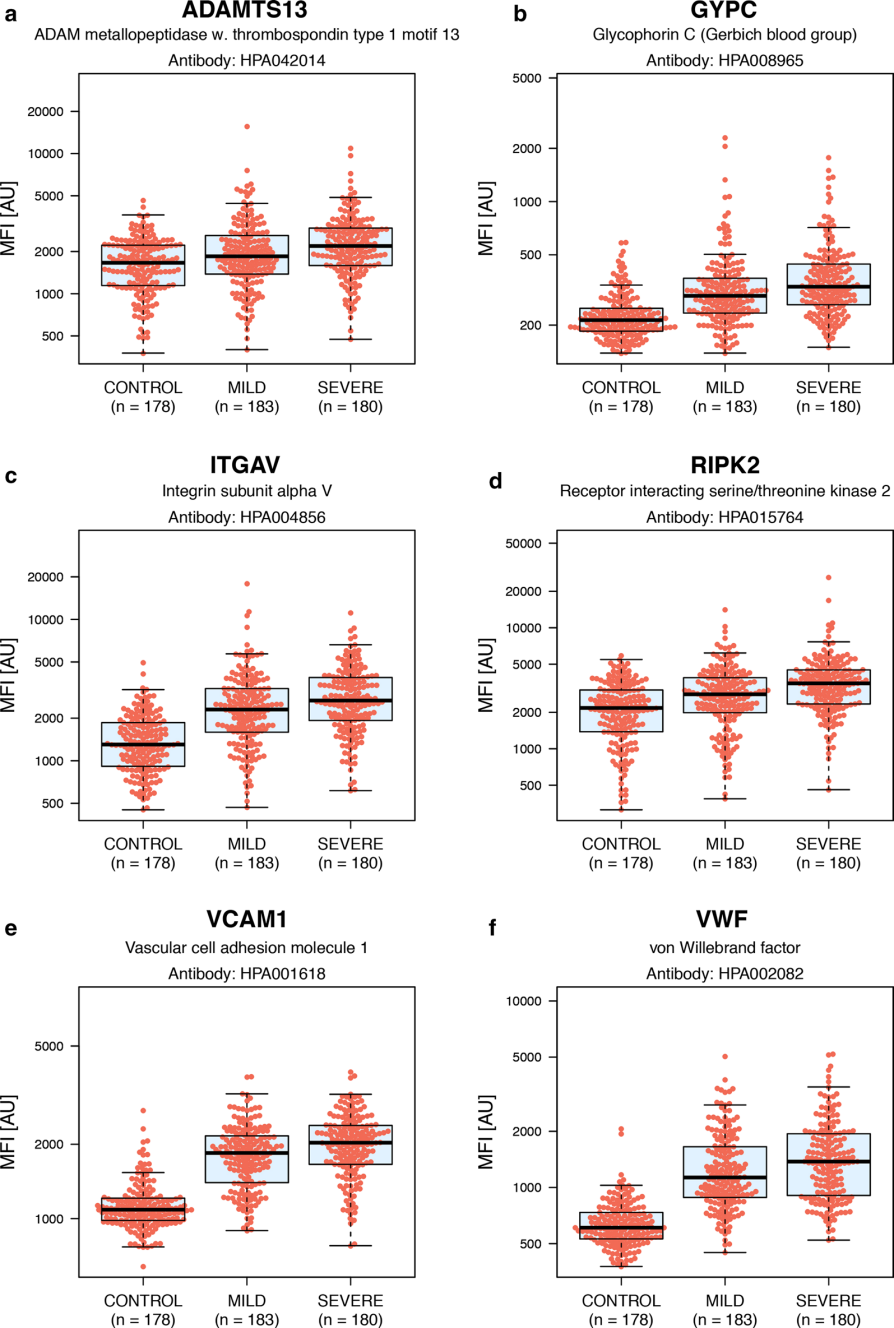
Boxplots outlining selected protein examples with altered levels in all the three disease subgroups. Boxplots illustrating the relative intensities for six proteins with differential protein levels between both controls and malaria patients, as well as between mild and severe malaria patients. All selected proteins display a rising-step trend in protein levels, increasing with disease severity. These boxplots include the relative intensity signal for each individual sample (the individual red dots). **a** ADAM metallopeptidase with thrombospondin type 1 motif 13 (ADAMTS13). **b** Glycophorin C (GYPC). **c** Integrin subunit alpha V (ITGAV). **d** Receptor interacting serine/threonine kinase 2 (RIPK2). **e** Vascular cell adhesion molecule 1 (VCAM1). **f** von Willebrand factor (VWF)

Within this study, a total of 61 proteins were identified by significant p-values in any of the comparisons of different sample groups. For the 57 proteins showing differential levels in malaria cases compared to controls, 24 proteins were noted as only associated with malaria infection compared to controls. The remaining 33 proteins had also significant differences in the comparison between mild and severe malaria patients. For these 33 proteins, a rising-step trend of increment with disease severity could be noted (Fig. 8). Four proteins (CA2, CKB, CKM, DAPK1) were only found to be associated with severe compared to mild malaria.

**Fig. 9:**
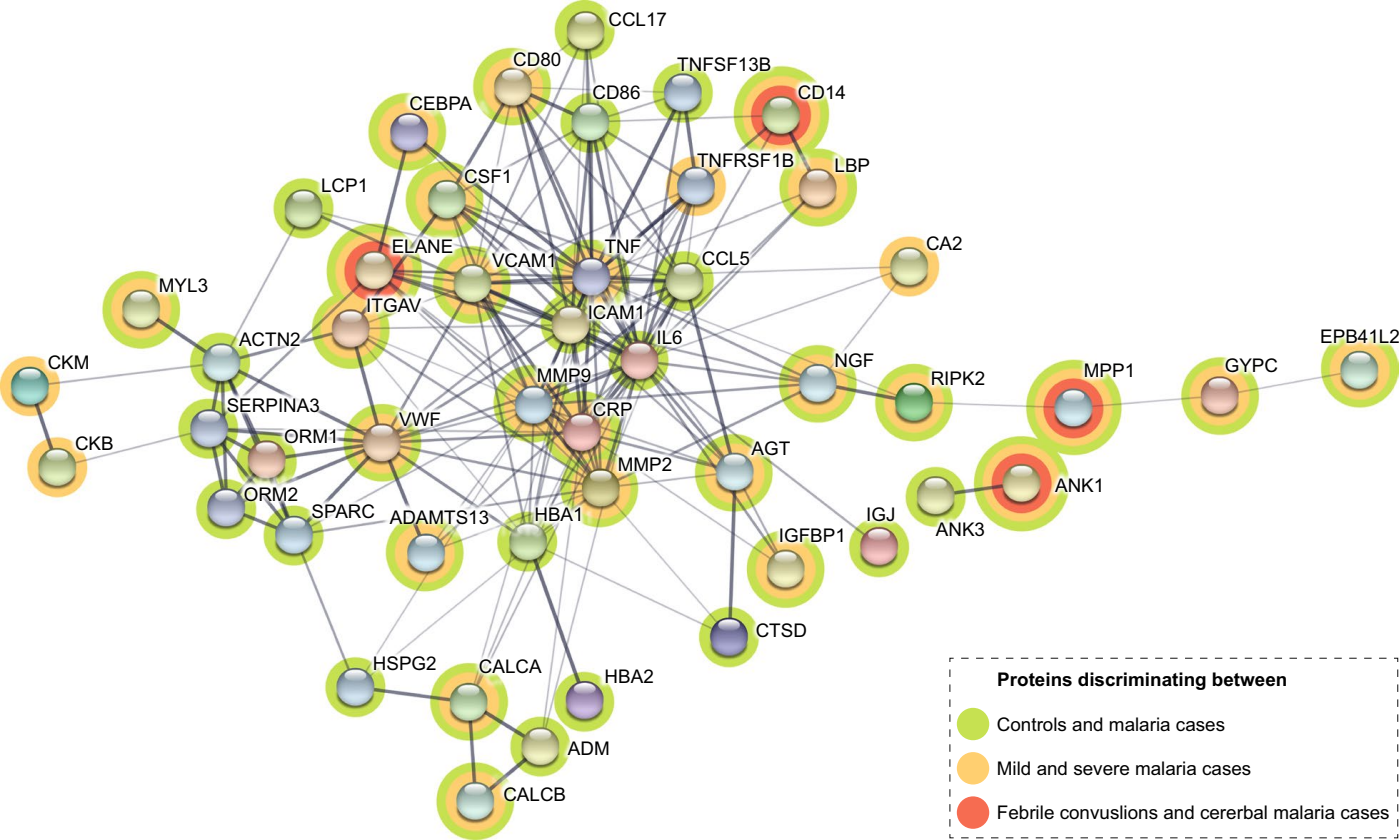
Protein–protein associations visualized with STRING. Here, displaying the 61 proteins with, significant p-values between any of the different group comparisons within this paper, but only showing connective nodes. The STRING network has been modified to show in which group comparison a specific protein had significantly different protein levels between the groups, illustrated by differently coloured backgrounds. The thickness of the lines indicates the strength of data support of that particular association

STRING network analysis was used to identify possible connections between all the 61 proteins found as significant between any of the presented groups (Fig. 9). Protein interaction network associations were identified for the majority of the 61 proteins, with TNF and VWF as two central nodes connecting acute phase inflammatory proteins, cell migration/adhesion and tissue remodelling proteins. In addition, the erythrocyte membrane proteins EPB41L2, GYPC and MPP1, as well as ANK1 and ANK3, were shown to be associated.

## Discussion

Herein is a comparative survey of proteins present at divergent levels in malaria patients at different clinical spectra of the disease and healthy community controls. This exploration aimed to increase the understanding of the host-parasite interaction and pathogenesis of the malaria disease, with a bearing towards the identification of putative prognostic protein markers. To accomplish this, an antibody-based suspension bead array approach with a targetted selection of antibodies towards human proteins was used. This selection included proteins previously identified as being of variable abundance during malaria infection, as well as a hypothesis-driven inclusion of antibodies towards proteins of unclear or unknown role in disease progression. Indeed, identification of a number of the proteins previously linked to malaria infection were identified. These proteins are involved in a range of biological processes including inflammatory response. The fact that these findings now have been replicated in several cohorts of patients, including the Rwandan cohort presented here, argues that the reliability and robustness of the proposed candidate markers are high. Moreover, proteins elevated in patients with malaria displayed a rising step trend with incremental levels as the disease progress to severe was observed. Thereby, a number of protein signatures were found that may shed light on the intricate interplay between the pathogen and the host.

Erythrocyte plasma membrane proteins were here identified as being elevated in malaria cases compared to controls and in severe malaria compared to mild malaria (GYPC) or increased in febrile convulsions compared to cerebral malaria (ANK1, MPP1). Interestingly, prior studies have found these three proteins (together with CA2) in microvesicles derived from parasitized erythrocytes [41]. To the best of our knowledge, there have been no reports of soluble forms of these three proteins in plasma reported to date. However, verification is needed to identify if the affinity proteomics findings are of soluble or vesicular origin. Further, two proteins of the immune response, that previously have been identified in different types of microvesicles: ITGAV [42] and CD80 were also found [43]. Previous studies have shown that certain parasite-derived molecules within host-derived extracellular microvesicles could be implicated in modulating leukocytic transcription and cytokine and chemokine responses and it has been suggested that these could be linked to parasite virulence and severe disease [44–48]. The identification of elevated levels of host proteins of possible microvesicular origin in patients with severe malaria supports these findings. If true, this suggests microvesicles from infected erythrocytes to be of very high relevance for the pathogenesis of the disease.

Cytoadherence is a central process in the biology of the *P. falciparum* malaria parasite as the adherence to the vascular endothelium protects the parasite from splenic clearance. The cytoadherence properties of infected erythrocytes are also intimately linked to severe malaria, as the resulting vascular occlusion prevents proper oxygenation of surrounding tissues and organs [49–52]. In line with previous findings [38], cytoadherence linked proteins VWF and ADAMTS13 were identified to vary in plasma abundance between malaria-infected and uninfected individuals. However, in previous findings by others, inhibition of ADAMTS13 has been reported while an increase in relative protein levels was observed here. This may not represent contradictory results, as relative protein levels and enzymatic activity are not necessarily interlinked.

Intriguingly, this study also identified proteins with a connection to cell adhesion and migration of substantially lower abundance in plasma of malaria patients compared to healthy controls. The decrease in CCL5 levels during malaria infection has been suggested to be a result of malaria-induced thrombocytopaenia [53]. Thrombocytes have also been identified to secrete SPARC [54], thus decreased levels of SPARC could also be the result of thrombocytopaenia. However, SPARC is also present in the extracellular matrix of a wide variety of other cells and tissues, including platelets, immune cells and endothelial cells, where abundance is particularly high in the latter. TNF is an inhibitor of SPARC which inhibits CEBPA [55] which could explain the opposite trends in protein profiles. Furthermore, VWF and SPARC have also been shown to share the same binding site of collagen [56], potentially competing for binding. The herein identified lowered abundance of SPARC could thus potentially aid the parasite in the process of sequestration as a reduction of anti-adhesive antigens could alter the adhesive potential of the host endothelial cells. However, the mechanistic modes by which plasma levels of SPARC could be reduced are many. There is a need to further investigate the possible anti-adhesive potential of SPARC during malaria infection experimentally.

The host proteins identified herein as variable upon malaria infection could serve purposes beyond providing clues to the understanding of host-parasite interaction and the pathogenesis of the disease. Although many of the targetted proteins are part of the pro- and anti-inflammatory responses that are shared with other infections, many others could potentially serve as prognostic markers for malaria infection. Microscopic examination of patient blood smears is still considered the golden standard of malaria diagnostics, but the specificity and sensitivity of this approach depend heavily on highly trained personnel [57, 58]. Antibody-based rapid diagnostic tests (RDTs) can be used with limited training and are often used as a complement to microscopy. However, recently it has been reported that one of the currently used RDTs suffers from both sensitivity and specificity issues [59–62]. Thus, complementing the existing diagnostic portfolio with new markers could be invaluable to ongoing efforts to eliminate and eradicate the disease. However, although evaluating the diagnostic potential of host protein markers would be attractive due to the possibility to prevent misdiagnosis due to natural selection of the targetted parasite antigens, this is a non-trivial task. Reference baseline levels of many proteins are highly individual [63, 64] and can affect the prognostic prediction power. Therefore, analysing longitudinal samples from patients with malaria infection would be needed to ensure prognostic prediction power as well as elucidate the timeframe needed for individual proteins to revert to baseline levels post infection. To further assure the usefulness of these candidate markers, disease controls from other illnesses such as bacterial and viral infections would also need to be investigated to reveal whether identified markers, by themselves or in combinations, are malaria-specific or not.

## Conclusions

Levels of 115 carefully selected human proteins were analysed in 541 paediatric plasma samples from community controls and patients with mild and various types of severe malaria, collected in Rwanda, by affinity proteomics. The results validate the previous findings of protein candidate markers associated with malaria infection done with the same affinity approach. New potential markers were identified that could be important leads towards an increased understanding of host-parasite interaction and pathogenesis of the disease. This study shows that a set of proteins, either individually or as a part of a panel, can display a highly significant discriminatory capacity between controls and malaria cases. Furthermore, a set of promising candidate markers were identified that significantly separate mild and severe malaria cases, despite the clinical overlap between these disease states. Consistent with the current literature, the identified increased levels of a variety of proteins related to acute phase immune responses and cytoadherence, but also new proteins that could be either linked to the same or other pathophysiological phenomena, such as infection-related microvesicular loads. Further evaluation and characterization of these presented proteins could enable increased understanding of their infection-biological roles and function. By increasing the understanding of the underlying mechanisms behind the clinical manifestations, this could potentially become an important component to improve treatment and prediction of disease progression.

## Additional files

**Additional file 1.** Clinical patient characteristics. Table summarizing the clinical characteristics for all the 541 included patients, divided by disease subcategory. Information about sex, age, nutrition status (based on WHO reference z-score), temperature and dehydration status are provided.

**Additional file 2.** Information about the number of individuals classified with the different severe stratifications. Table over the combination of dual severity stratifications for the 180 patients with severe malaria. Patients may suffer from one, two or more severe stratifications (not shown).

**Additional file 3.** Supplementary method information. **A**. Description of the sample labelling protocol including sample plate distribution and plate controls. **B**. Protocol for the antibody coupling onto the carboxylated beads and sample test for estimating coupling efficiency.

**Additional file 4.** Proteins significantly discriminating between community controls and malaria cases. Table with information about all antibodies targetting the 57 proteins (p-value < 0.001) discriminating between malaria cases and controls. The information provided is listed as follows: gene name, Uniprot ID, gene description, individual p-values and antibody ID. Antibodies which names include “HPA” are generated within the Human Protein Atlas project and “R&D” from R&D Systems.

**Additional file 5.** Proteins with significantly altered levels between mild malaria and severe malaria cases. Table with information about all antibodies targetting the 37 proteins (p-value < 0.05) with divergent levels in mild compared to severe malaria patients. The information provided is listed as follows: gene name, Uniprot ID, gene description, individual p-values and antibody ID. Names of antibodies from the Human Protein Atlas project are named “HPA” and from R&D Systems “R&D”.

**Additional file 6.** t-SNE plots. Plot A-F shows t-distributed stochastic neighbour embedding (t-SNE) plots to visualize the patient samples coloured based on different clinical parameters for all 61 proteins showing significant p-values between any of the investigated sample groups. All data points are shaped according to the sample group: community controls (point), mild malaria (rectangle) and severe malaria (triangle). **A**. Colouring by sample subgroup: community controls (green), mild malaria (orange) and severe malaria (red). **B**. Colouring by patient sex: F = female (blue), M = male (green). **C**. Colouring by patient age in months, ranging from 1–12 months (green) to 61–72 months (blue). **D**. Colouring by WHO reference z-score for nutrition based on patient age and weight. Normal nutrition (green), moderate undernutrition (blue), severe undernutrition (red). **E**. Colouring by patient body temperature at sampling, ranging from 35 °C (light yellow) to 41 °C (red). **F**. Colouring by signs of dehydration of the patient.

**Additional file 7.** Assay variation. Variation between assays calculated as Spearman’s Rho. **A**. Intra-assay variation. Each data point represents the calculated median antibody MFI for 88 replicated samples during the same assay run but in different assay plates. Spearman’s correlation was calculated to 0.999. **B**. Inter-assay variation. Each data point represents the calculated median antibody MFI for 325 replicated samples between two runs. Spearman’s correlation was calculated to 0.965.

**Additional file 8.** Information about proteins discriminating between controls and malaria patients. One panel for each of the proteins presented as potential markers for separating community controls from malaria cases with a p-value < 10^−14^. The panel includes one boxplot with protein levels for the group of controls and the malaria cases together with the Spearman’s correlation Rho for that protein between the two experiments (Exp 1 and Exp 2) and the classification power of the protein represented on ROC curves (including AUC values).

**Additional file 9.** Information about proteins with divergent levels between mild and severe malaria patients. One panel for each of the 37 proteins presented as potential markers for separating mild malaria from severe malaria cases with a p-value < 0.05. The panel includes one boxplot with protein levels for the two groups and p-values together with the Spearman’s correlation Rho for that protein between the two experiments (Exp 1 and Exp 2). The second boxplot illustrate the protein profile per sample group but only including the samples that did not show signs of dehydration.

**Additional file 10.** Information about proteins with divergent levels between febrile convulsion compared to cerebral malaria patients. One panel for each of the five proteins with a p-value < 0.01. Each panel includes a boxplot with protein levels for the group of patients with febrile convulsions and the group with cerebral malaria. The panel does also include the Spearman’s correlation Rho for that protein between the two experiments (Exp 1 and Exp 2) and the classification power of the protein represented on ROC curves (including AUC values).

**Additional file 11.** Spearman correlation’s for antibodies targetting the same protein. Table of presented antibodies targetting the same protein with Spearman’s Rho > 0.70. The table also includes the gene and gene description of the target protein of the antibodies.

**Additional file 12.** Multi-protein signature. Multi-protein ROC curve including all eight antibodies identified by Lasso and single antibody ROC curves with corresponding antibody ID and AUC.

**Additional file 13.** Heatmap over relative protein intensities between cases with mild and severe malaria. Exploratory hierarchical clustering of samples with mild and severe malaria for all proteins with p-values < 0.05 between these two groups. An extra sample-legend is provided for sample subgroup: mild malaria (orange) and severe malaria (red).
