# Supplementary material for: Levels of human proteins in plasma associated with acute paediatric malaria
Source: Malar J. 2018 Nov 15;17:426. doi: 10.1186/s12936-018-2576-y (PMC6238294; doi:10.1186/s12936-018-2576-y)
Supplement: Supplementary file 1 — Additional file 1. Clinical patient characteristics. Table summarizing the clinical characteristics for all the 541 included patients, divided by disease subcategory. Information about sex, age, nutrition status (based on WHO reference z-score), temperature and dehydration status are provided. [file 12936_2018_2576_MOESM1_ESM.pdf]

Additional file 1. Clinical patient characteristics

Variable	Parameter	Community controls	Mild malaria	Severe malaria
	**Total nr of samples**	**178**	**183**	**180**
**Sex [n, %]**	**Female**	97 (54.5%)	90 (49.2%)	95 (54.5%)
	**Male**	81 (45.5%)	93 (50.8%	85 (45.5%)
**Age [month]**	**Mean (± SD)**	41.3 ± 17.5	44.6 ± 18.1	46.0 ± 16.6
**Nutrition [n, %]**	**Moderate undernutrition**	11 (6.2%)	18 (9.8%)	22 (12.2%)
	**Normal nutrition**	128 (71.9%)	109 (59.6%)	110 (61.1%)
	**Severe undernutrition**	10 (5.6%)	5 (2.7%)	6 (3.3%)
	**Info is missing**	29 (16.3%)	51 (27.9%)	42 (23.3%)
**Temperature [°C]**	**Mean (± SD)**	36.6 ± 0.5	37.8 ± 1.1	38.3 ± 1.3
	**Info is missing [n, %]**	9 (5%)	11 (6%)	17 (9.4%)
**Dehydration [n, %]**	**No**	73 (41%)	150 (82%)	113 (62.8%)
	**Yes**	0 (0%)	18 (9.8%)	54 (30%)
	**Info is missing**	105 (59%)	15 (8.2%)	13 (7.2%)
