# Supplementary material for: Levels of human proteins in plasma associated with acute paediatric malaria
Source: Malar J. 2018 Nov 15;17:426. doi: 10.1186/s12936-018-2576-y (PMC6238294; doi:10.1186/s12936-018-2576-y)
Supplement: Supplementary file 2 — Additional file 2. Information about the number of individuals classified with the different severe stratifications. Table over the combination of dual severity stratifications for the 180 patients with severe malaria. Patients may suffer from one, two or more severe stratifications (not shown). [file 12936_2018_2576_MOESM2_ESM.pdf]

Additional file 2. Information about the number of individuals with the different forms of severe malaria

Hyper-parasitemia	Respiratory distress	Hyper-pyrexia	Prostration	Cerebral malaria	Severe anemia	Febrile convulsion	Only one symptom	
**70**	14	8	2	2	7	1	**47**	**Hyper-parasitemia**
	**53**	23	1	5	7	1	**14**	**Respiratory distress**
		**37**	0	1	2	0	**10**	**Hyper-pyrexia**
			**27**	0	1	0	**23**	**Prostration**
				**20**	1	0	**14**	**Cerebral malaria**
					**16**	0	**5**	**Severe anemia**
						**16**	**15**	**Febrile convulsion**
							**3**	**Only one symptom**
