# Supplementary material for: Levels of human proteins in plasma associated with acute paediatric malaria
Source: Malar J. 2018 Nov 15;17:426. doi: 10.1186/s12936-018-2576-y (PMC6238294; doi:10.1186/s12936-018-2576-y)
Supplement: Supplementary file 3 — Additional file 3. Supplementary method information. A. Description of the sample labelling protocol including sample plate distribution and plate controls. B. Protocol for the antibody coupling onto the carboxylated beads and sample test for estimating coupling efficiency. [file 12936_2018_2576_MOESM3_ESM.pdf]

Additional file 3. Supplementary method information

Additional file 3A. Method for sample labeling

The samples were directly labeled using biotin as described previously [23, 24]. In brief, 3 µL of plasma samples were diluted in 22 µL 0.1 M sterile filtered phosphate buffered saline (PBS) (09-9400, Medicago) in 96-well microtiter plates (732-4828, Thermo Scientific). Samples were allocated in a stratified distribution plate layout in regards to sample subgroup, patient sample age at sampling, sex and collection year. We used two types of plate controls; replicate plasma sample pools, to control for plate variability, and wells without sample. Subsequently, 5 µL NHS-PEG_4 biotin (21329, Thermo Scientific), dissolved in DMSO (276855, Sigma-Aldrich) and diluted in PBS, was added to the diluted plasma samples using a liquid handler (SELMA, CyBio). This corresponds to 20 ng NHS-biotin per well and a 10-fold theoretical excess of biotin over plasma protein. The samples were incubated for 2 hours at 4°C upon which the reaction was quenched by addition of a 250-fold molar excess of 0.5 M Tris-HCl pH=8 (T6066, Sigma-Aldrich) over biotin followed by a 20 minutes incubation at room temperature at 650 rpm. The biotin-labeled samples were stored at -20°C until further use.

Additional file 3B. Method for copuling of antibodies to beads

Antibodies were diluted to 16 µg/mL in 100 µL 0.1 M 2[N-Morpholino]ethanesulfonic acid (MES)-buffer (Sigma-Aldrich) pH=5 using a pipetting robot (TECAN EVO150) and coupled to carboxylated color-coded magnetic beads (MagPlex-C, Luminex Corp) as described previously [23, 24]. Beads (n = 500 000/ID), located in 96-well microtiter plates (781 101, Greiner BioOne), were washed with 80 µL 0.1 M NaH_2PO_4 (phosphate buffer) pH 6.2 (S3139, Sigma Life Science) with a plate washer/dispenser (EL406, Biotek) on a magnet before being suspended in 50 µL phosphate-buffer. Activation buffer, consisting of 10 mg/mL 1-ethyl-3-(3-dimethylaminopropyl) carbodiimide (EDC) (ProteoChem) and 10 mg/mL Sulfo-N-hydroxysulfosuccinimide (Sulfo-NHS) (24510, Thermo-Fisher Scientific) in phosphate buffer, was subsequently added to the beads, resulting in 0.5 mg EDC and 0.5 mg Sulfo-NHS per well. The beads suspended in activation buffer were incubated for 20 minutes at 650 rpm at room temperature and washed two times with 100 µL 0.1 M MES. The pre-diluted antibodies were added to the activated beads and incubated

for 2 hours at 650 rpm at room temperature. After incubation, the antibody-coupled beads were washed three times in 100 µL 1 × PBS, 0.05% Tween20 (BP337, Fisher Bioreagents) (PBS-T) and re-suspended in 50 µL storage buffer (Blocking Reagent for ELISA, Roche Applied Science). The individual bead IDs were pooled together after overnight blocking at 4°C, creating a bead stock containing all coupled bead IDs.

The coupling efficiency was tested using R-Phycoerythrin-conjugated (RPE) goat anti-rabbit IgG (111-116-144, Jackson), RPE-conjugated goat anti-mouse IgG (115-116-146, Jackson) and RPE-conjugated donkey anti-goat IgG (795-116-147, Jackson). 100 µL RPE-conjugated antibodies diluted to 0.5 µg/mL in PBS-T were added to 5 µL antibody-coupled bead stock in different wells, followed by incubation for 20 minutes at 650 rpm at room temperature. After incubation, the wells were washed three times with 100 µL PBS-T before analyzed on a Flexmap 3D instrument (Luminex corp.).
