# Supplementary material for: Levels of human proteins in plasma associated with acute paediatric malaria
Source: Malar J. 2018 Nov 15;17:426. doi: 10.1186/s12936-018-2576-y (PMC6238294; doi:10.1186/s12936-018-2576-y)
Supplement: Supplementary file 4 — Additional file 4. Proteins significantly discriminating between community controls and malaria cases. Table with information about all antibodies targetting the 57 proteins (p-value < 0.001) discriminating between malaria cases and controls. The information provided is listed as follows: gene name, Uniprot ID, gene description, individual p-values and antibody ID. Antibodies which names include “HPA” are generated within the Human Protein Atlas project and “R&D” from R&D Systems. [file 12936_2018_2576_MOESM4_ESM.pdf]

Additional file 4. Proteins significantly discriminating between community controls and malaria cases

Gene name	UniprotID	Gene description	p-value	Antibody
ACTN2	P35609	Actinin alpha 2	2E-09	HPA008315
ADAMTS13	Q76LX8	ADAM metallopeptidase w. thrombospondin type 1 motif 13	1E-07	HPA042014
ADM	P35318	Adrenomedullin	3E-10	HPA070396
ADSSL1	Q8N142	Adenylosuccinate synthase like 1	4E-55	HPA052621
AGT	P01019	Angiotensinogen	1E-13	MAB3156 R&D
ANK1	P16157	Ankyrin 1	2E-04	HPA004842
ANK3	Q12955	Ankyrin 3	2E-06	HPA055643
BPGM	P07738	Bisphosphoglycerate mutase	2E-10	HPA016493
CALCA	P01258;P06881	Calcitonin related polypeptide alpha	8E-28	HPA064453
CALCA/B	P10092;P01258;P06881	Calcitonin related polypeptide alpha/beta	3E-10	HPA043700
			5E-04	HPA059886
CCL17	Q92583	C-C motif chemokine ligand 17	4E-11	HPA068163
CCL5	P13501	C-C motif chemokine ligand 5	1E-38	HPA010552
			1E-04	HPA042290
CD14	P08571	CD14 molecule	6E-31	HPA001887
			1E-29	HPA002127
CD80	P33681	CD80 molecule	7E-24	HPA039851
CD86	P42081	CD86 molecule	3E-06	HPA072318
CDK14	O94921	Cyclin dependent kinase 14	3E-11	HPA015267
CEBPA	P49715	CCAAT/enhancer binding protein alpha	5E-83	HPA052734
CRP	P02741	C-reactive protein	7E-109	HPA027396
			4E-45	DY1707 R&D
CSF1	P09603	Colony stimulating factor 1	5E-60	HPA061864
			5E-51	HPA022244
			2E-34	HPA044339
CTSD	P07339	Cathepsin D	5E-15	HPA003001
ELANE	P08246	Neutrophil elastase	5E-13	MAB91671 R&D
			1E-04	HPA001184
EPB41L2	O43491	Erythrocyte membrane protein band 4.1 like 2	2E-15	HPA006642
			2E-10	HPA005730
ETFB	P38117	Electron transfer flavoprotein beta subunit	3E-07	HPA018910
GYPC	P04921	Glycophorin C (Gerbich blood group)	2E-28	HPA008965
HABP2	Q14520	Hyaluronan binding protein 2	4E-05	HPA019518
HAP1	P54257	Huntingtin associated protein 1	9E-06	HPA053019
			2E-04	HPA023368
HBA1/2	P69905	Hemoglobin subunit alpha 1/2	1E-05	HPA043780
HSPG2	P98160	Heparan sulfate proteoglycan 2	1E-04	HPA072690
ICAM1	P05362	Intercellular adhesion molecule 1	2E-08	DY720 R&D
IGFBP1	P08833	Insulin like growth factor binding protein 1	1E-21	MAB675 R&D
			4E-21	DY871 R&D
			4E-15	HPA046972
IL6	P05231	Interleukin 6	9E-13	HPA064428
ITGAV	P06756	Integrin subunit alpha V	3E-33	HPA004856
JCHAIN	P01591	Joining chain of multimeric IgA and IgM	3E-04	HPA044132
LBP	P18428	Lipopolysaccharide binding protein	4E-87	HPA001508
LCP1	P13796	Lymphocyte cytosolic protein 1	6E-39	HPA019493
LCP1, PLS1/3	P13796;Q14651;P13797	Lymphocyte cytosolic protein 1, Plastin 3/ Plastin 1,	3E-33	HPA000895
MMP2	P08253	Matrix metallopeptidase 2	2E-42	HPA001939
MMP9	P14780	Matrix metallopeptidase 9	1E-11	HPA001238
			5E-07	HPA063909
MPP1	Q00013	Membrane palmitoylated protein 1	9E-11	HPA076675
MYL3	P08590	Myosin light chain 3	3E-18	HPA016564
MYO15A	Q9UKN7	Myosin XVA	2E-10	HPA039770
NEFM	P07197	Neurofilament medium	8E-16	HPA022845
NGF	P01138	Nerve growth factor	4E-06	HPA063135
			7E-04	MAB256 R&D
ORM1/2	P02763;P19652	Orosomucoid 1/2	5E-27	HPA047725
PAFAH1B3	Q15102	Platelet activating factor acetylhydrolase 1b catalytic subunit 3	2E-04	HPA072475
RIPK2	O43353	Receptor interacting serine/threonine kinase 2	1E-14	HPA016499
			3E-11	HPA015764
SEC24C	P53992	SEC24 homolog C, COPII coat complex component	1E-07	HPA040213
SERPINA3	P01011	Serpin family A member 3	2E-50	HPA002560
			4E-50	HPA000893
SLC12A3	P55017	Solute carrier family 12 member 3	4E-09	HPA028748
SLC25A20	O43772	Solute carrier family 25 member 20	2E-04	HPA016862
SPARC	P09486	Secreted protein acidic and cysteine rich	5E-26	HPA003020
TIPIN	Q9BVW5	TIMELESS interacting protein	4E-19	HPA039704
TNF	P01375	Tumor necrosis factor	3E-43	HPA077901
			1E-13	HPA055037
TNFRSF1B	P20333	TNF receptor superfamily member 1B	7E-04	HPA004796
TNFSF13B	Q9Y275	TNF superfamily member 13b	6E-12	HPA030526
VCAM1	P19320	Vascular cell adhesion molecule 1	2E-74	HPA001618
			3E-39	HPA069867
			5E-29	HPA034795
VWF	P04275	von Willebrand factor	1E-75	HPA002082
			6E-38	HPA001815
