# Supplementary material for: Levels of human proteins in plasma associated with acute paediatric malaria
Source: Malar J. 2018 Nov 15;17:426. doi: 10.1186/s12936-018-2576-y (PMC6238294; doi:10.1186/s12936-018-2576-y)
Supplement: Supplementary file 5 — Additional file 5. Proteins with significantly altered levels between mild malaria and severe malaria cases. Table with information about all antibodies targetting the 37 proteins (p-value < 0.05) with divergent levels in mild compared to severe malaria patients. The information provided is listed as follows: gene name, Uniprot ID, gene description, individual p-values and antibody ID. Names of antibodies from the Human Protein Atlas project are named “HPA” and from R&D Systems “R&D”. [file 12936_2018_2576_MOESM5_ESM.pdf]

Additional file 5. Proteins with significantly altered levels between mild malaria and severe malaria cases

Gene	UniprotID	Gene description	p-value	Antibody
ADAMTS13	Q76LX8	ADAM metallopeptidase with thrombospondin type 1 motif 13	0.024	HPA042014
AGT	P01019	Angiotensinogen	0.029	MAB3156 R&D
ANK1	P16157	Ankyrin 1	0.046	HPA004842
BPGM	P07738	Bisphosphoglycerate mutase	0.012	HPA016493
CA2	P00918	Carbonic anhydrase 2	0.007	HPA071085
CALCA	P01258, P06881	Calcitonin related polypeptide alpha	0.043	HPA064453
CD14	P08571	CD14 molecule	0.029	HPA001887
			0.044	HPA002127
CD80	P33681	CD80 molecule	0.021	HPA039851
CDK14	O94921	Cyclin dependent kinase 14	0.026	HPA015267
CEBPA	P49715	CCAAT/enhancer binding protein alpha	0.029	HPA052734
CKB/CKM	P12277, P06732	Creatine kinase B/M-type	0.036	MAB5564 R&D
CRP	P02741	C-reactive protein	0.034	HPA027396
CSF1	P09603	Colony stimulating factor 1	0.029	HPA061864
			0.036	HPA044339
DAPK1	P53355	Death associated protein kinase 1	0.033	HPA040472
ELANE	P08246	Neutrophil elastase	0.012	MAB91671 R&D
EPB41L2	O43491	Erythrocyte membrane protein band 4.1 like 2	0.029	HPA005730
GYPC	P04921	Glycophorin C (Gerbich blood group)	0.016	HPA008965
HAP1	P54257	Huntingtin associated protein 1	0.026	HPA053019
HBA1/HBA2	P69905	Hemoglobin subunit alpha 1/2	0.012	HPA043780
IGFBP1	P08833	Insulin like growth factor binding protein 1	0.030	MAB675 R&D
ITGAV	P06756	Integrin subunit alpha V	0.024	HPA004856
LBP	P18428	Lipopolysaccharide binding protein	0.030	HPA001508
MMP2	P08253	Matrix metallopeptidase 2	0.023	HPA001939
MMP9	P14780	Matrix metallopeptidase 9	0.029	HPA001238
MPP1	Q00013	Membrane palmitoylated protein 1	0.012	HPA076675
MYL3	P08590	Myosin light chain 3	0.033	HPA016564
MYO15A	Q9UKN7	Myosin XVA	0.024	HPA039770
NEFM	P07197	Neurofilament medium	0.029	HPA022845
NGF	P01138	Nerve growth factor	0.047	HPA063135
RIPK2	O43353	Receptor interacting serine/threonine kinase 2	0.007	HPA015764
			0.015	HPA016499
TIPIN	Q9BVW5	TIMELESS interacting protein	0.038	HPA039704
TNF	P01375	Tumor necrosis factor	0.015	HPA055037
			0.032	HPA050631
			0.048	HPA077901
TNFRSF1B	P20333	Tumor necrosis factor receptor superfamily member 1B	0.020	HPA004796
TNFSF13B	Q9Y275	Tumor necrosis factor superfamily member 13b	0.036	HPA030526
VCAM1	P19320	Vascular cell adhesion molecule 1	0.012	HPA001618
			0.017	HPA069867
			0.026	HPA034795
VWF	P04275	von Willebrand factor	0.036	HPA002082
