# Supplementary material for: Levels of human proteins in plasma associated with acute paediatric malaria
Source: Malar J. 2018 Nov 15;17:426. doi: 10.1186/s12936-018-2576-y (PMC6238294; doi:10.1186/s12936-018-2576-y)

Additional file 6. t-SNE plots

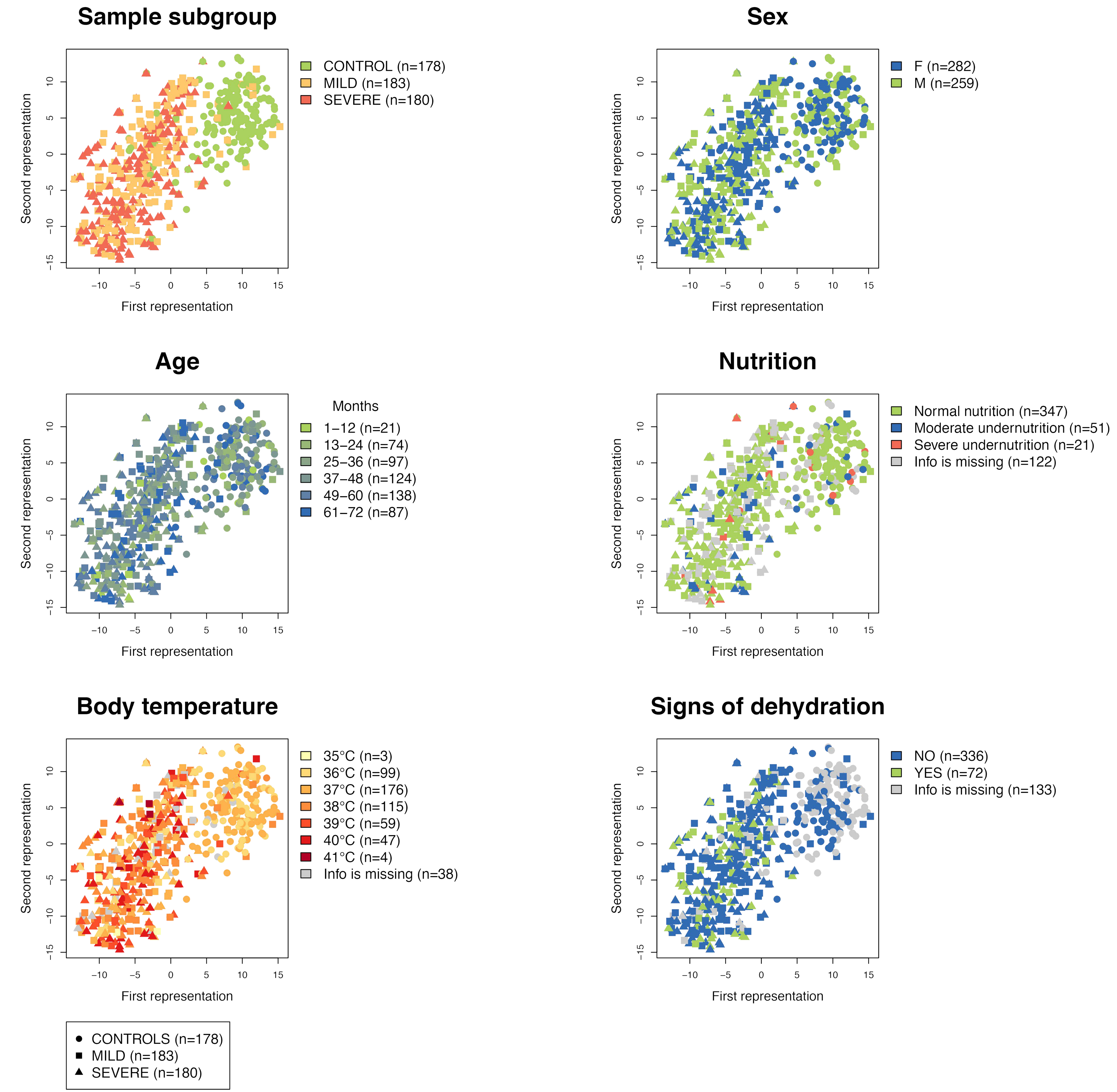

Supplement: Supplementary file 6 — Additional file 6. t-SNE plots. Plot A-F shows t-distributed stochastic neighbour embedding (t-SNE) plots to visualize the patient samples coloured based on different clinical parameters for all 61 proteins showing significant p-values between any of the investigated sample groups. All data points are shaped according to the sample group: community controls (point), mild malaria (rectangle) and severe malaria (triangle). A. Colouring by sample subgroup: community controls (green), mild malaria (orange) and severe malaria (red). B. Colouring by patient sex: F = female (blue), M = male (green). C. Colouring by patient age in months, ranging from 1–12 months (green) to 61–72 months (blue). D. Colouring by WHO reference z-score for nutrition based on patient age and weight. Normal nutrition (green), moderate undernutrition (blue), severe undernutrition (red). E. Colouring by patient body temperature at sampling, ranging from 35 °C (light yellow) to 41 °C (red). F. Colouring by signs of dehydration of the patient. [file 12936_2018_2576_MOESM6_ESM.pdf]
