# Supplementary figures and images for: Levels of human proteins in plasma associated with acute paediatric malaria
Source: Malar J. 2018 Nov 15;17:426. doi: 10.1186/s12936-018-2576-y (PMC6238294; doi:10.1186/s12936-018-2576-y)

# Additional file 7. Assay variation

**A** Intra assay variation

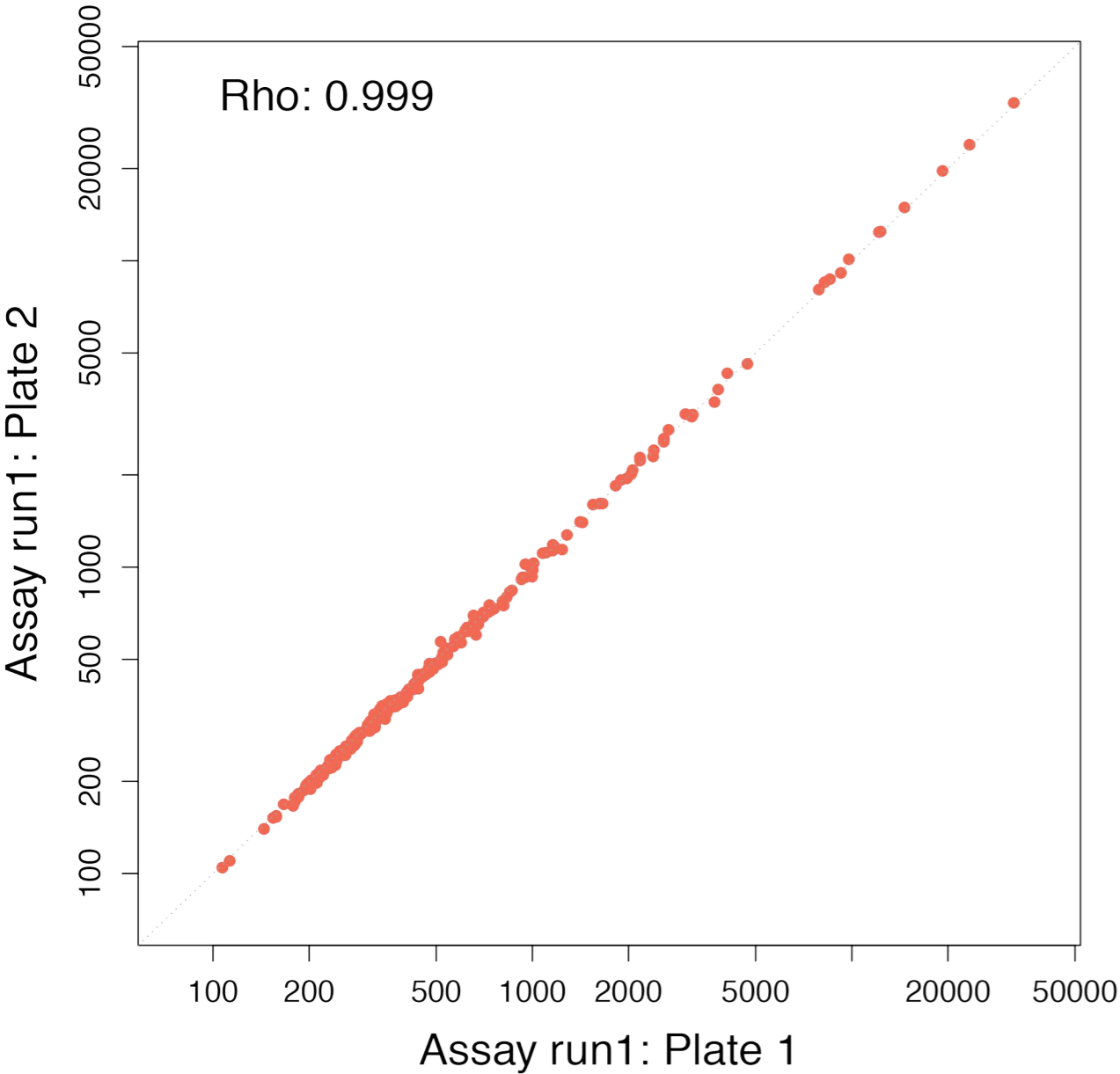

**B** Inter assay variation

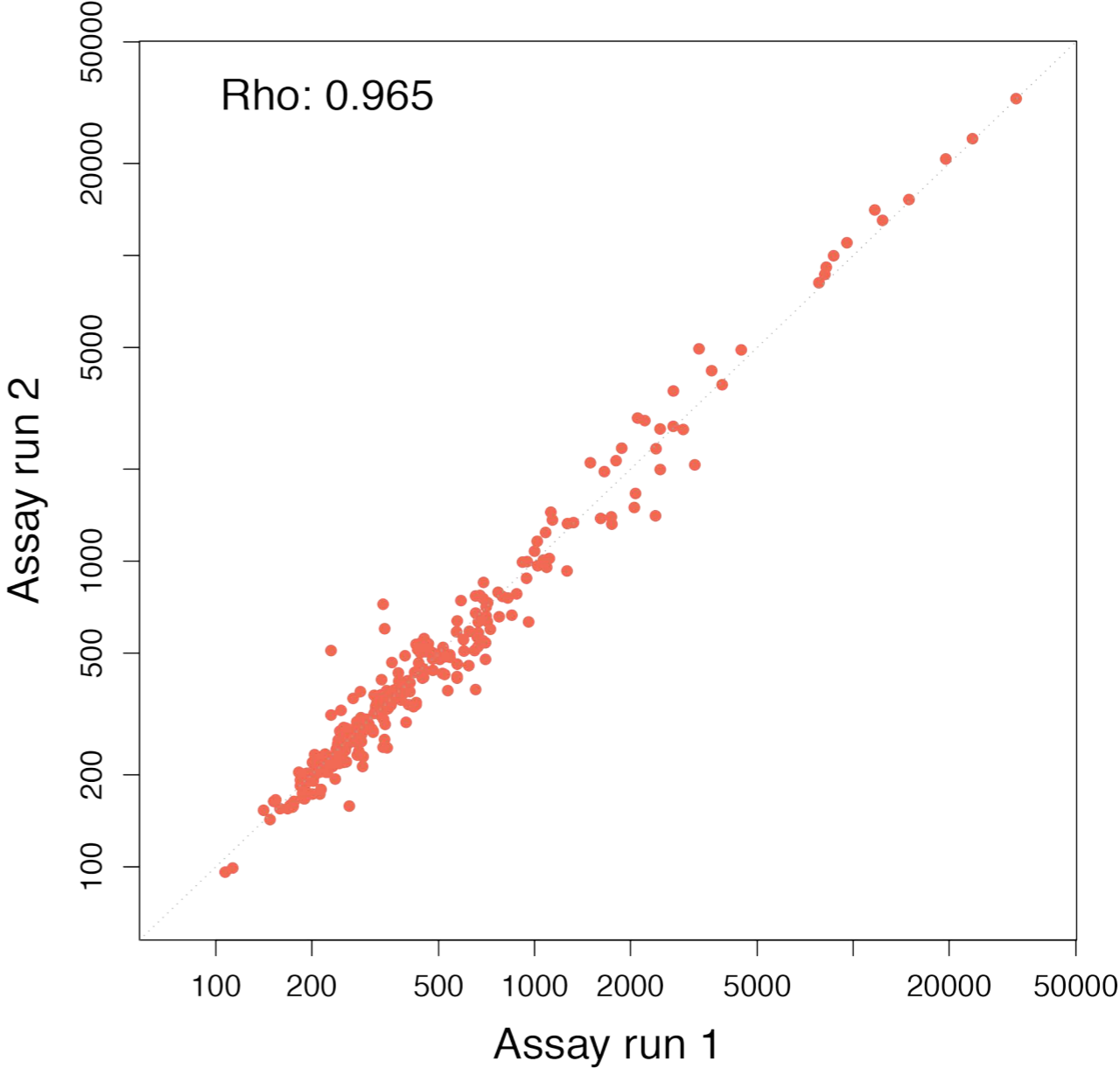

Supplement: Supplementary file 7 — Additional file 7. Assay variation. Variation between assays calculated as Spearman’s Rho. A. Intra-assay variation. Each data point represents the calculated median antibody MFI for 88 replicated samples during the same assay run but in different assay plates. Spearman’s correlation was calculated to 0.999. B. Inter-assay variation. Each data point represents the calculated median antibody MFI for 325 replicated samples between two runs. Spearman’s correlation was calculated to 0.965. [file 12936_2018_2576_MOESM7_ESM.pdf]

## Additional file 12. Multi-protein signature

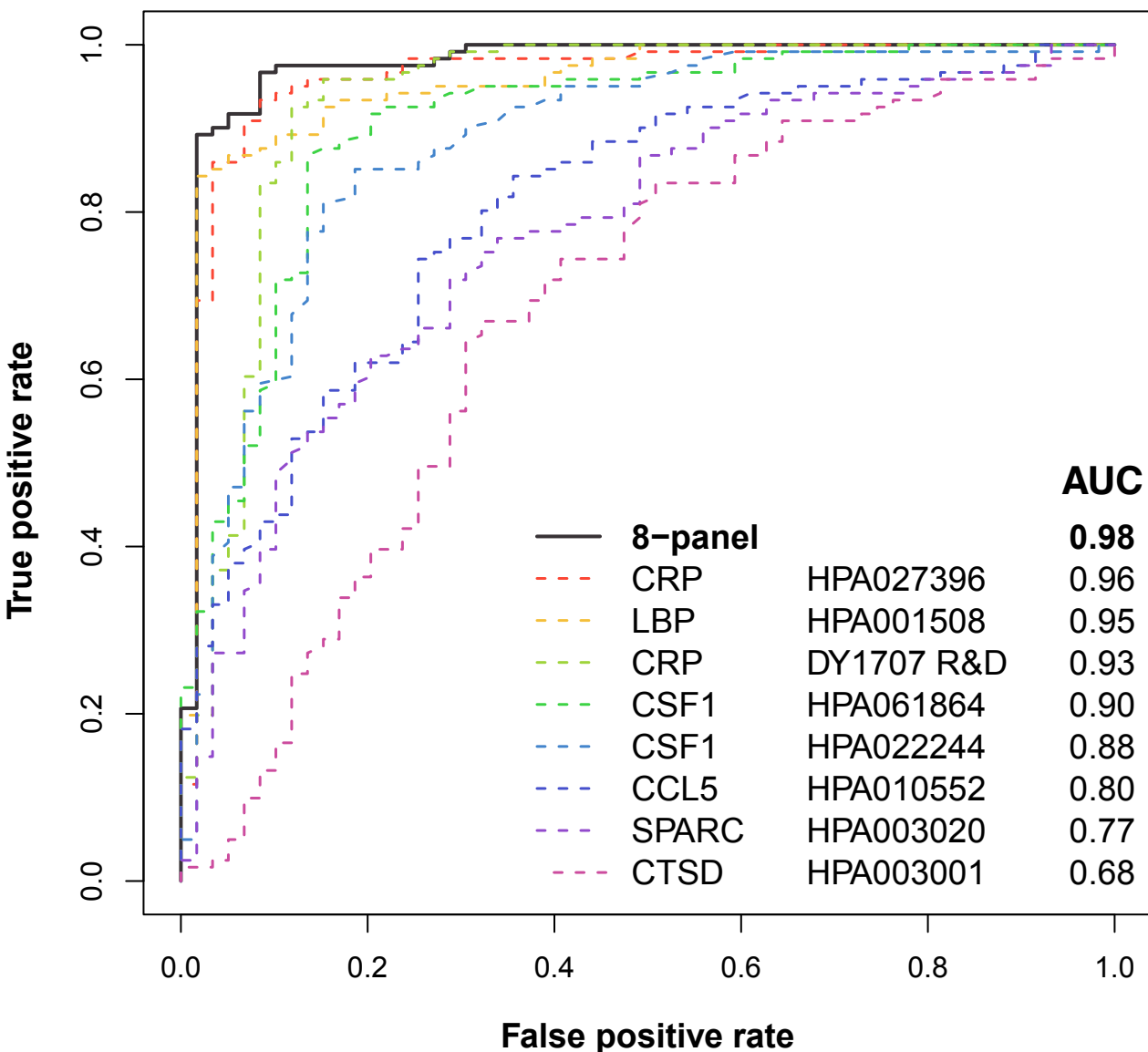

Supplement: Supplementary file 12 — Additional file 12. Multi-protein signature. Multi-protein ROC curve including all eight antibodies identified by Lasso and single antibody ROC curves with corresponding antibody ID and AUC. [file 12936_2018_2576_MOESM12_ESM.pdf]
