# Supplementary material for: Levels of human proteins in plasma associated with acute paediatric malaria
Source: Malar J. 2018 Nov 15;17:426. doi: 10.1186/s12936-018-2576-y (PMC6238294; doi:10.1186/s12936-018-2576-y)

Additional file 8. Information about proteins discriminating between controls and malaria patients

ADSSL1

Adenylosuccinate synthase like 1
Antibody: HPA052621

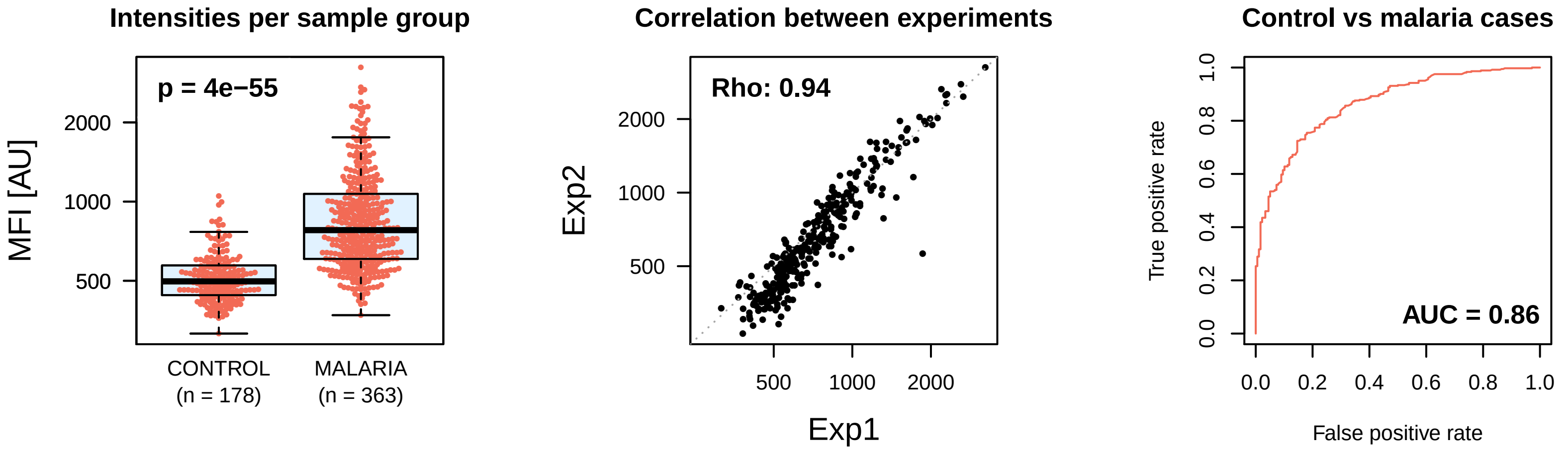

CALCA

Calcitonin related polypeptide alpha
Antibody: HPA064453

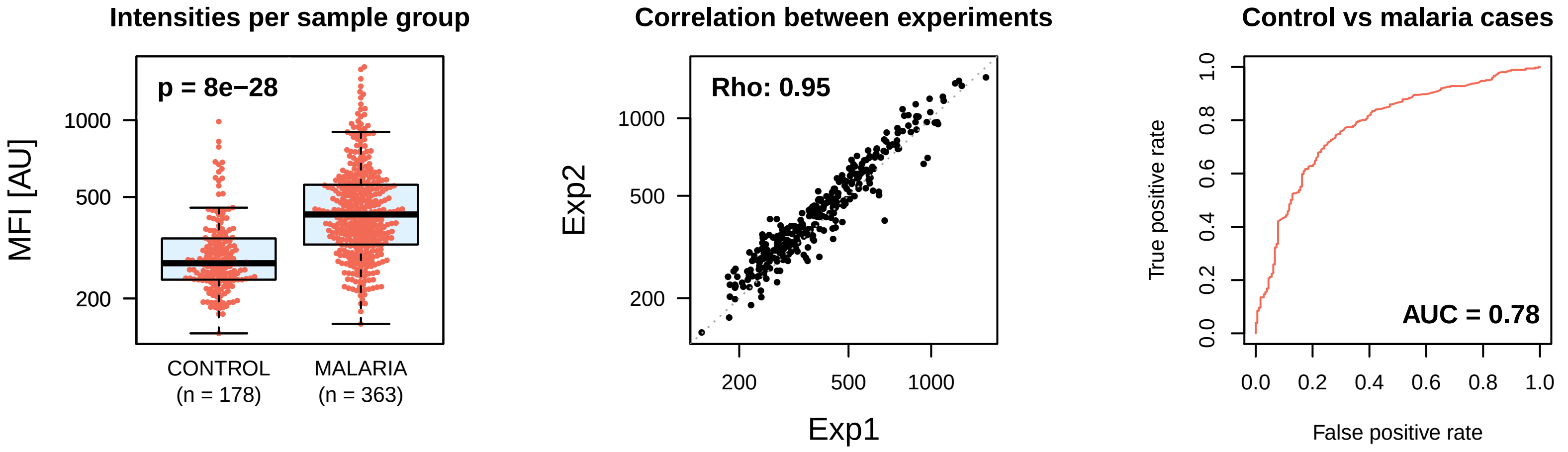

CCL5

C−C motif chemokine ligand 5
Antibody: HPA010552

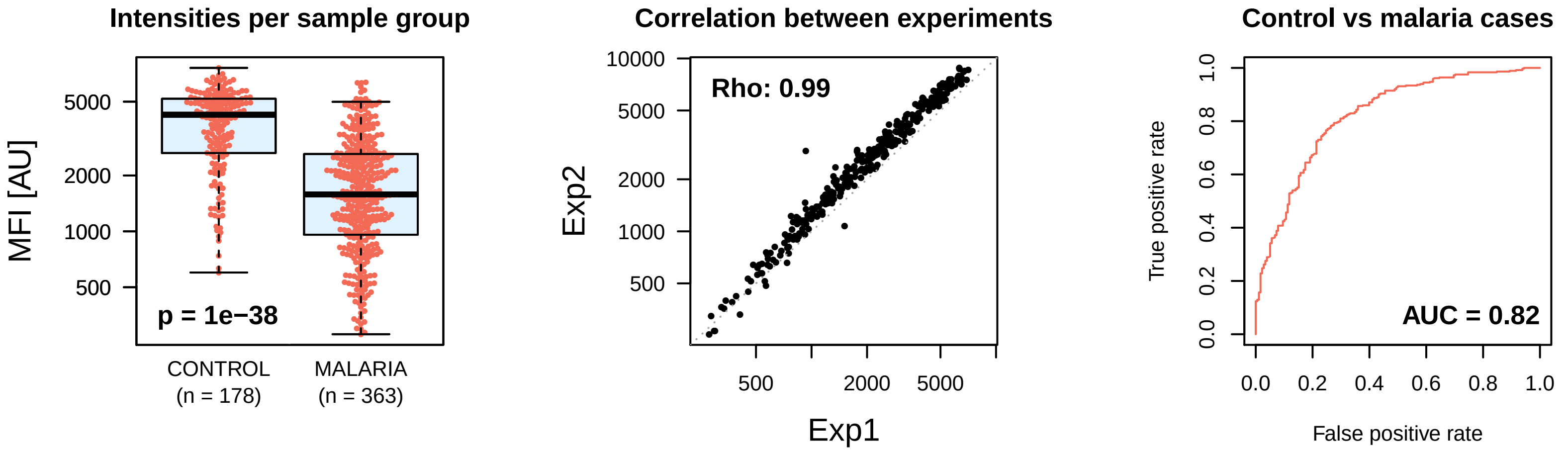

CD14

CD14 molecule
Antibody: HPA001887

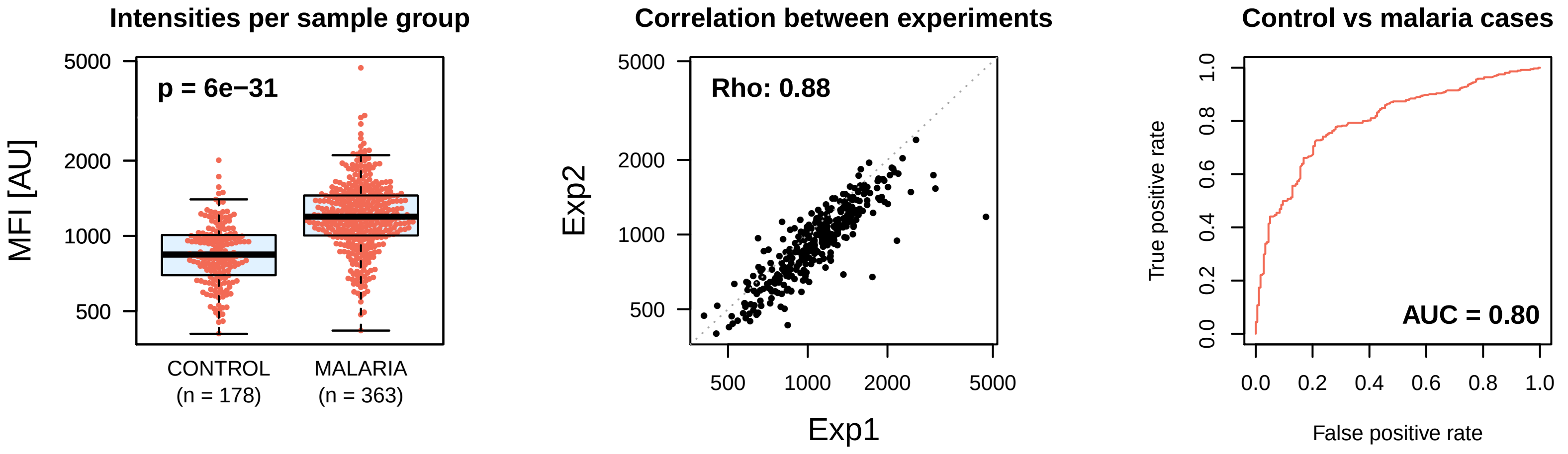

CD80

CD80 molecule
Antibody: HPA039851

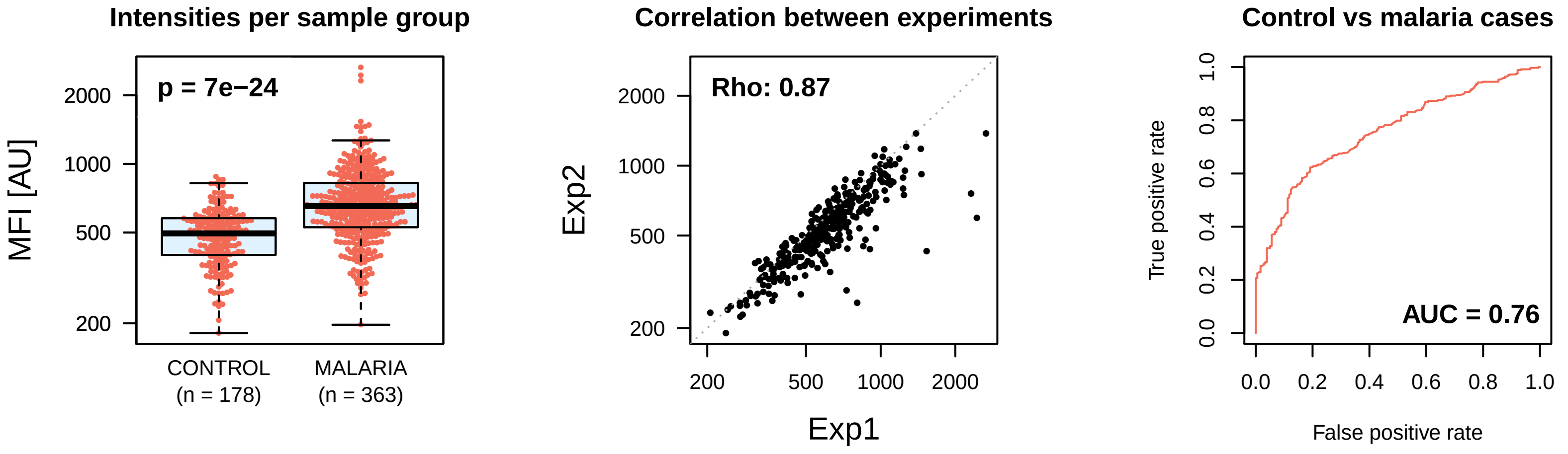

CEBPA

CCAAT/enhancer binding protein alpha
Antibody: HPA052734

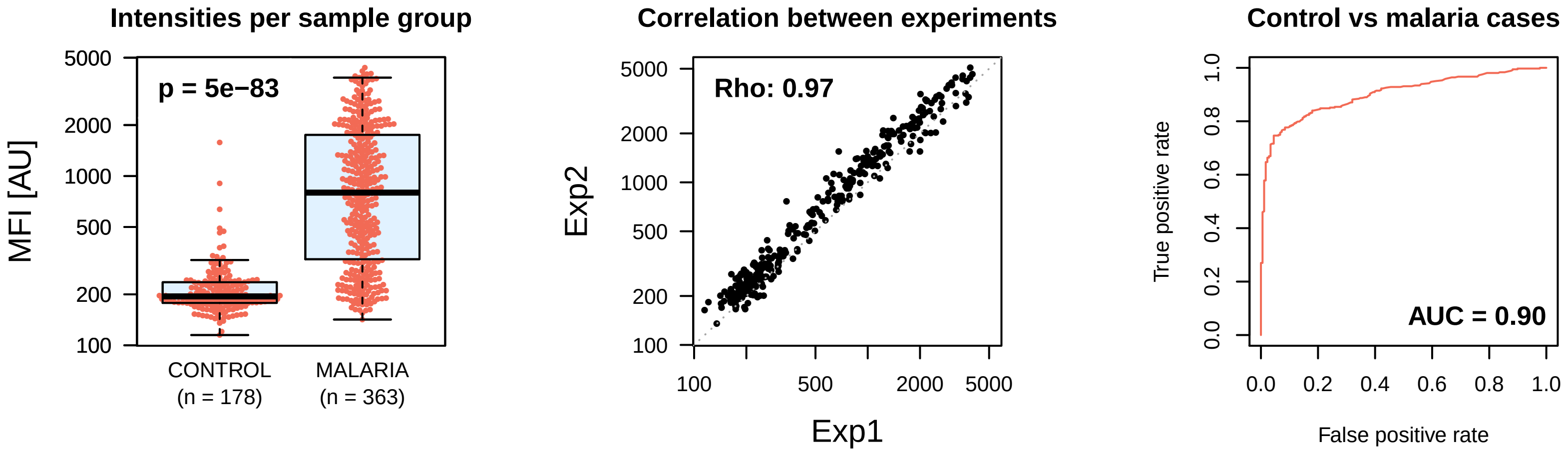

CRP

C−reactive protein
Antibody: HPA027396

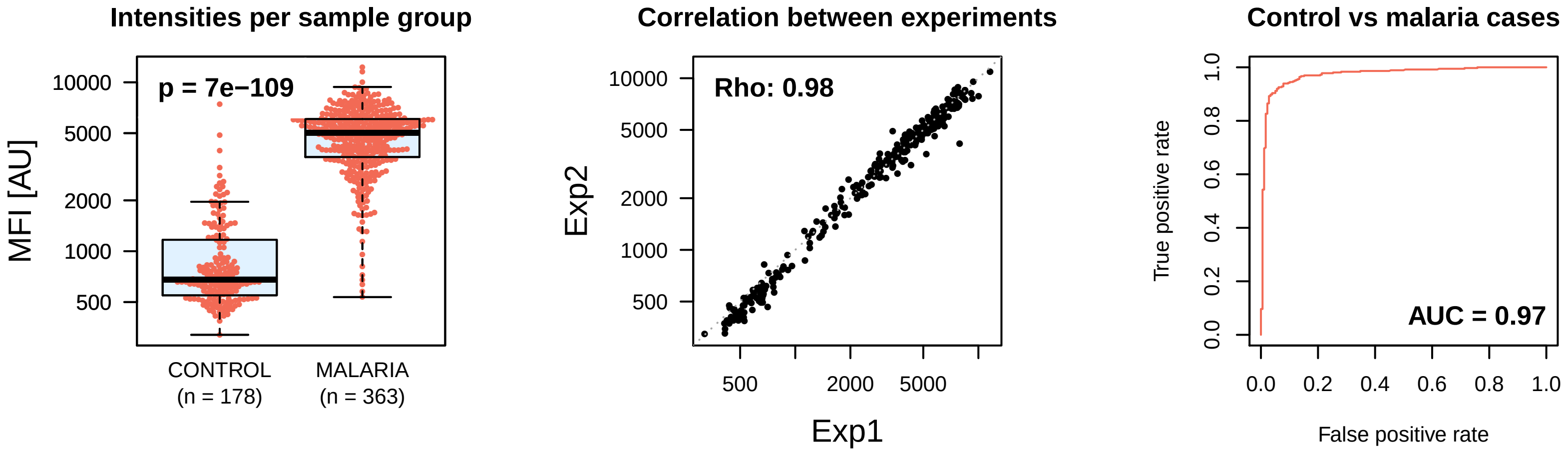

CSF1

Colony stimulating factor 1
Antibody: HPA061864

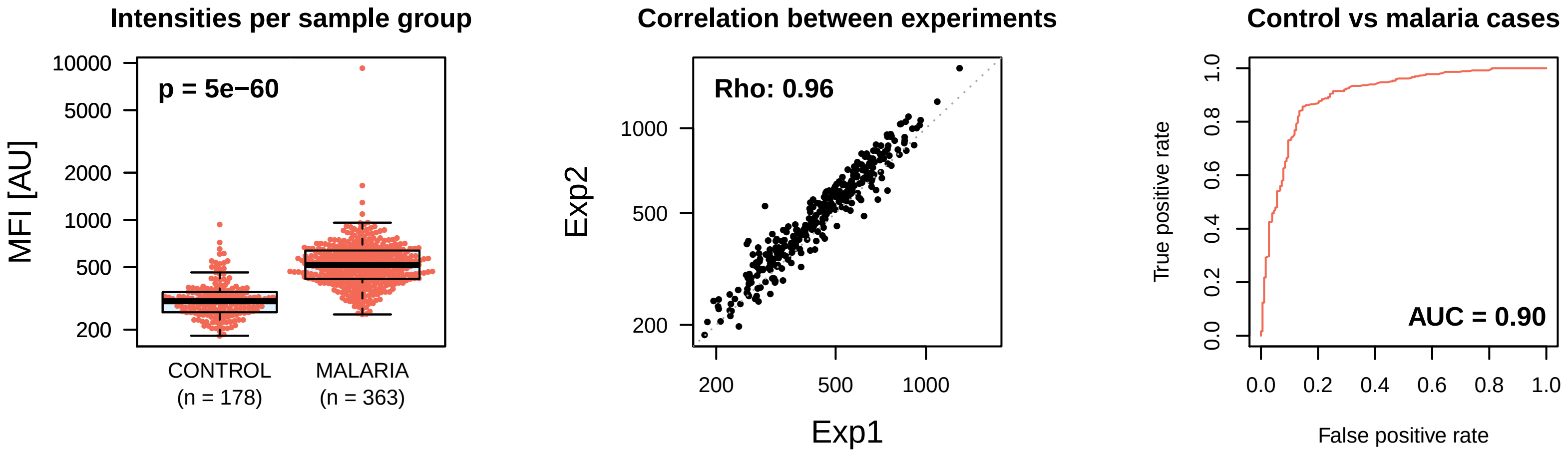

CTSD

Cathepsin D
Antibody: HPA003001

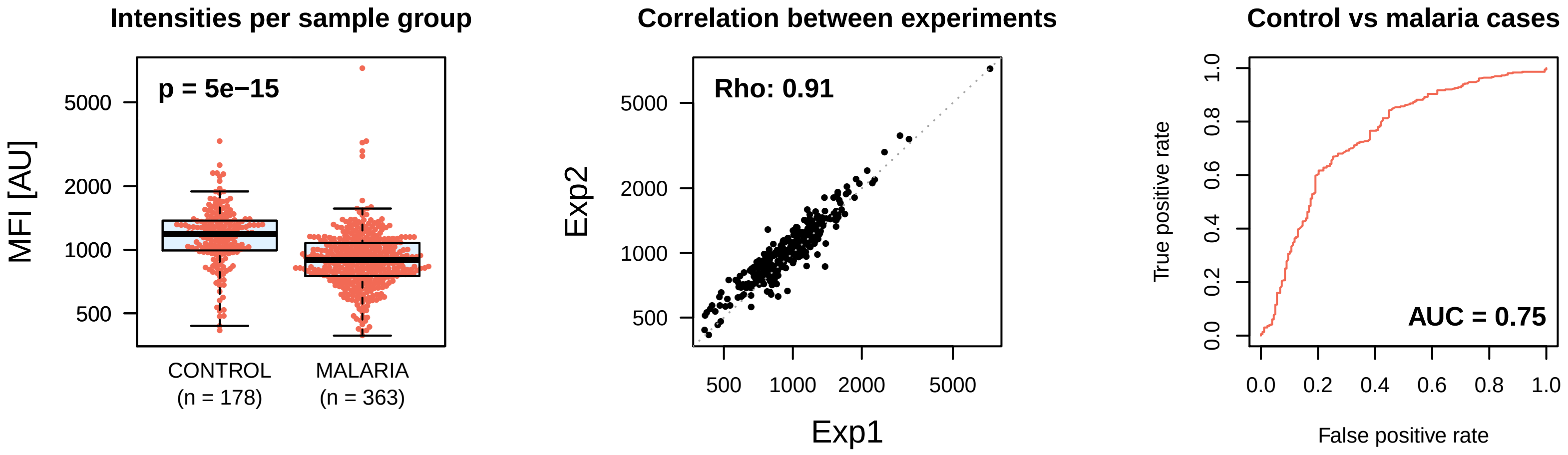

EPB41L2

Erythrocyte membrane protein band 4.1 like 2
Antibody: HPA006642

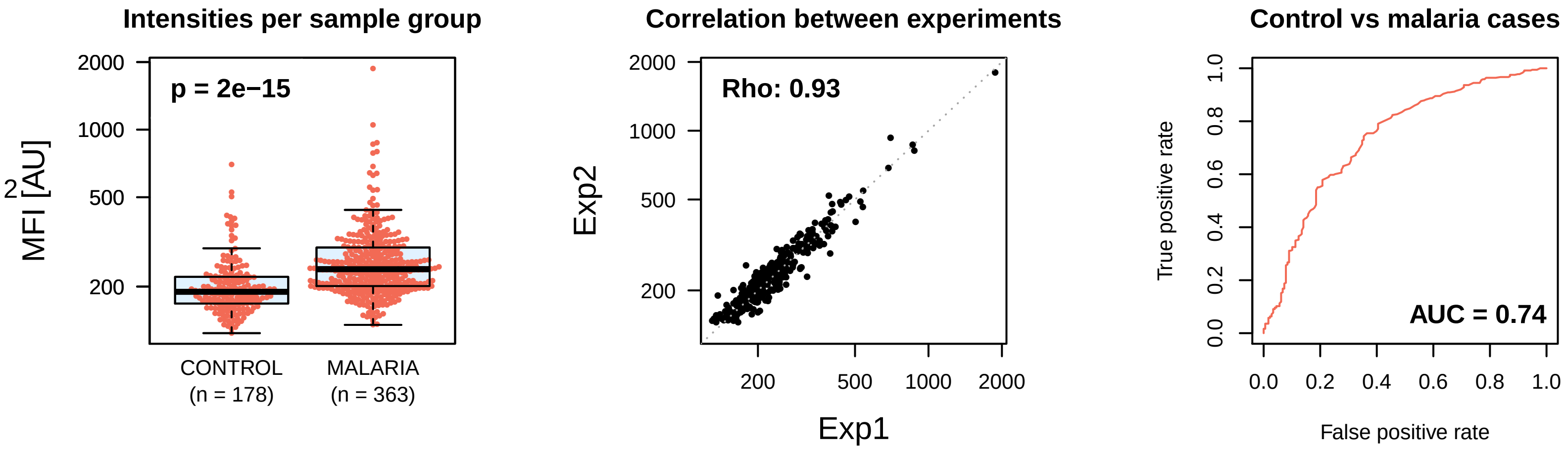

GYPC

Glycophorin C (Gerbich blood group)
Antibody: HPA008965

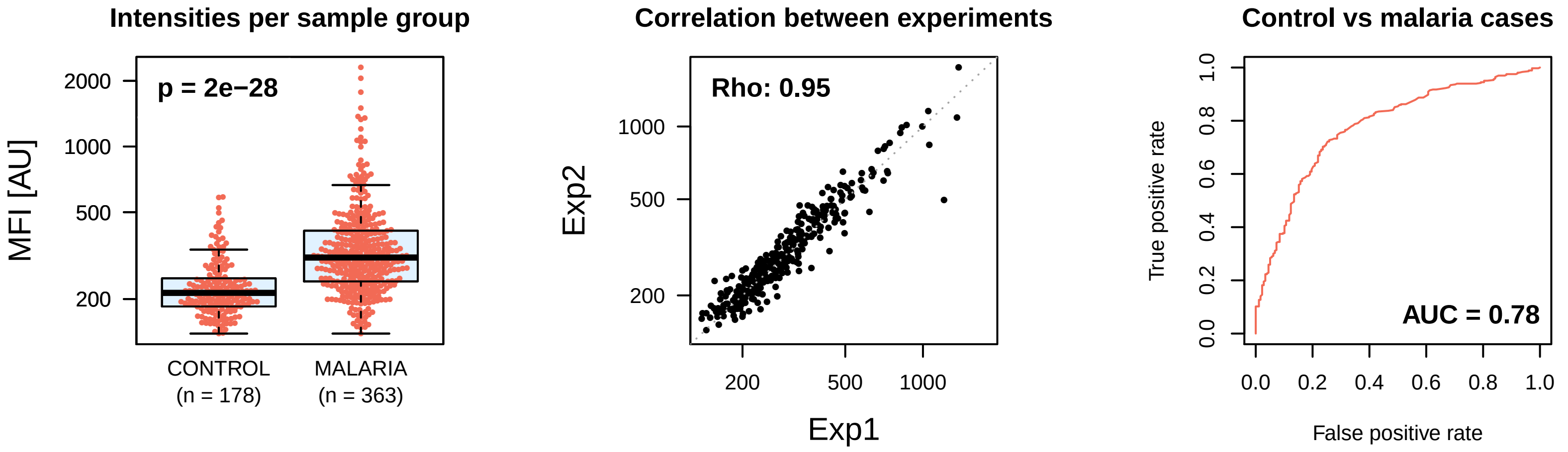

IGFBP1

Insulin like growth factor binding protein 1
Antibody: MAB675 R&D

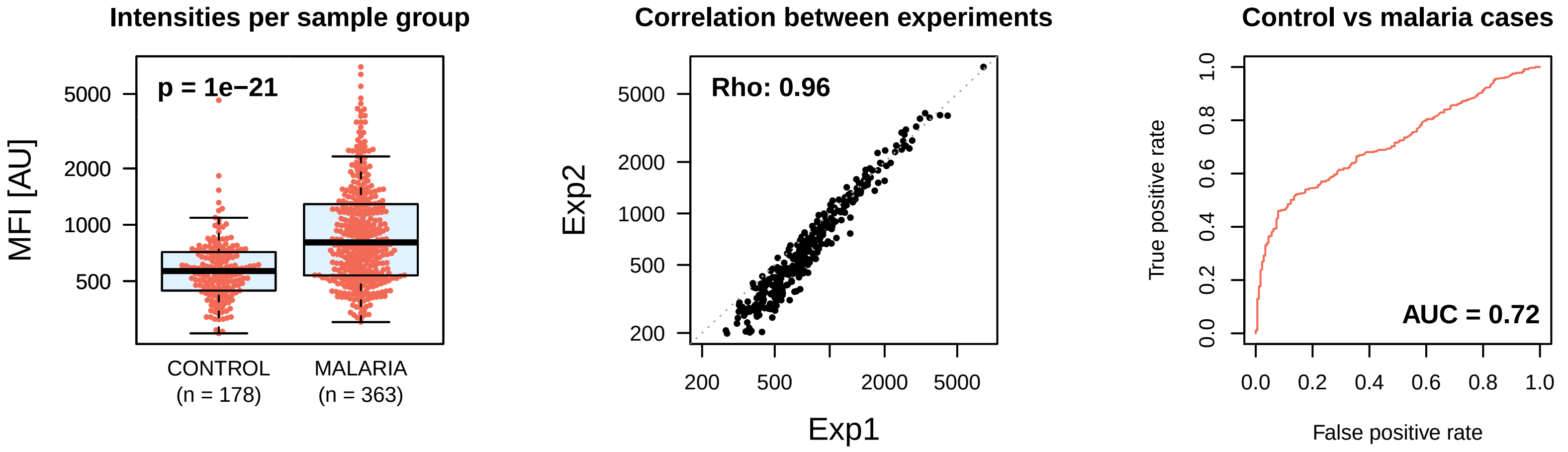

ITGAV

Integrin subunit alpha V
Antibody: HPA004856

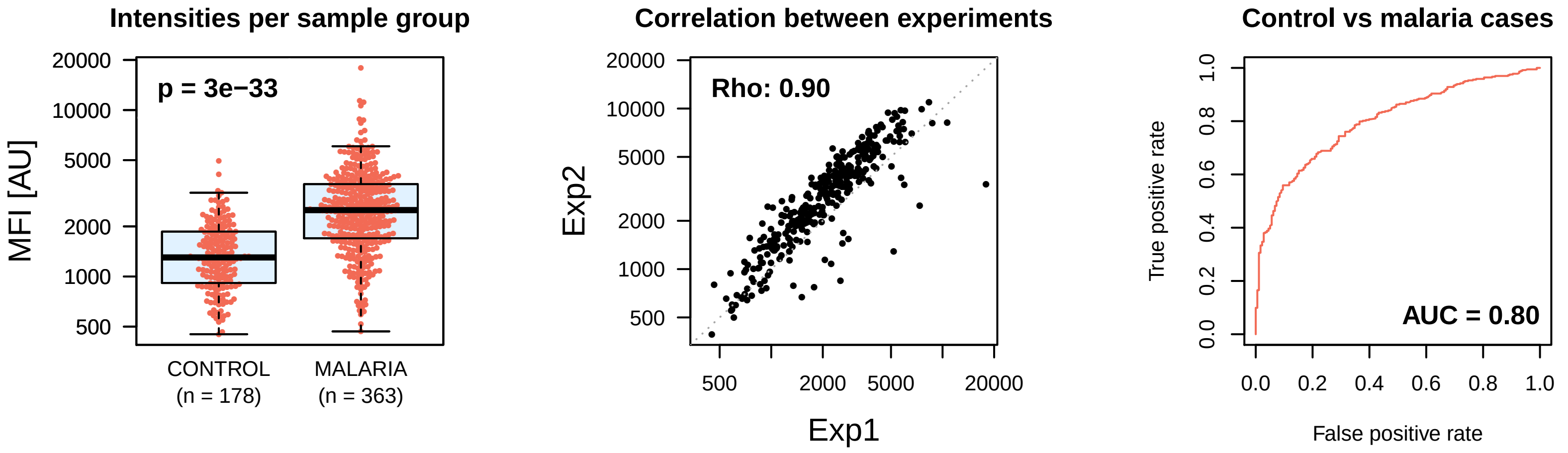

LBP

Lipopolysaccharide binding protein
Antibody: HPA001508

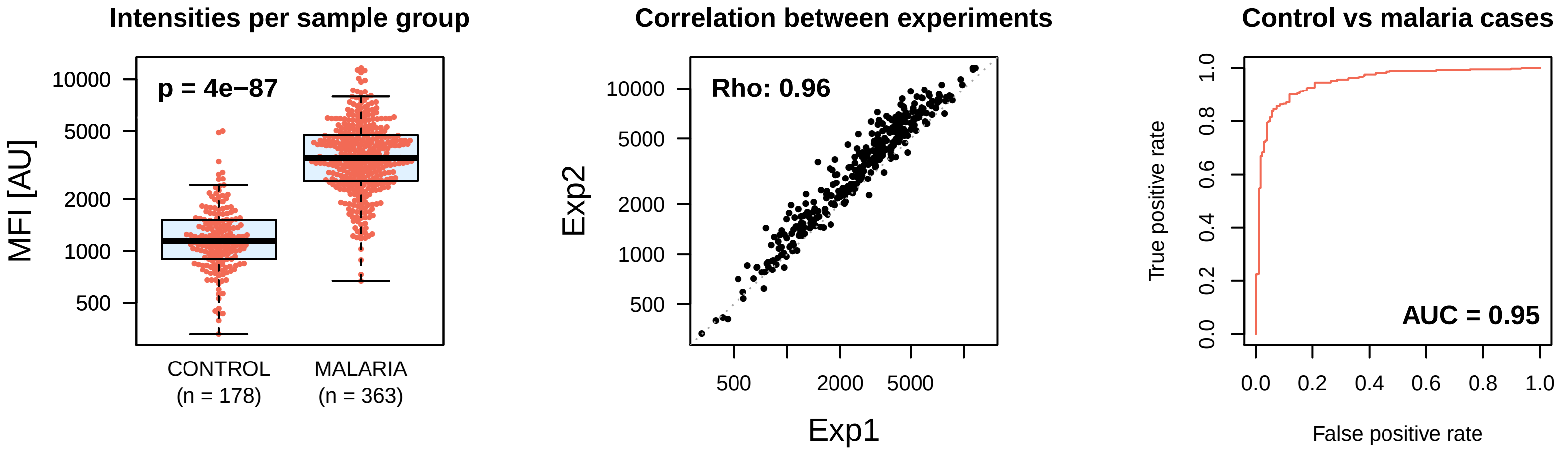

LCP1

Lymphocyte cytosolic protein 1
Antibody: HPA019493

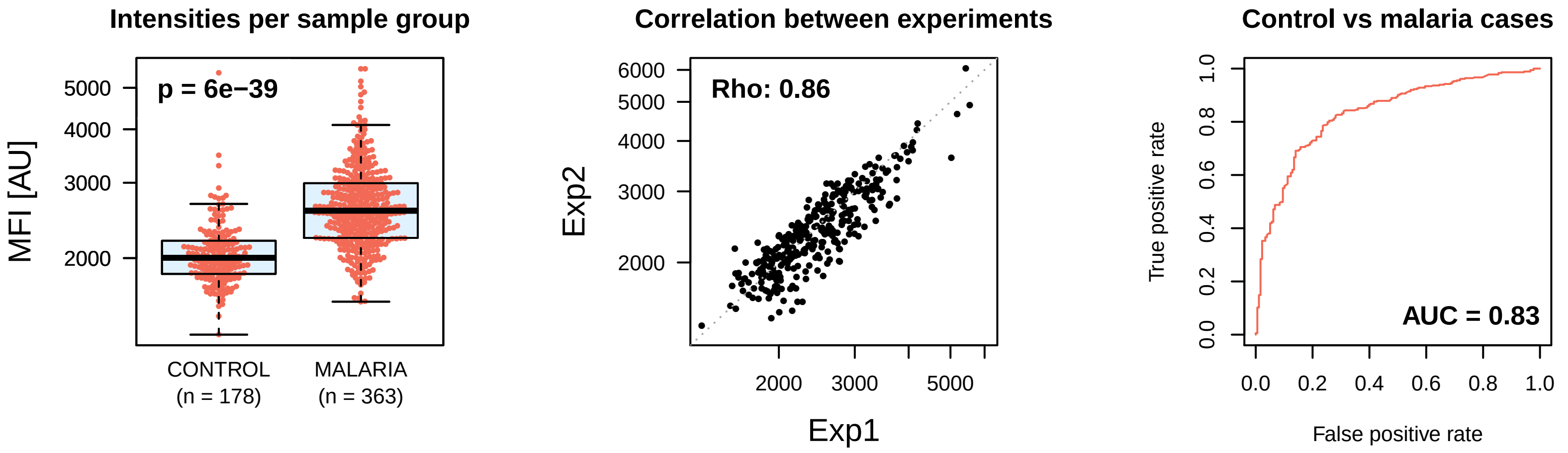

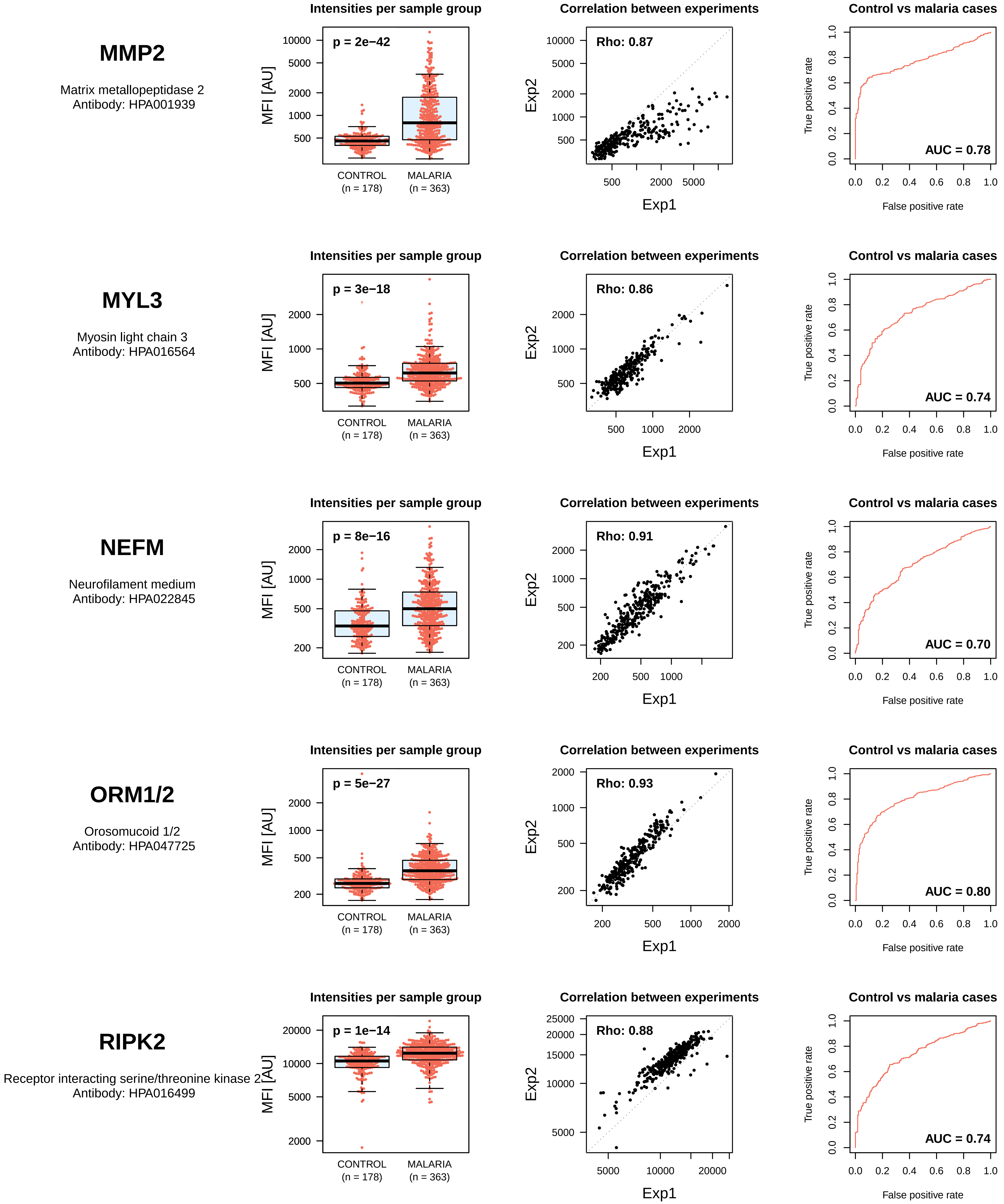
MMP2
Matrix metallopeptidase 2
Antibody: HPA001939
Intensities per sample group
p = 2e−42
MFI [AU]
10000
5000
2000
1000
500
CONTROL
(n = 178)
MALARIA
(n = 363)
Correlation between experiments
Rho: 0.87
Exp2
Exp1
500
2000
5000
Control vs malaria cases
AUC = 0.78
True positive rate
False positive rate
0.0
0.2
0.4
0.6
0.8
1.0
MYL3
Myosin light chain 3
Antibody: HPA016564
Intensities per sample group
p = 3e−18
MFI [AU]
2000
1000
500
CONTROL
(n = 178)
MALARIA
(n = 363)
Correlation between experiments
Rho: 0.86
Exp2
Exp1
500
1000
2000
Control vs malaria cases
AUC = 0.74
True positive rate
False positive rate
NEFM
Neurofilament medium
Antibody: HPA022845
Intensities per sample group
p = 8e−16
MFI [AU]
2000
1000
500
200
CONTROL
(n = 178)
MALARIA
(n = 363)
Correlation between experiments
Rho: 0.91
Exp2
Exp1
200
500
1000
2000
Control vs malaria cases
AUC = 0.70
True positive rate
False positive rate
ORM1/2
Orosomucoid 1/2
Antibody: HPA047725
Intensities per sample group
p = 5e−27
MFI [AU]
2000
1000
500
200
CONTROL
(n = 178)
MALARIA
(n = 363)
Correlation between experiments
Rho: 0.93
Exp2
Exp1
200
500
1000
2000
Control vs malaria cases
AUC = 0.80
True positive rate
False positive rate
RIPK2
Receptor interacting serine/threonine kinase 2
Antibody: HPA016499
Intensities per sample group
p = 1e−14
MFI [AU]
20000
10000
5000
2000
CONTROL
(n = 178)
MALARIA
(n = 363)
Correlation between experiments
Rho: 0.88
Exp2
Exp1
25000
20000
15000
10000
5000
Control vs malaria cases
AUC = 0.74
True positive rate
False positive rate

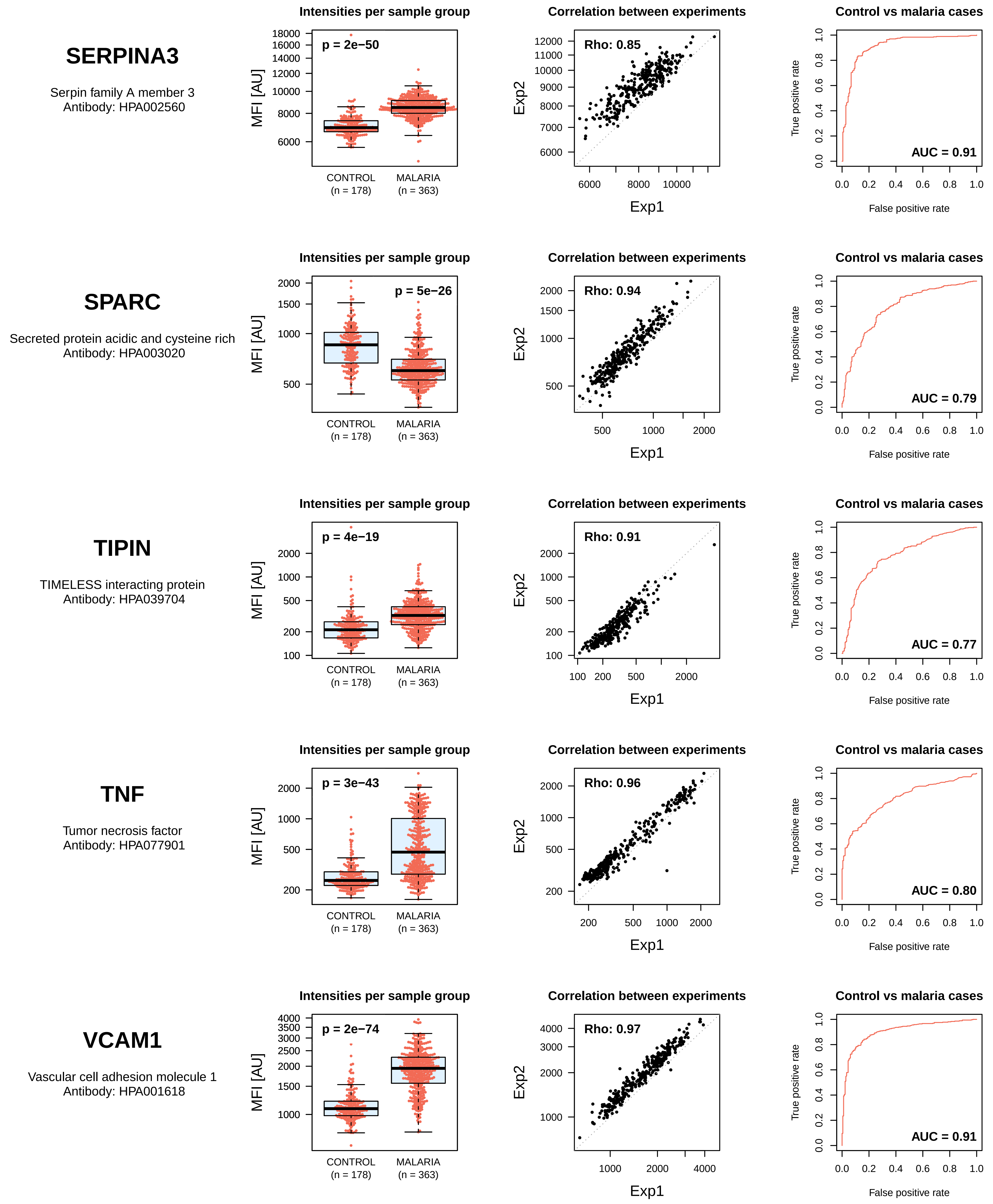
SERPINA3
Serpin family A member 3
Antibody: HPA002560
Intensities per sample group
p = 2e−50
MFI [AU]
CONTROL (n = 178)
MALARIA (n = 363)
Correlation between experiments
Rho: 0.85
Exp1
Exp2
Control vs malaria cases
AUC = 0.91
True positive rate
False positive rate
SPARC
Secreted protein acidic and cysteine rich
Antibody: HPA003020
Intensities per sample group
p = 5e−26
MFI [AU]
CONTROL (n = 178)
MALARIA (n = 363)
Correlation between experiments
Rho: 0.94
Exp1
Exp2
Control vs malaria cases
AUC = 0.79
True positive rate
False positive rate
TIPIN
TIMELESS interacting protein
Antibody: HPA039704
Intensities per sample group
p = 4e−19
MFI [AU]
CONTROL (n = 178)
MALARIA (n = 363)
Correlation between experiments
Rho: 0.91
Exp1
Exp2
Control vs malaria cases
AUC = 0.77
True positive rate
False positive rate
TNF
Tumor necrosis factor
Antibody: HPA077901
Intensities per sample group
p = 3e−43
MFI [AU]
CONTROL (n = 178)
MALARIA (n = 363)
Correlation between experiments
Rho: 0.96
Exp1
Exp2
Control vs malaria cases
AUC = 0.80
True positive rate
False positive rate
VCAM1
Vascular cell adhesion molecule 1
Antibody: HPA001618
Intensities per sample group
p = 2e−74
MFI [AU]
CONTROL (n = 178)
MALARIA (n = 363)
Correlation between experiments
Rho: 0.97
Exp1
Exp2
Control vs malaria cases
AUC = 0.91
True positive rate
False positive rate

VWF

von Willebrand factor
Antibody: HPA002082

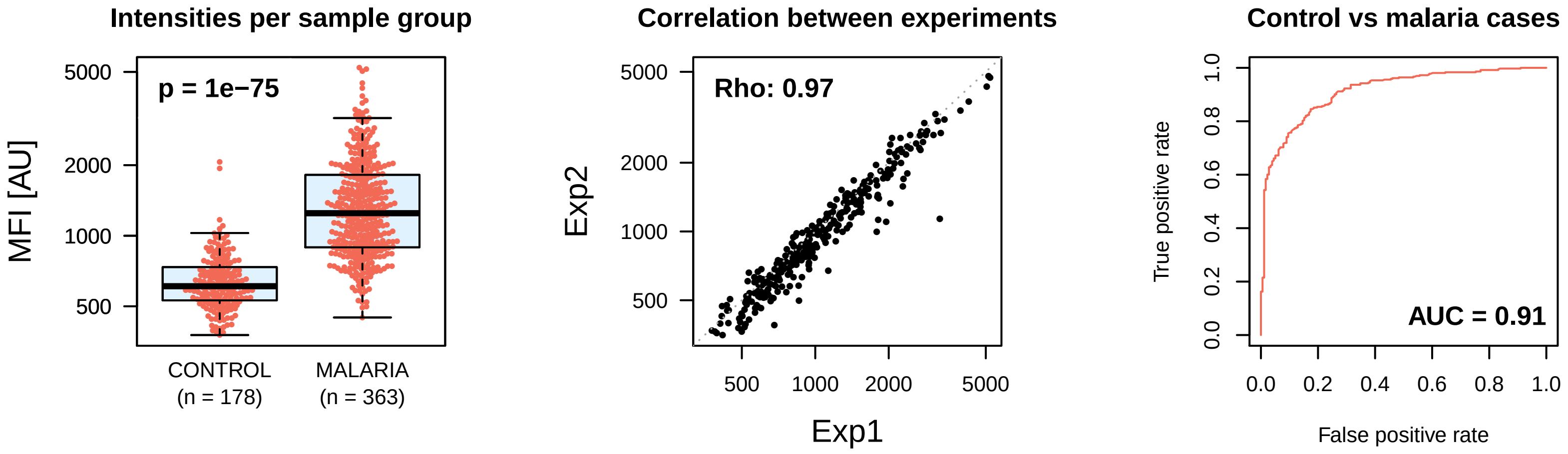

Supplement: Supplementary file 8 — Additional file 8. Information about proteins discriminating between controls and malaria patients. One panel for each of the proteins presented as potential markers for separating community controls from malaria cases with a p-value < 10−14. The panel includes one boxplot with protein levels for the group of controls and the malaria cases together with the Spearman’s correlation Rho for that protein between the two experiments (Exp 1 and Exp 2) and the classification power of the protein represented on ROC curves (including AUC values). [file 12936_2018_2576_MOESM8_ESM.pdf]
