# Supplementary material for: Levels of human proteins in plasma associated with acute paediatric malaria
Source: Malar J. 2018 Nov 15;17:426. doi: 10.1186/s12936-018-2576-y (PMC6238294; doi:10.1186/s12936-018-2576-y)

Additional file 9. Information about proteins with divergent levels between mild and severe malaria patients

ADAMTS13

ADAM metallopeptidase w. thrombospondin type 1 motif 13
Antibody: HPA042014

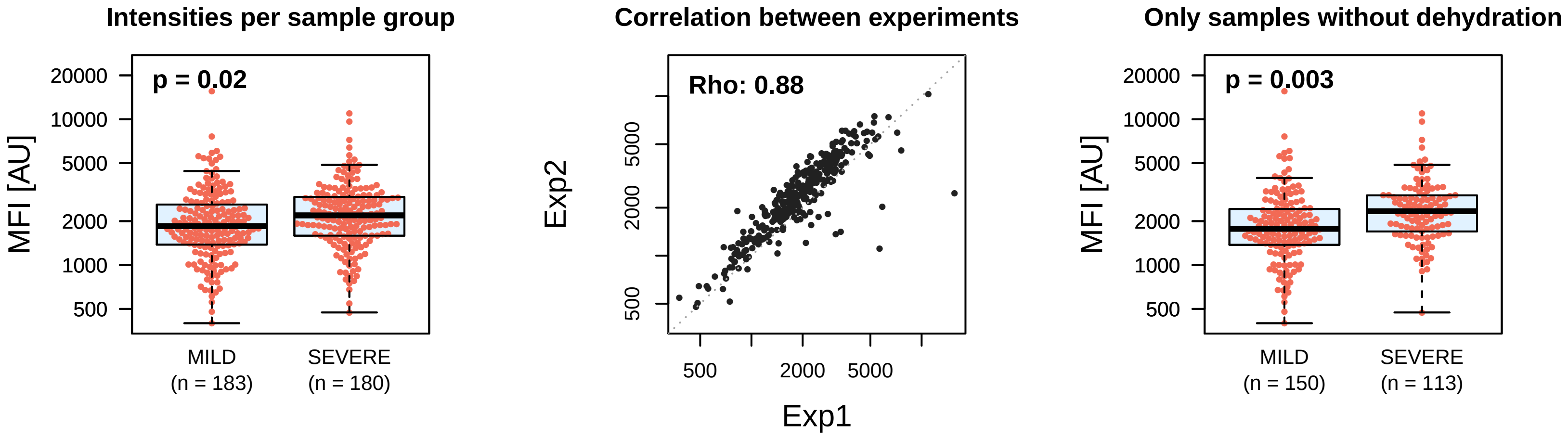

AGT

Angiotensinogen
Antibody: MAB3156 R&D

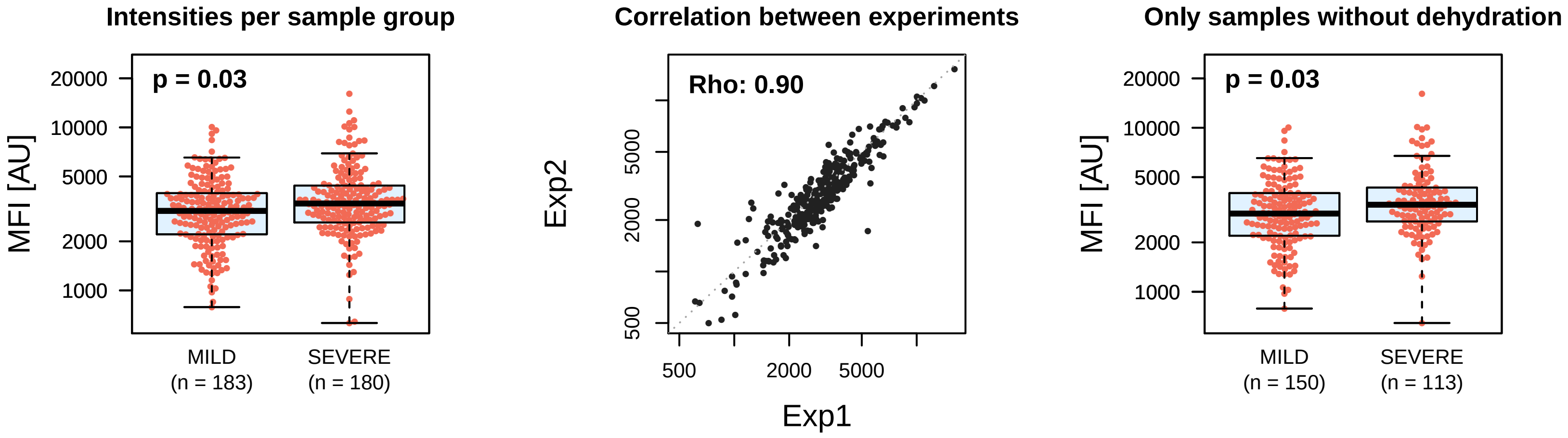

ANK1

Ankyrin 1
Antibody: HPA004842

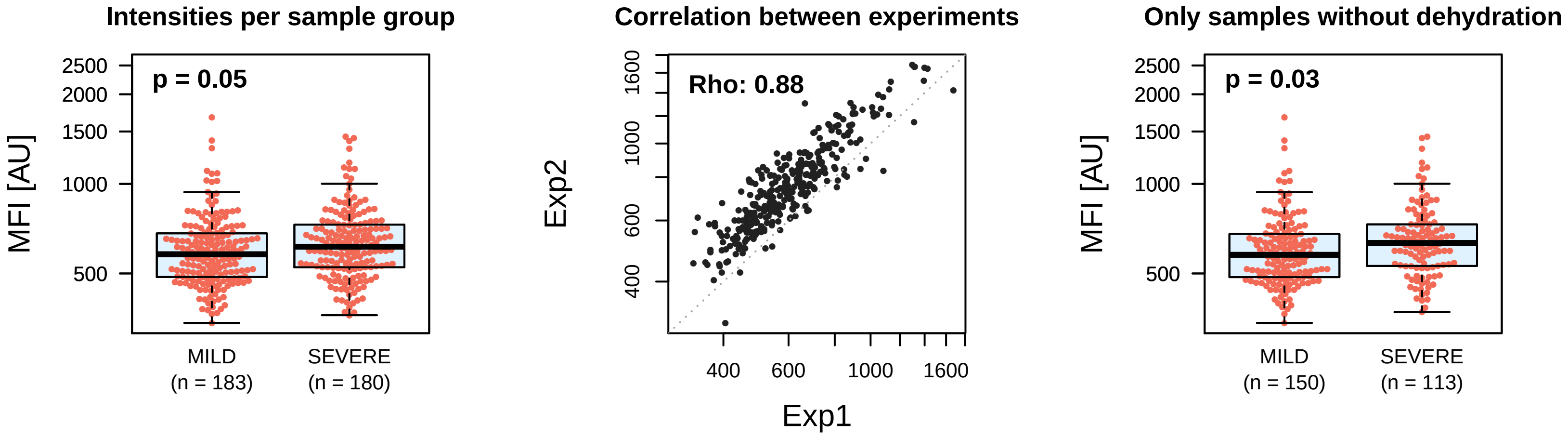

BPGM

Bisphosphoglycerate mutase
Antibody: HPA016493

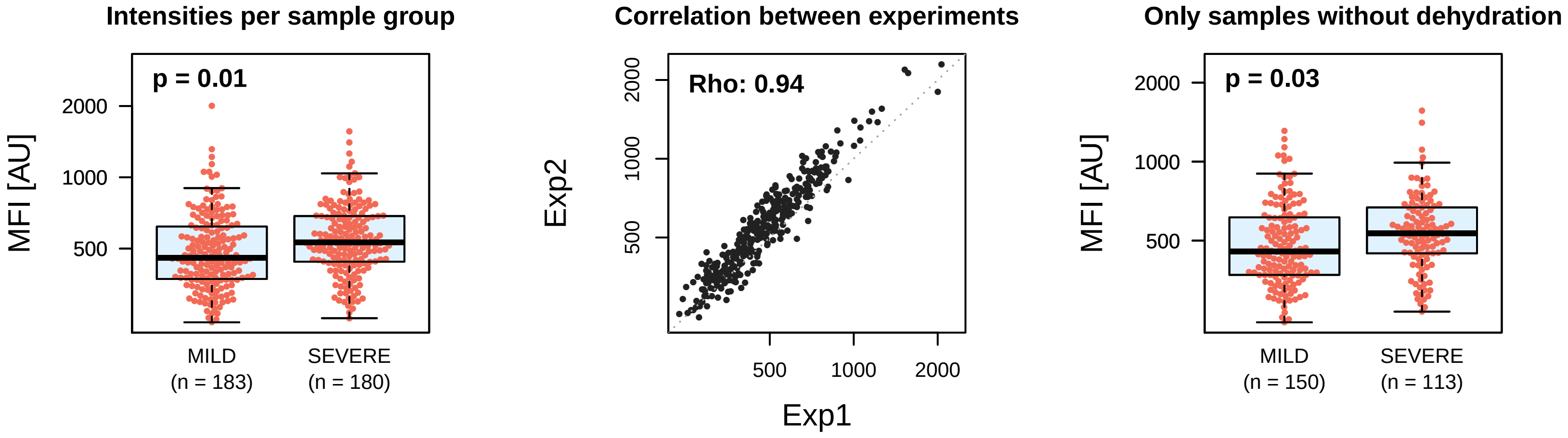

CA2

Carbonic anhydrase 2
Antibody: HPA071085

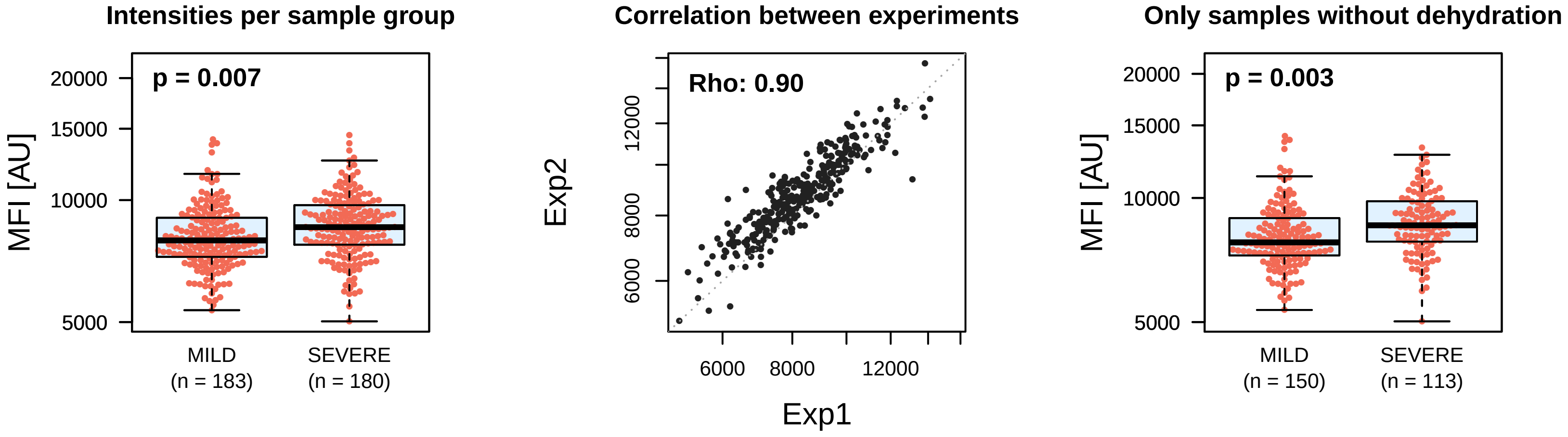

CALCA

Calcitonin related polypeptide alpha
Antibody: HPA064453

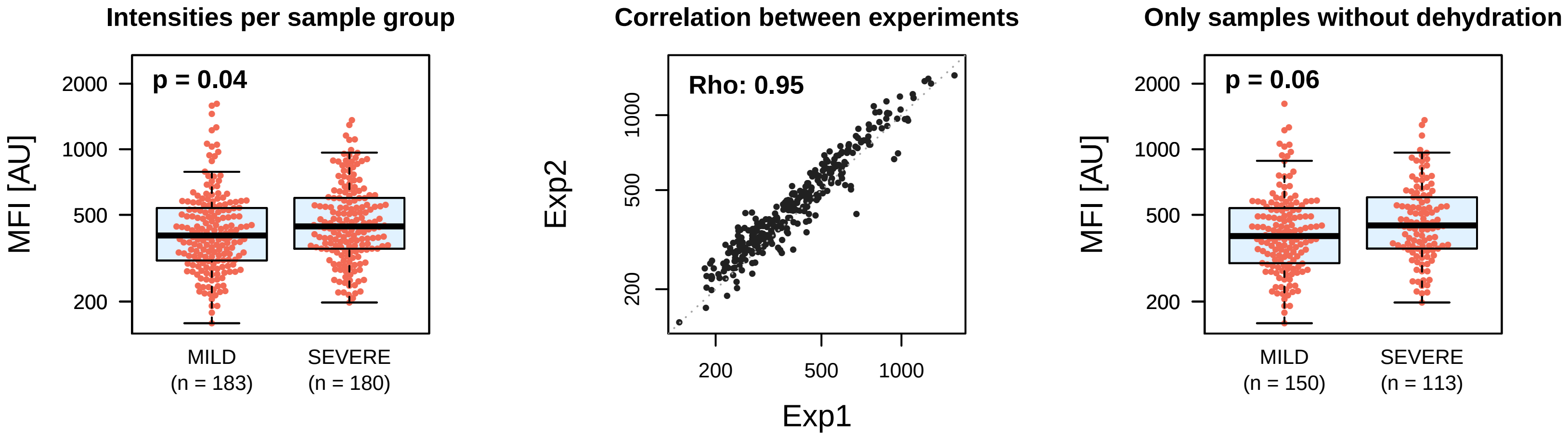

CD14

CD14 molecule
Antibody: HPA001887

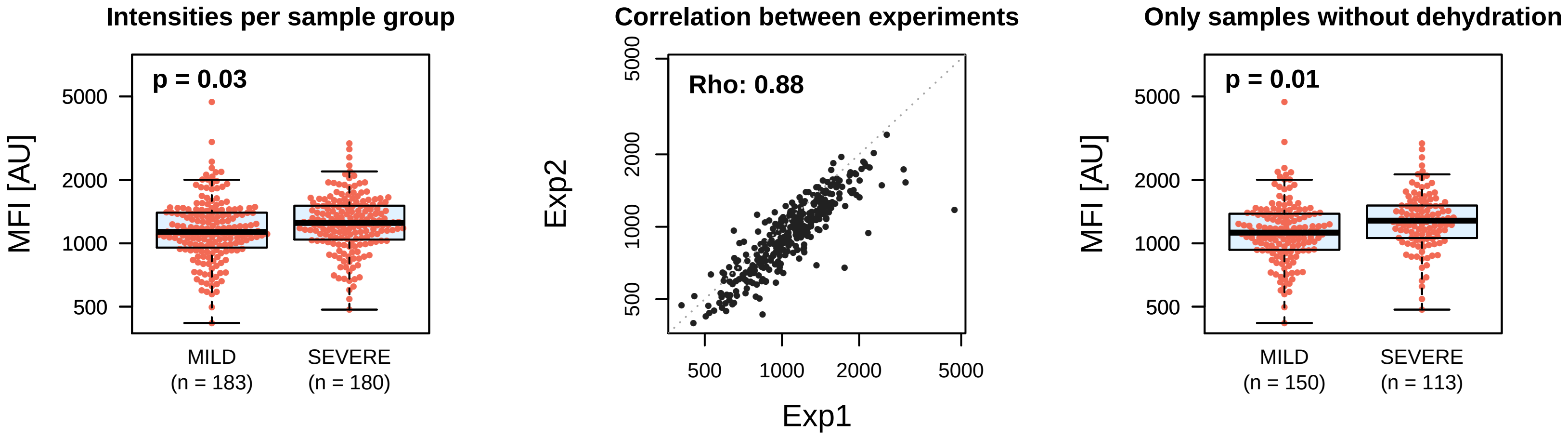

CD80

CD80 molecule
Antibody: HPA039851

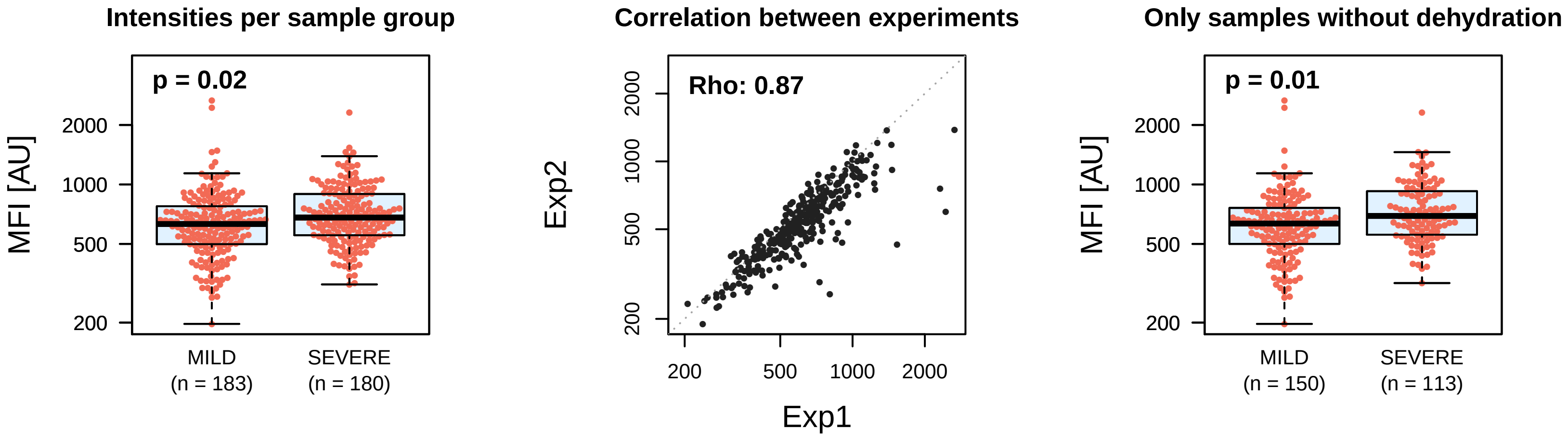

CDK14

Cyclin dependent kinase 14
Antibody: HPA015267

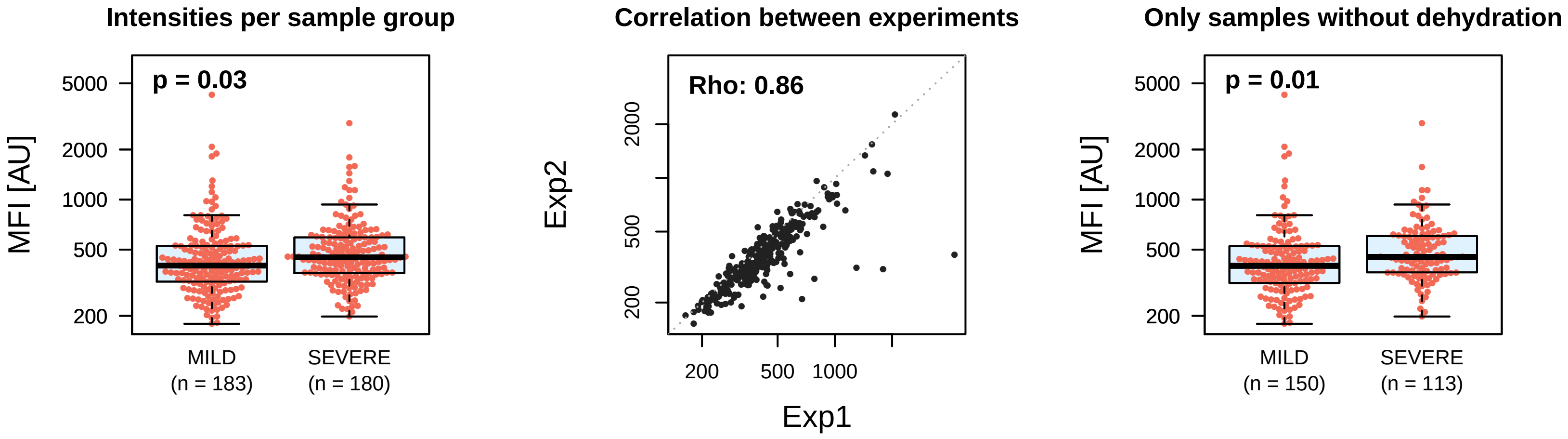

CEBPA

CCAAT/enhancer binding protein alpha
Antibody: HPA052734

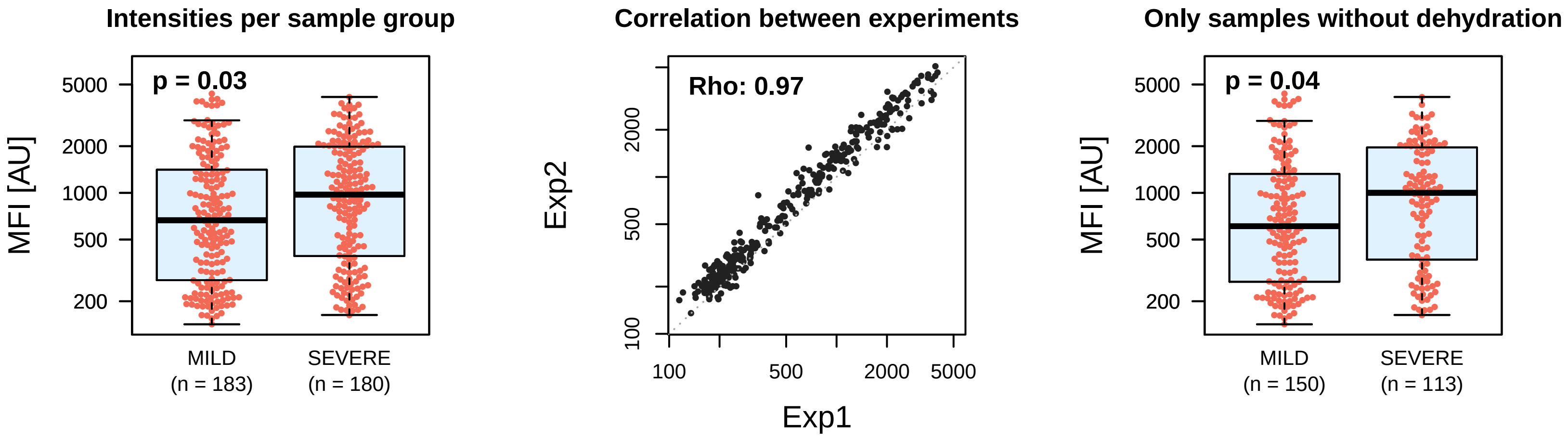

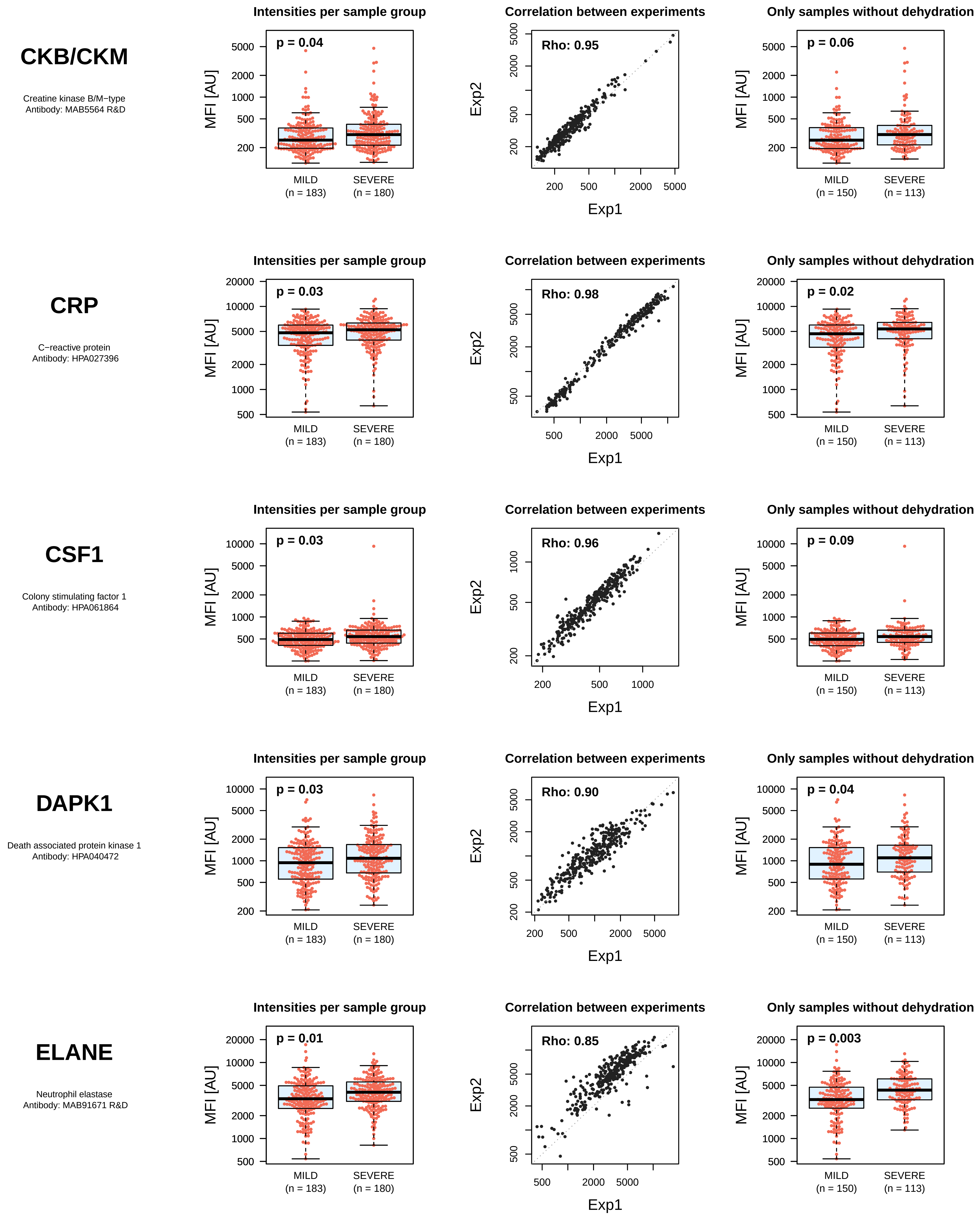
CKB/CKM
Creatine kinase B/M−type
Antibody: MAB5564 R&D
Intensities per sample group
p = 0.04
MFI [AU]
MILD (n = 183)
SEVERE (n = 180)
Correlation between experiments
Rho: 0.95
Exp1
Exp2
Only samples without dehydration
p = 0.06
MILD (n = 150)
SEVERE (n = 113)
CRP
C−reactive protein
Antibody: HPA027396
Intensities per sample group
p = 0.03
Correlation between experiments
Rho: 0.98
Only samples without dehydration
p = 0.02
CSF1
Colony stimulating factor 1
Antibody: HPA061864
Intensities per sample group
p = 0.03
Correlation between experiments
Rho: 0.96
Only samples without dehydration
p = 0.09
DAPK1
Death associated protein kinase 1
Antibody: HPA040472
Intensities per sample group
p = 0.03
Correlation between experiments
Rho: 0.90
Only samples without dehydration
p = 0.04
ELANE
Neutrophil elastase
Antibody: MAB91671 R&D
Intensities per sample group
p = 0.01
Correlation between experiments
Rho: 0.85
Only samples without dehydration
p = 0.003

EPB41L2

Erythrocyte membrane protein band 4.1 like 2
Antibody: HPA005730

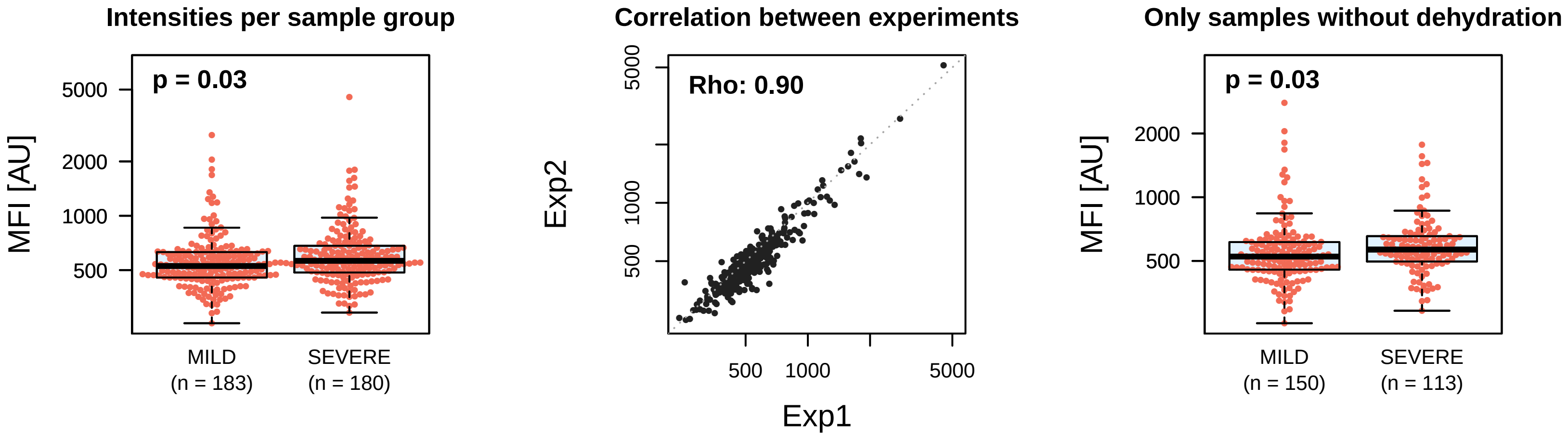

GYPC

Glycophorin C (Gerbich blood group)
Antibody: HPA008965

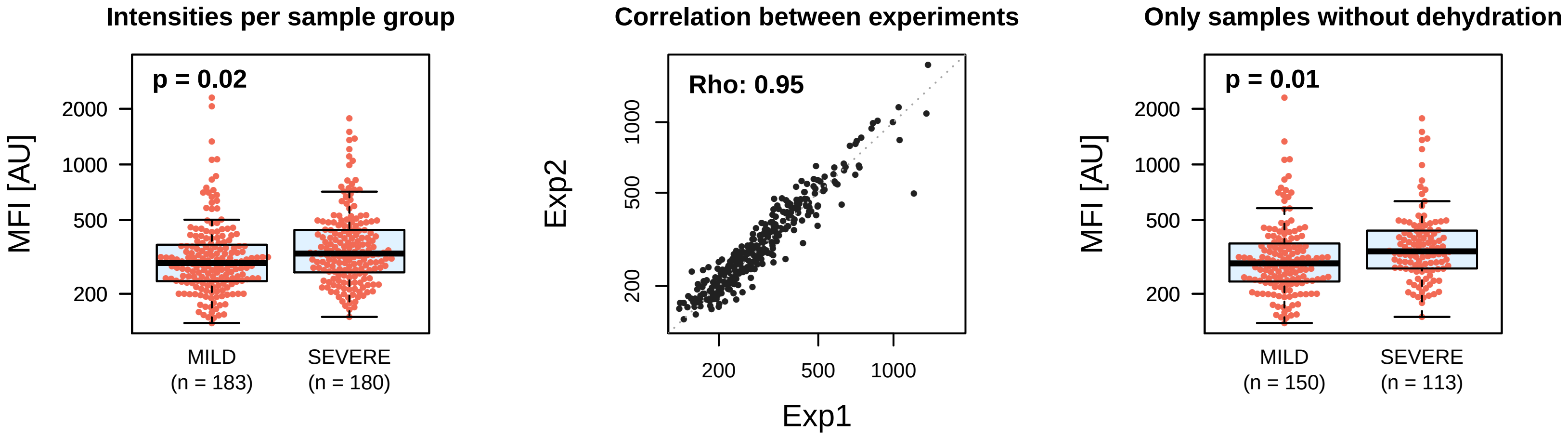

HAP1

Huntingtin associated protein 1
Antibody: HPA053019

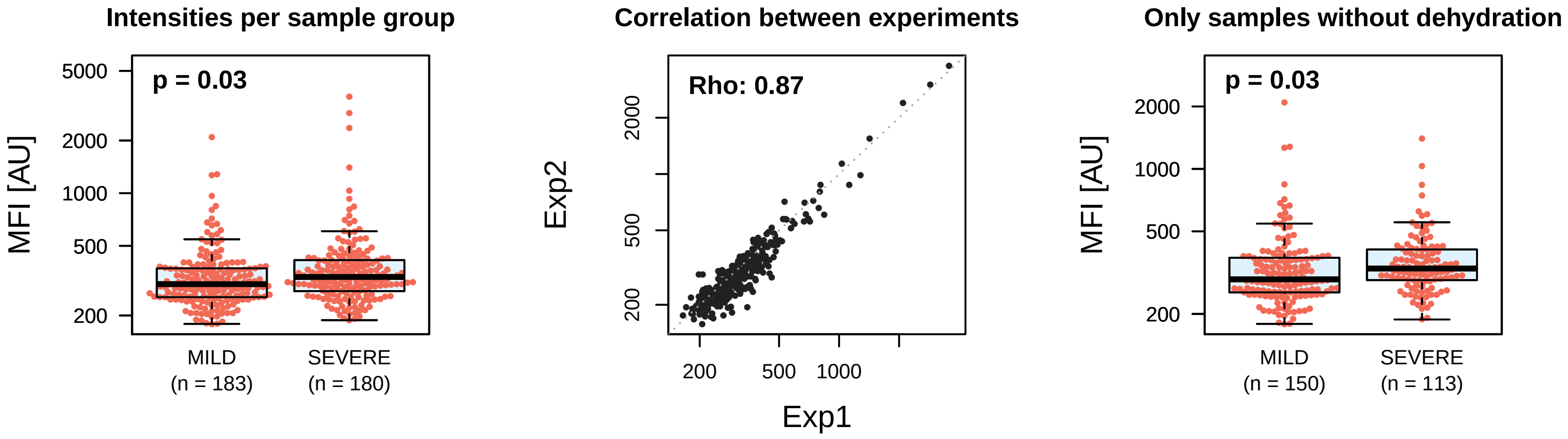

HBA1/2

Hemoglobin subunit alpha 1/2
Antibody: HPA043780

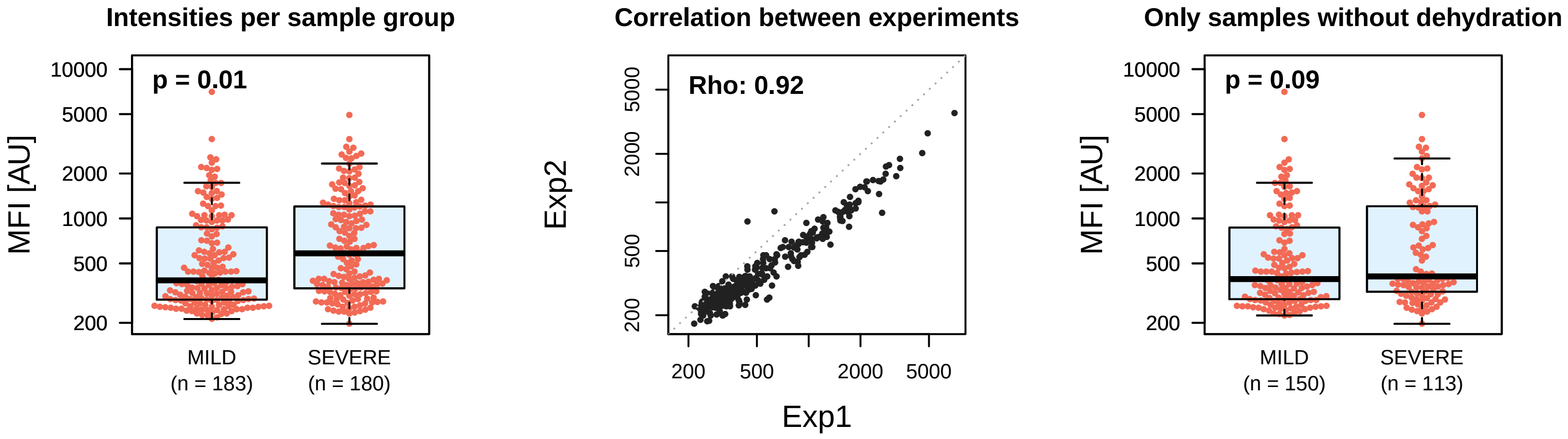

IGFBP1

Insulin like growth factor binding protein 1
Antibody: MAB675 R&D

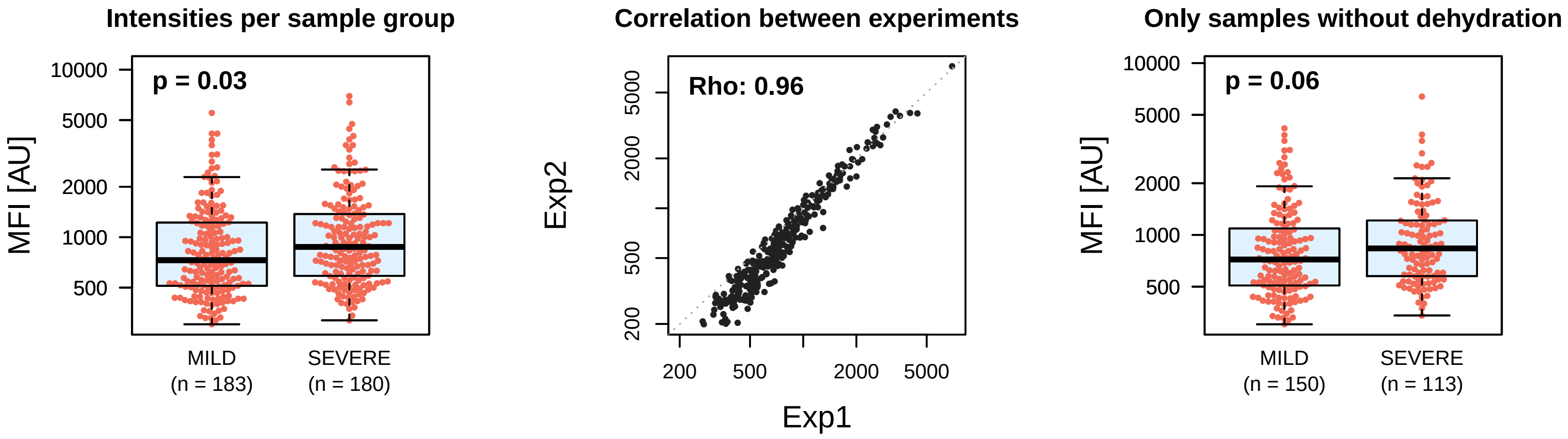

ITGAV

Integrin subunit alpha V
Antibody: HPA004856

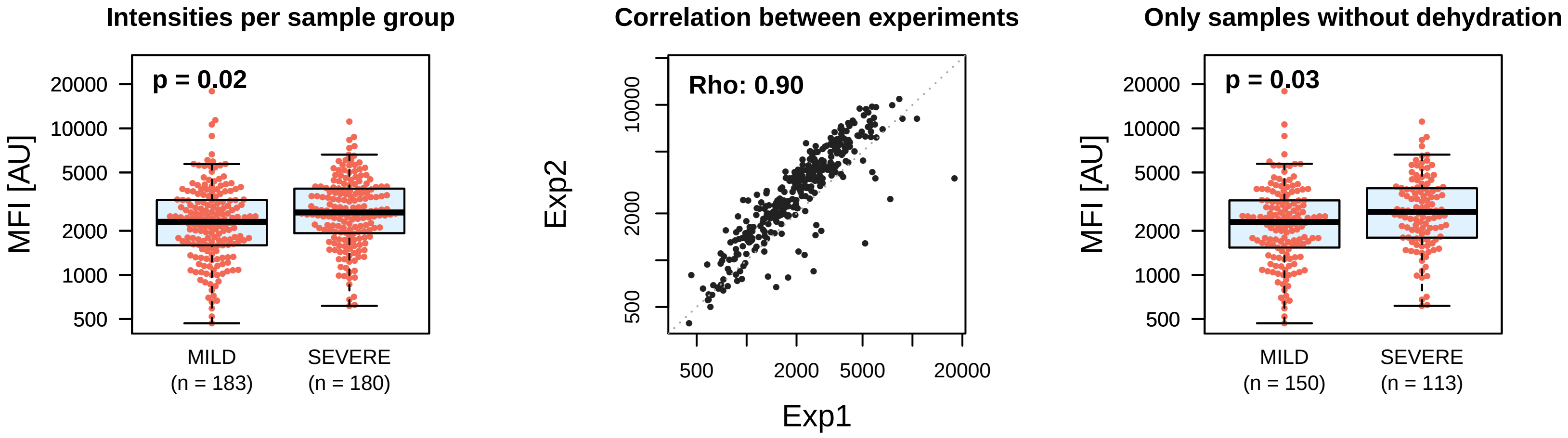

LBP

Lipopolysaccharide binding protein
Antibody: HPA001508

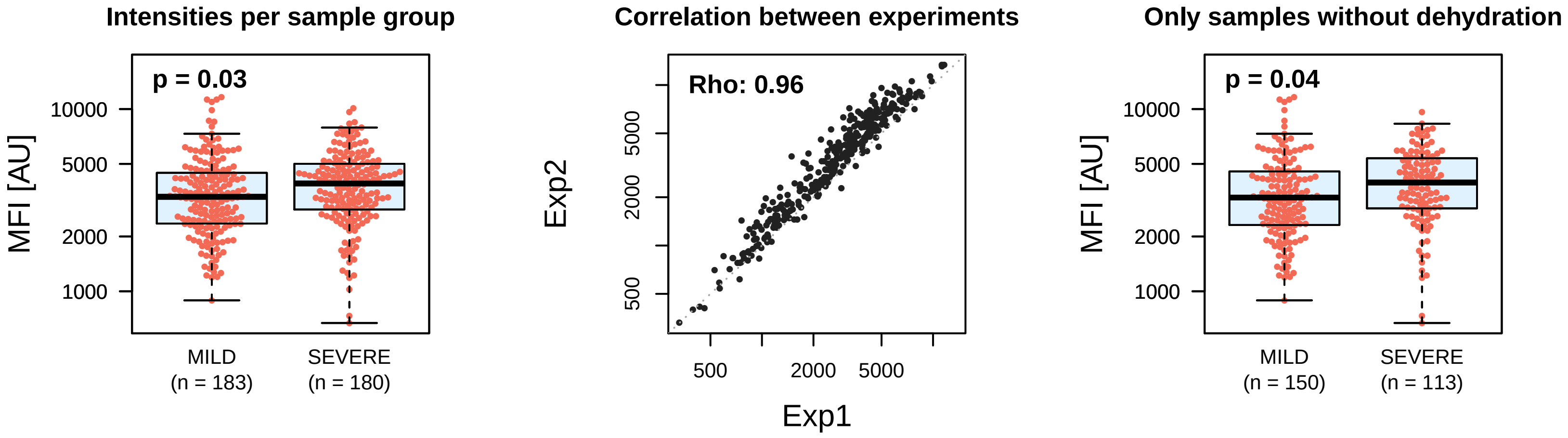

MMP2

Matrix metallopeptidase 2
Antibody: HPA001939

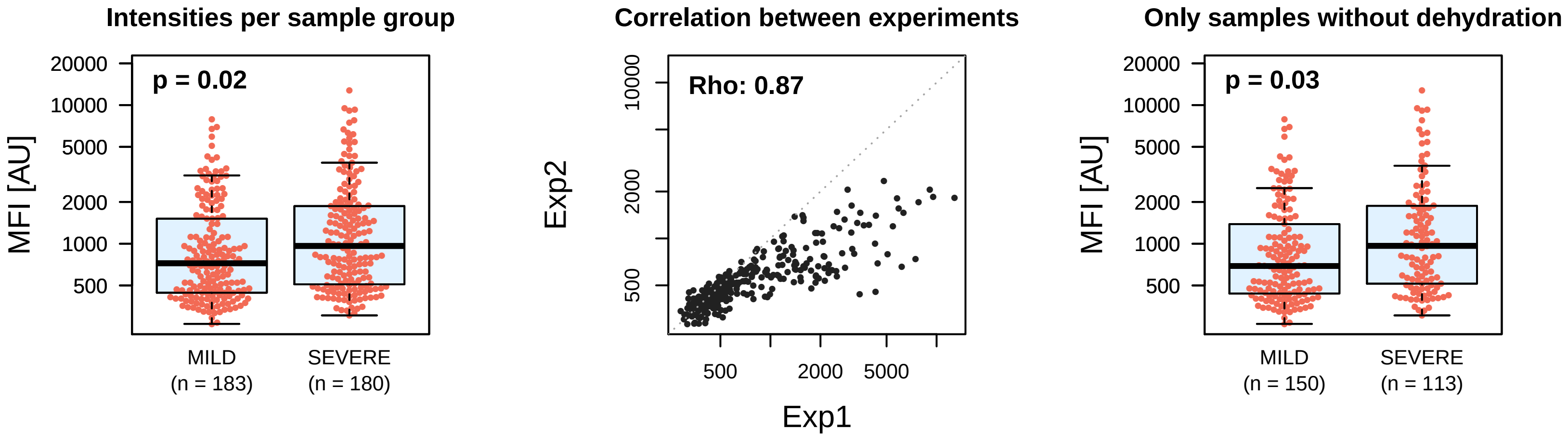

MMP9

Matrix metallopeptidase 9
Antibody: HPA001238

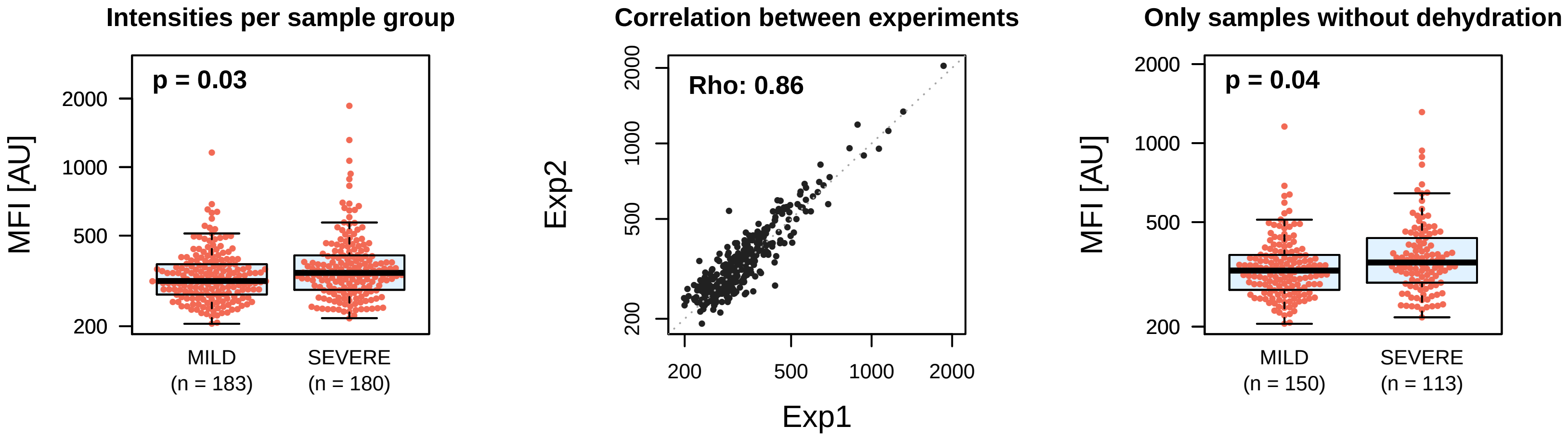

MPP1

Membrane palmitoylated protein 1
Antibody: HPA076675

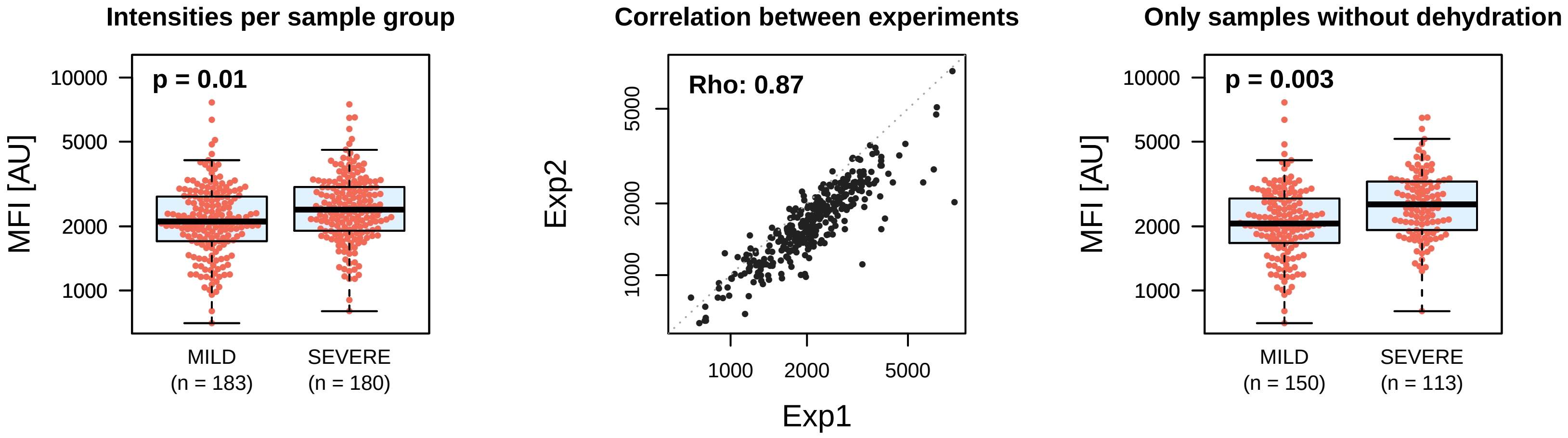

MYL3

Myosin light chain 3
Antibody: HPA016564

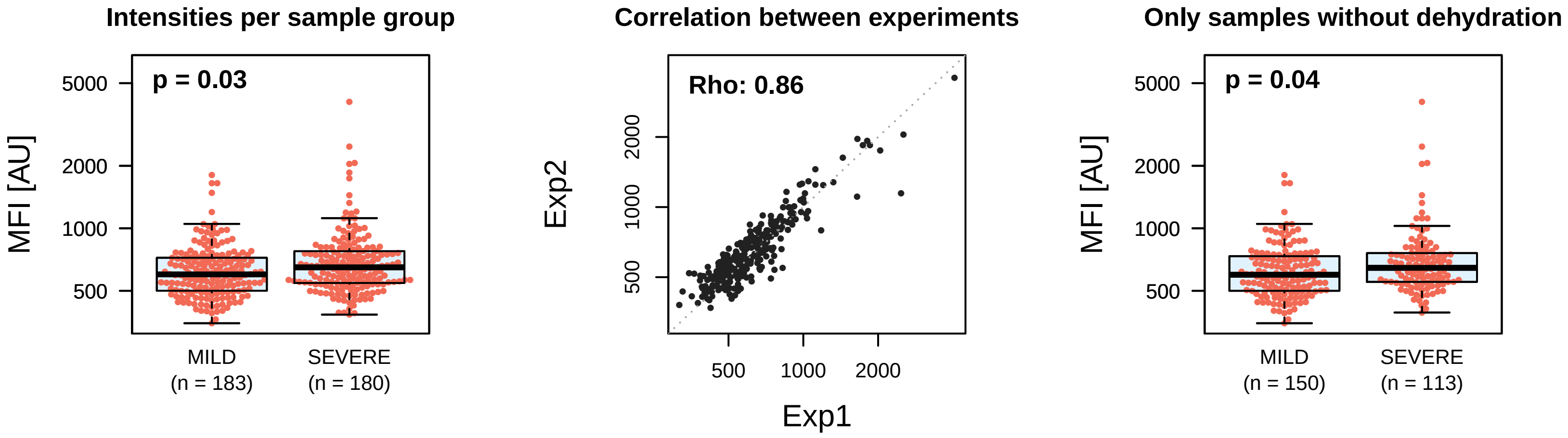

MYO15A

Myosin XVA
Antibody: HPA039770

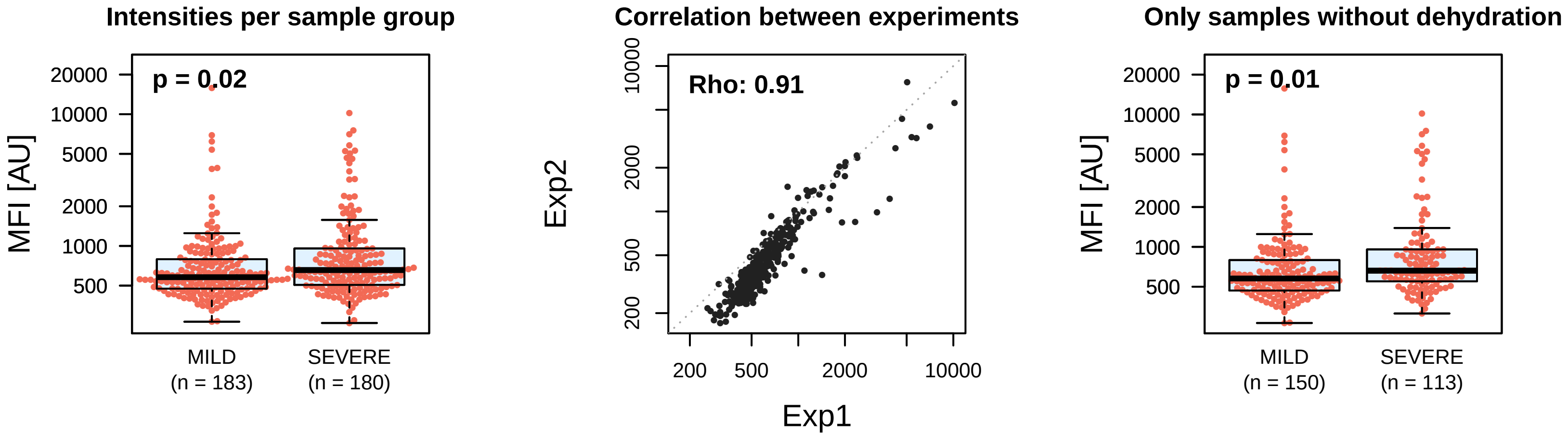

NEFM

Neurofilament medium
Antibody: HPA022845

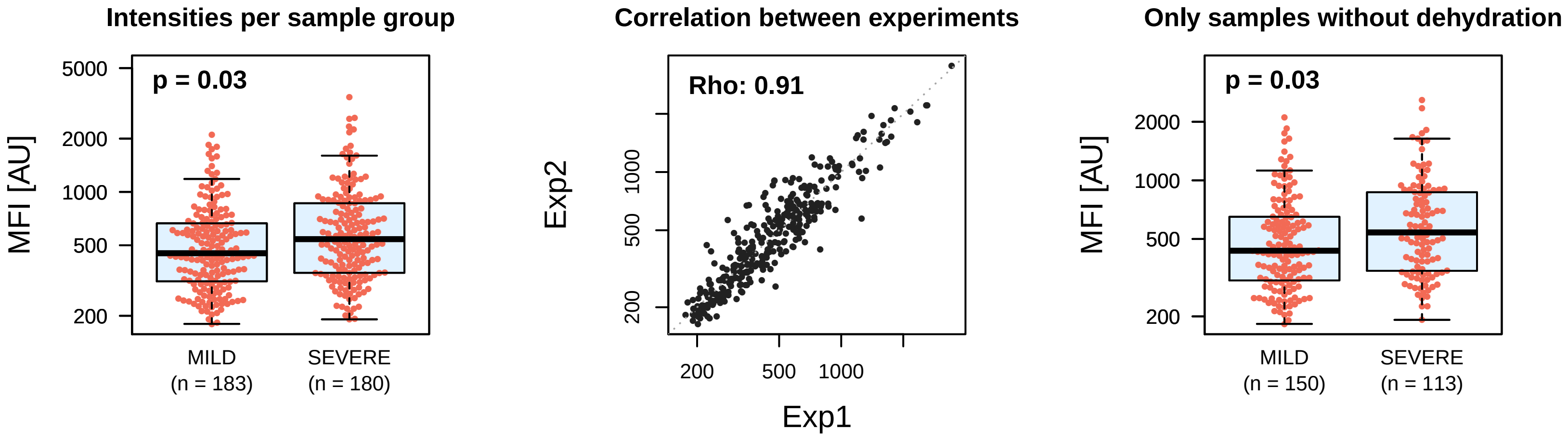

NGF

Nerve growth factor
Antibody: HPA063135

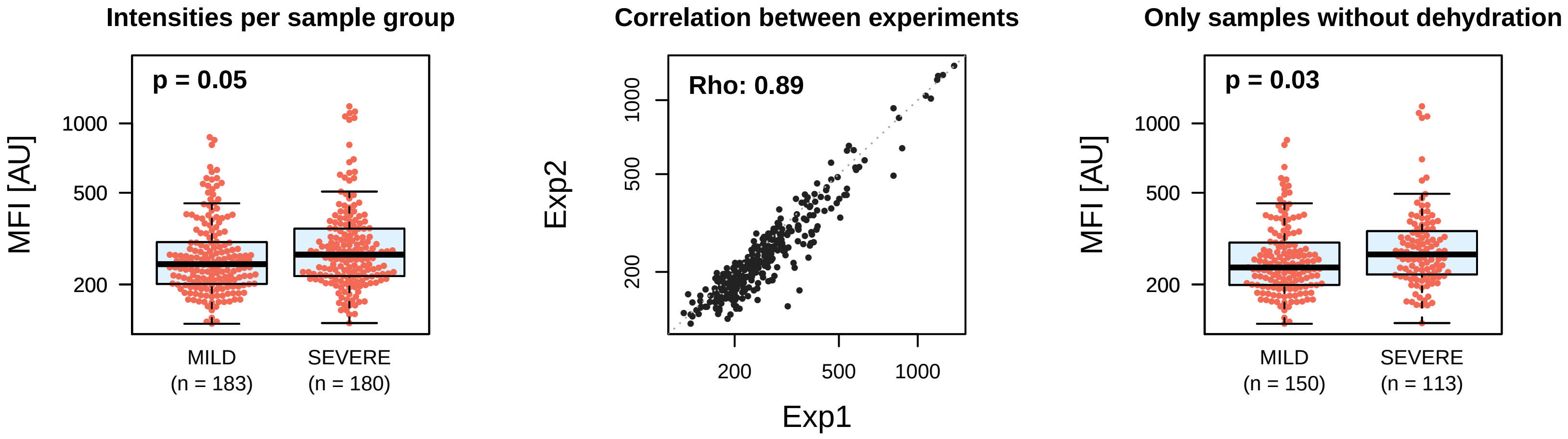

RIPK2

Receptor interacting serine/threonine kinase 2
Antibody: HPA015764

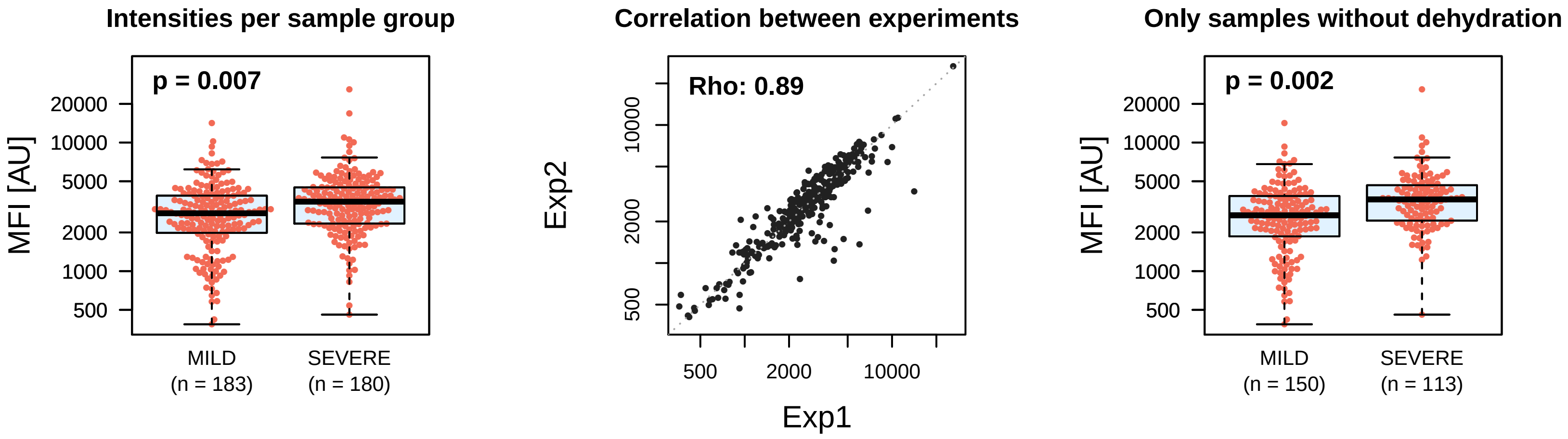

TIPIN

TIMELESS interacting protein
Antibody: HPA039704

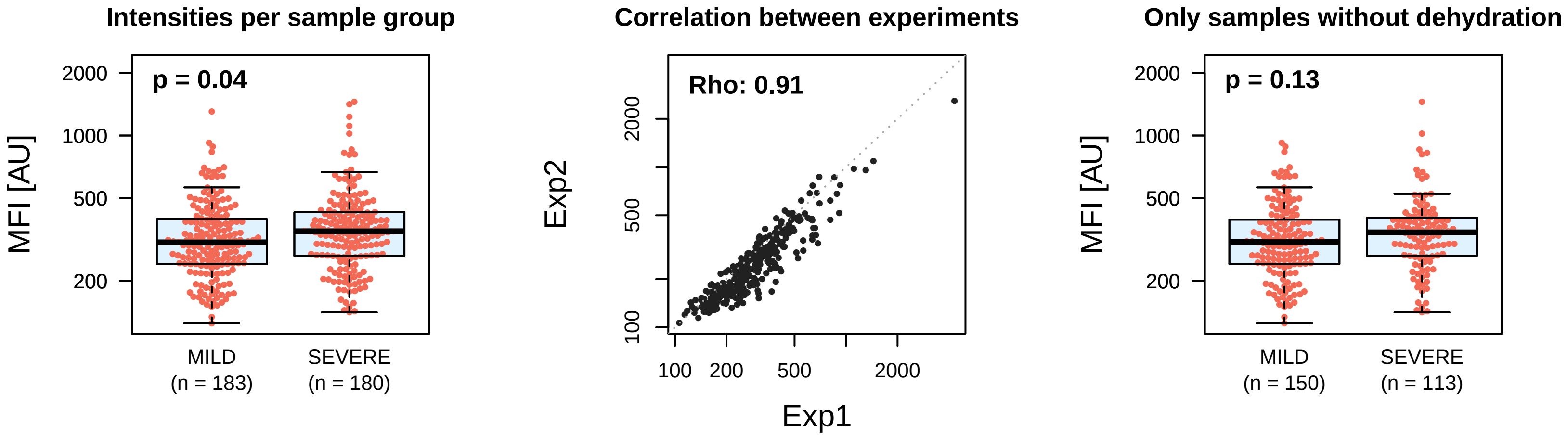

TNF

Tumor necrosis factor
Antibody: HPA055037

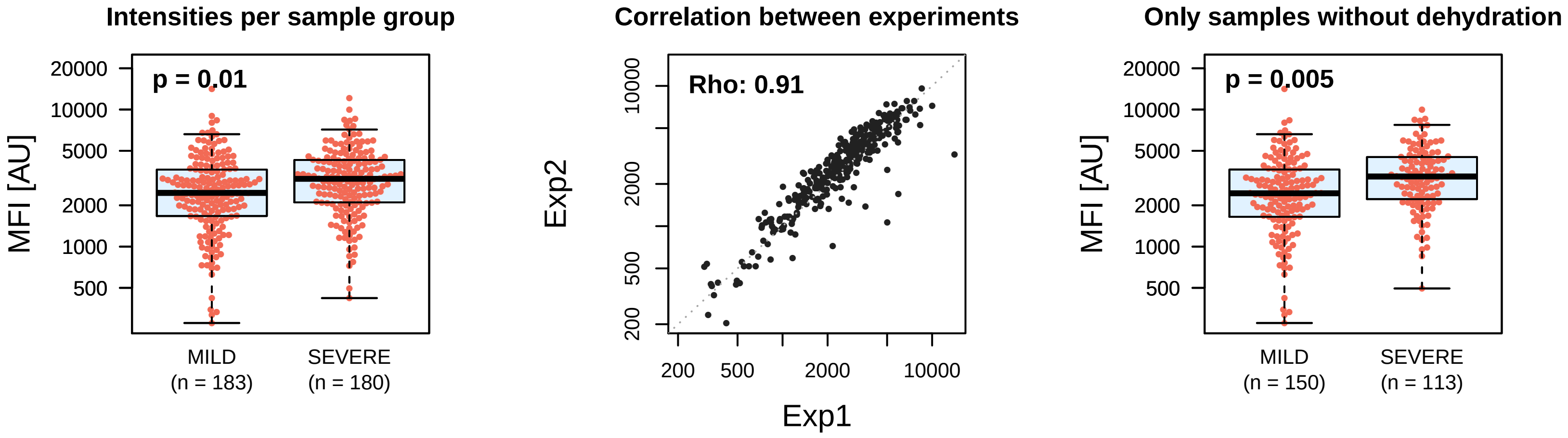

TNFRSF1B

TNF receptor superfamily member 1B
Antibody: HPA004796

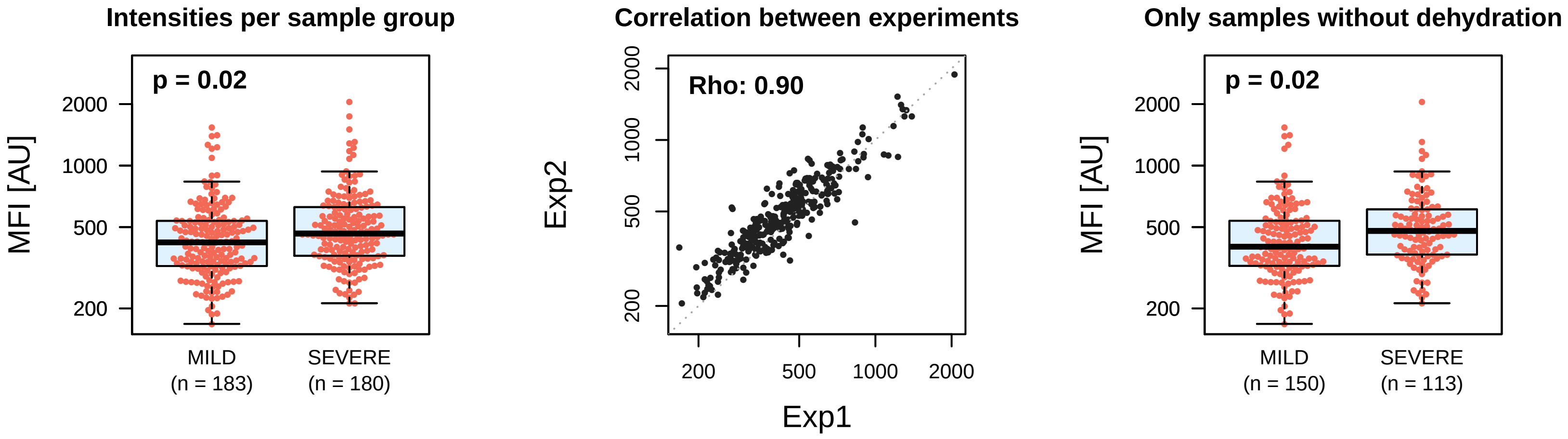

TNFSF13B

TNF superfamily member 13b
Antibody: HPA030526

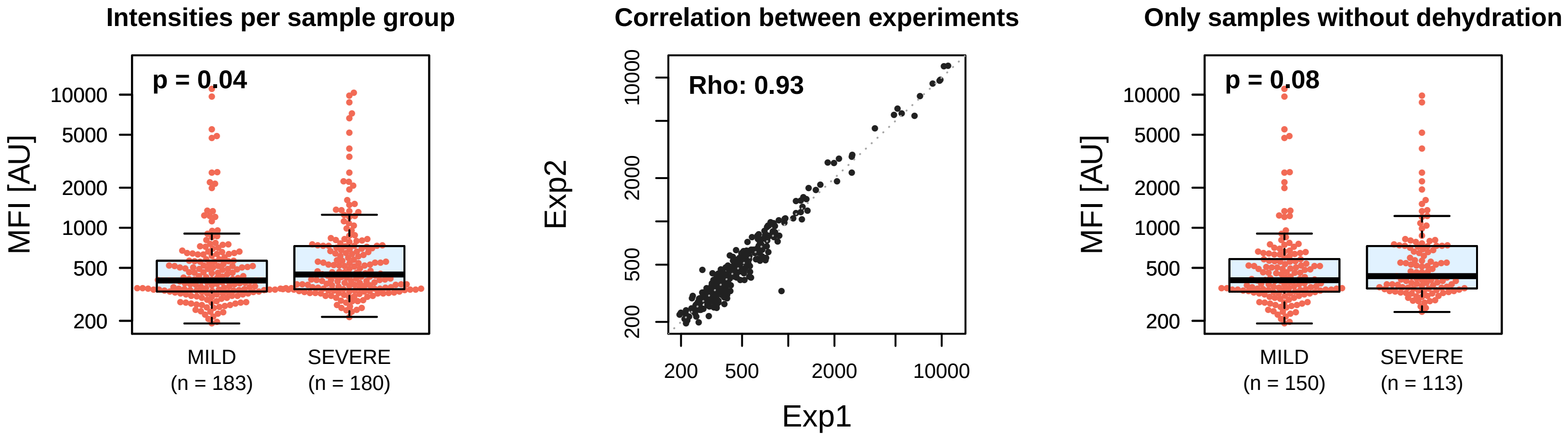

VCAM1

Vascular cell adhesion molecule 1
Antibody: HPA001618

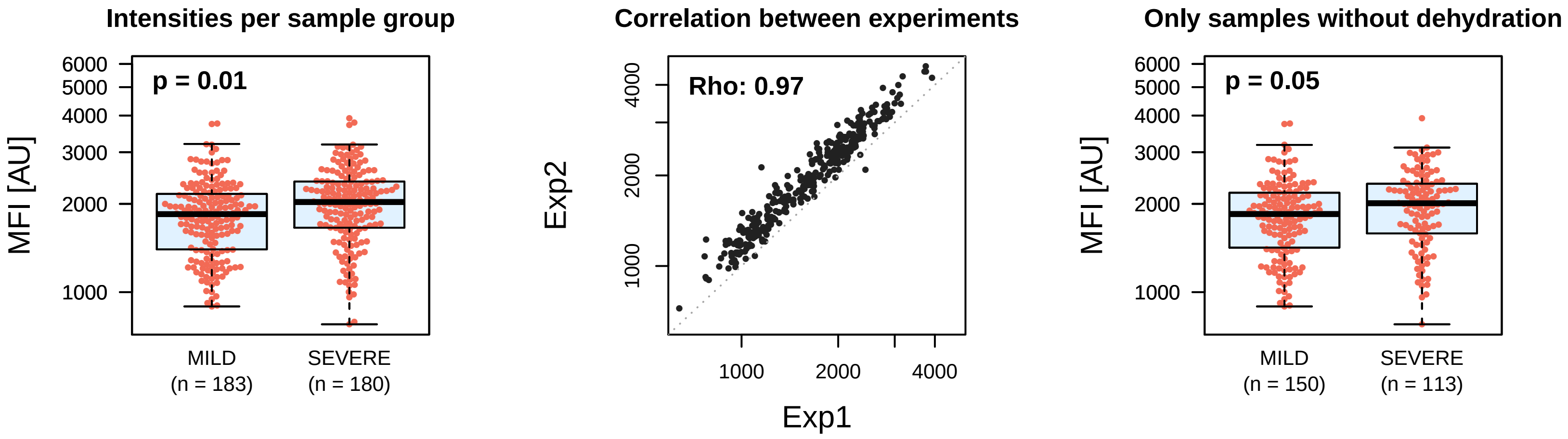

VWF

von Willebrand factor
Antibody: HPA002082

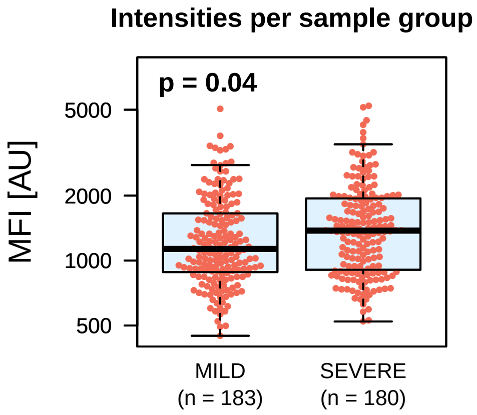

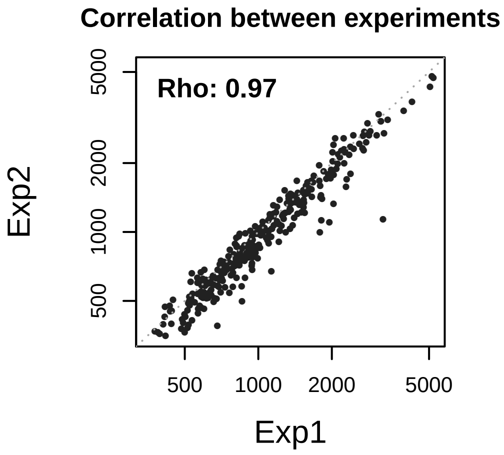

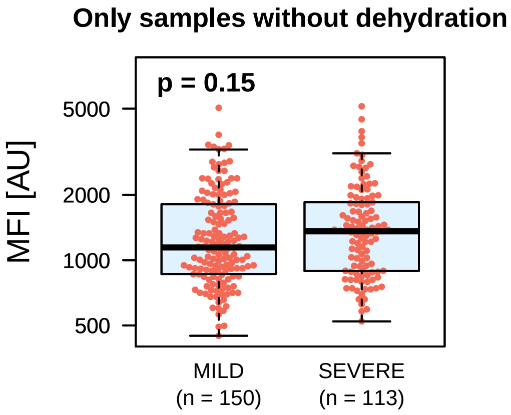

Supplement: Supplementary file 9 — Additional file 9. Information about proteins with divergent levels between mild and severe malaria patients. One panel for each of the 37 proteins presented as potential markers for separating mild malaria from severe malaria cases with a p-value < 0.05. The panel includes one boxplot with protein levels for the two groups and p-values together with the Spearman’s correlation Rho for that protein between the two experiments (Exp 1 and Exp 2). The second boxplot illustrate the protein profile per sample group but only including the samples that did not show signs of dehydration. [file 12936_2018_2576_MOESM9_ESM.pdf]
