# Supplementary material for: Levels of human proteins in plasma associated with acute paediatric malaria
Source: Malar J. 2018 Nov 15;17:426. doi: 10.1186/s12936-018-2576-y (PMC6238294; doi:10.1186/s12936-018-2576-y)

Additional file 10. Information about proteins with divergent levels between febrile convulsion compared to cerebral malaria patients

ANK1

Ankyrin 1
Antibody: HPA004842

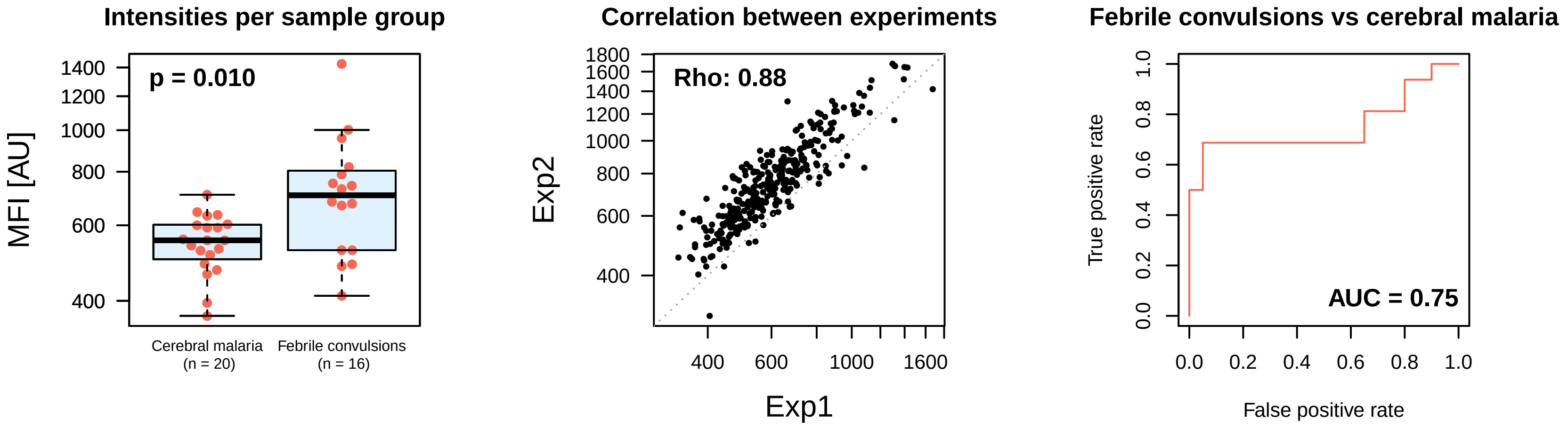

CD14

CD14 molecule
Antibody: HPA001887

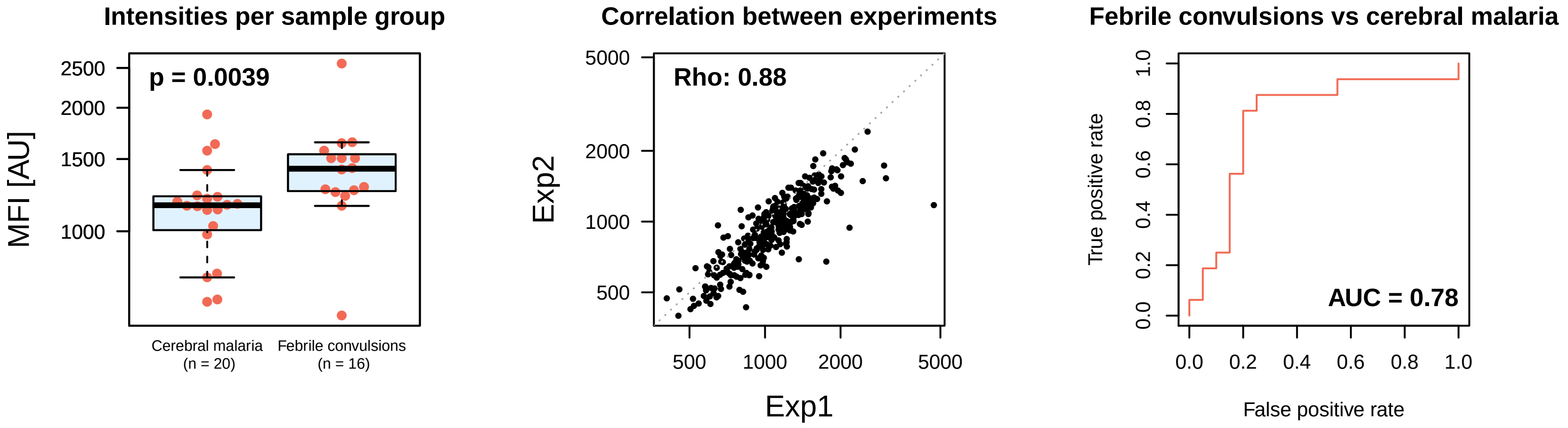

CDK14

Cyclin dependent kinase 14
Antibody: HPA015267

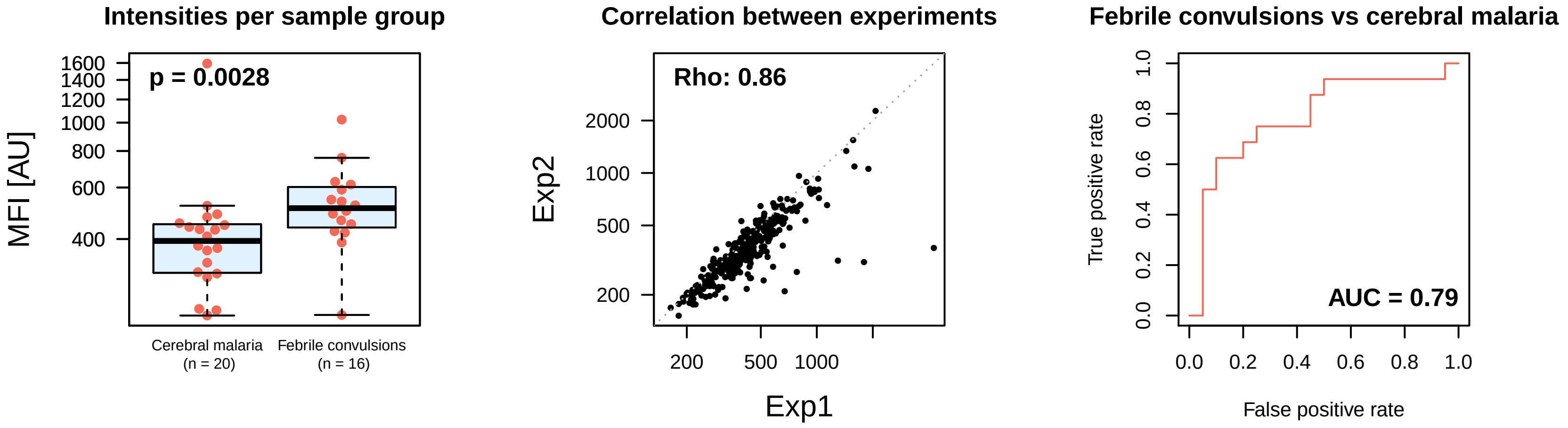

ELANE

Neutrophil elastase
Antibody: MAB91671 R&D

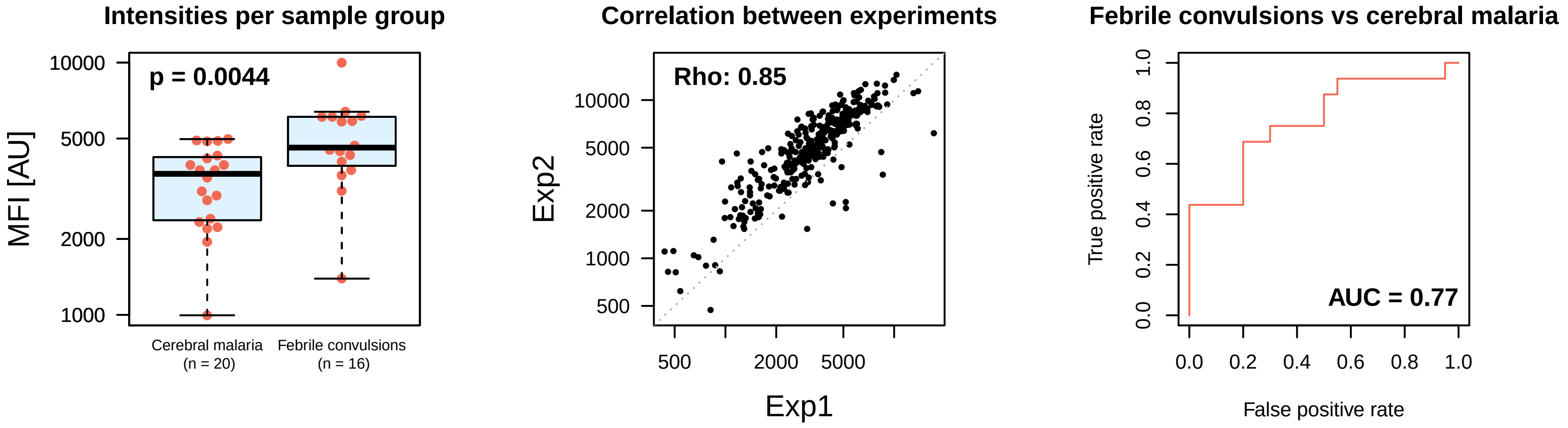

MPP1

Membrane palmitoylated protein 1
Antibody: HPA076675

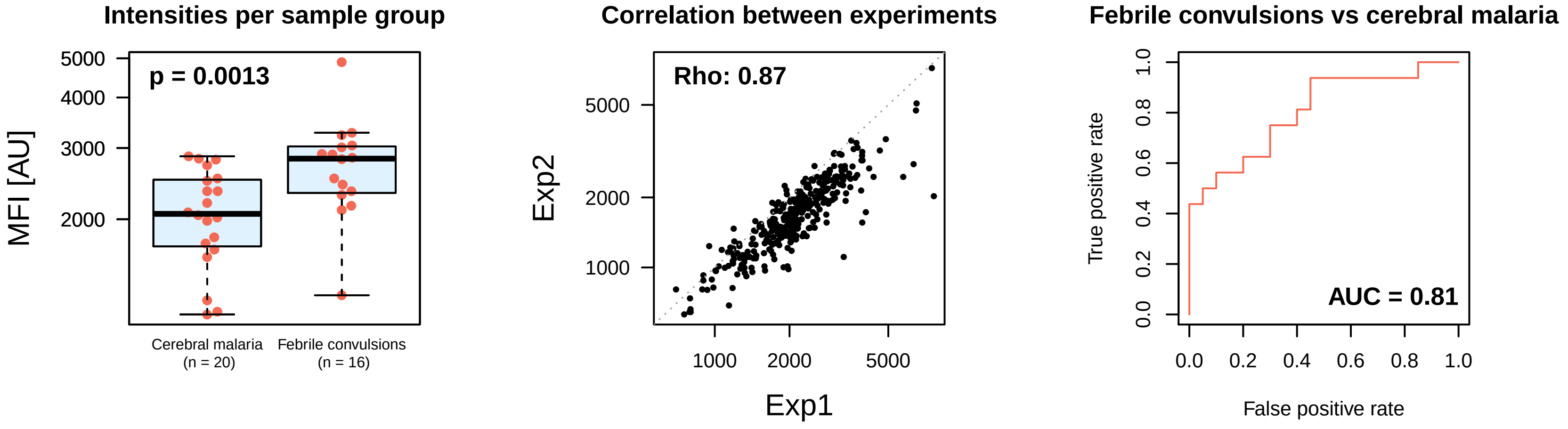

Supplement: Supplementary file 10 — Additional file 10. Information about proteins with divergent levels between febrile convulsion compared to cerebral malaria patients. One panel for each of the five proteins with a p-value < 0.01. Each panel includes a boxplot with protein levels for the group of patients with febrile convulsions and the group with cerebral malaria. The panel does also include the Spearman’s correlation Rho for that protein between the two experiments (Exp 1 and Exp 2) and the classification power of the protein represented on ROC curves (including AUC values). [file 12936_2018_2576_MOESM10_ESM.pdf]
