# Supplementary material for: Levels of human proteins in plasma associated with acute paediatric malaria
Source: Malar J. 2018 Nov 15;17:426. doi: 10.1186/s12936-018-2576-y (PMC6238294; doi:10.1186/s12936-018-2576-y)
Supplement: Supplementary file 11 — Additional file 11. Spearman correlation’s for antibodies targetting the same protein. Table of presented antibodies targetting the same protein with Spearman’s Rho > 0.70. The table also includes the gene and gene description of the target protein of the antibodies. [file 12936_2018_2576_MOESM11_ESM.pdf]

Additional file 11. Spearman correlation's for antibodies targeting the same protein

Gene	UniprotID	Gene description	Antibody 1	Antibody 2	Rho
AGT	P01019	Angiotensinogen	HPA001557	MAB3156 R&D	0.89
CD14	P08571	CD14 molecule	HPA001887	HPA002127	0.88
CD86	P42081	CD86 molecule	HPA012286	HPA012504	0.70
			HPA012286	HPA060986	0.78
			HPA012504	HPA060986	0.70
CRP	P02741	C-reactive protein	DY1707 R&D	HPA027396	0.73
CSF1	P09603	Colony stimulating factor 1	HPA022244	HPA044339	0.83
			HPA022244	HPA061864	0.87
			HPA044339	HPA061864	0.85
ETFB	P38117	Electron transfer flavoprotein beta subunit	HPA018898	HPA018921	0.74
GYPC	P04921	Glycophorin C (Gerbich blood group)	HPA009038	HPA047565	0.76
IGFBP1	P08833	Insulin like growth factor binding protein 1	DY871 R&D	HPA046972	0.88
			DY871 R&D	MAB675 R&D	0.98
			HPA046972	MAB675 R&D	0.87
LCP1	P13796	Lymphocyte cytosolic protein 1	HPA019493	HPA000895	0.88
RIPK2	O43353	Receptor interacting serine/threonine kinase 2	HPA015764	HPA016499	0.89
SERPINA3	P01011	Serpin family A member 3	HPA000893	HPA002560	0.96
VCAM1	P19320	Vascular cell adhesion molecule 1	HPA001618	HPA034795	0.88
			HPA034795	HPA069867	0.80
			HPA001618	HPA069867	0.88
VWF	P04275	von Willebrand factor	HPA001815	HPA002082	0.85
