# Supplementary material for: Levels of human proteins in plasma associated with acute paediatric malaria
Source: Malar J. 2018 Nov 15;17:426. doi: 10.1186/s12936-018-2576-y (PMC6238294; doi:10.1186/s12936-018-2576-y)

Additional file 13. Heatmap over relative protein intensities between cases with mild and severe malaria

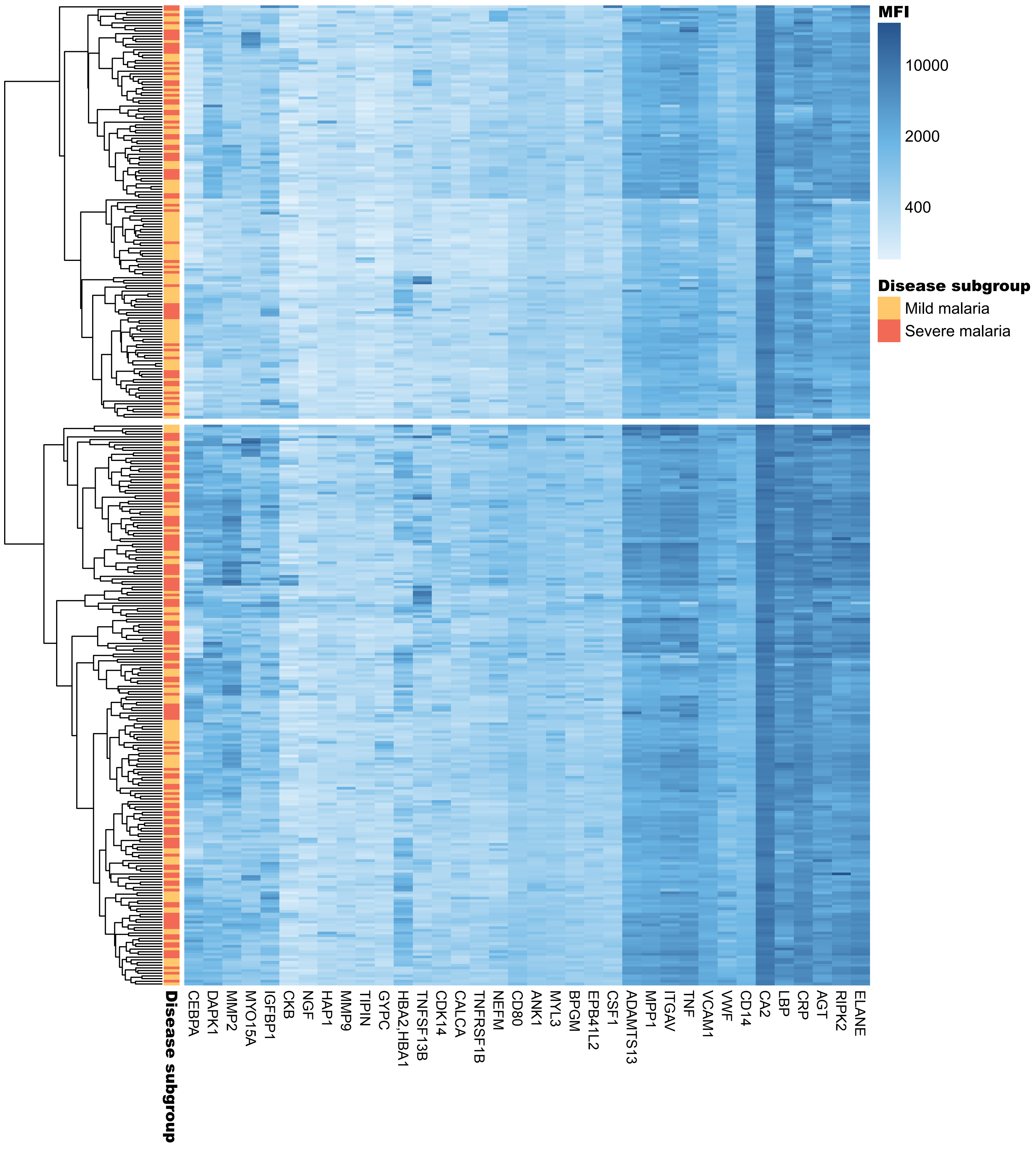

Supplement: Supplementary file 13 — Additional file 13. Heatmap over relative protein intensities between cases with mild and severe malaria. Exploratory hierarchical clustering of samples with mild and severe malaria for all proteins with p-values < 0.05 between these two groups. An extra sample-legend is provided for sample subgroup: mild malaria (orange) and severe malaria (red). [file 12936_2018_2576_MOESM13_ESM.pdf]
